# Supplementary figures and images for: Cryoelectrolysis—electrolytic processes in a frozen physiological saline medium
Source: PeerJ. 2017 Jan 17;5:e2810. doi: 10.7717/peerj.2810 (PMC5244893; doi:10.7717/peerj.2810)

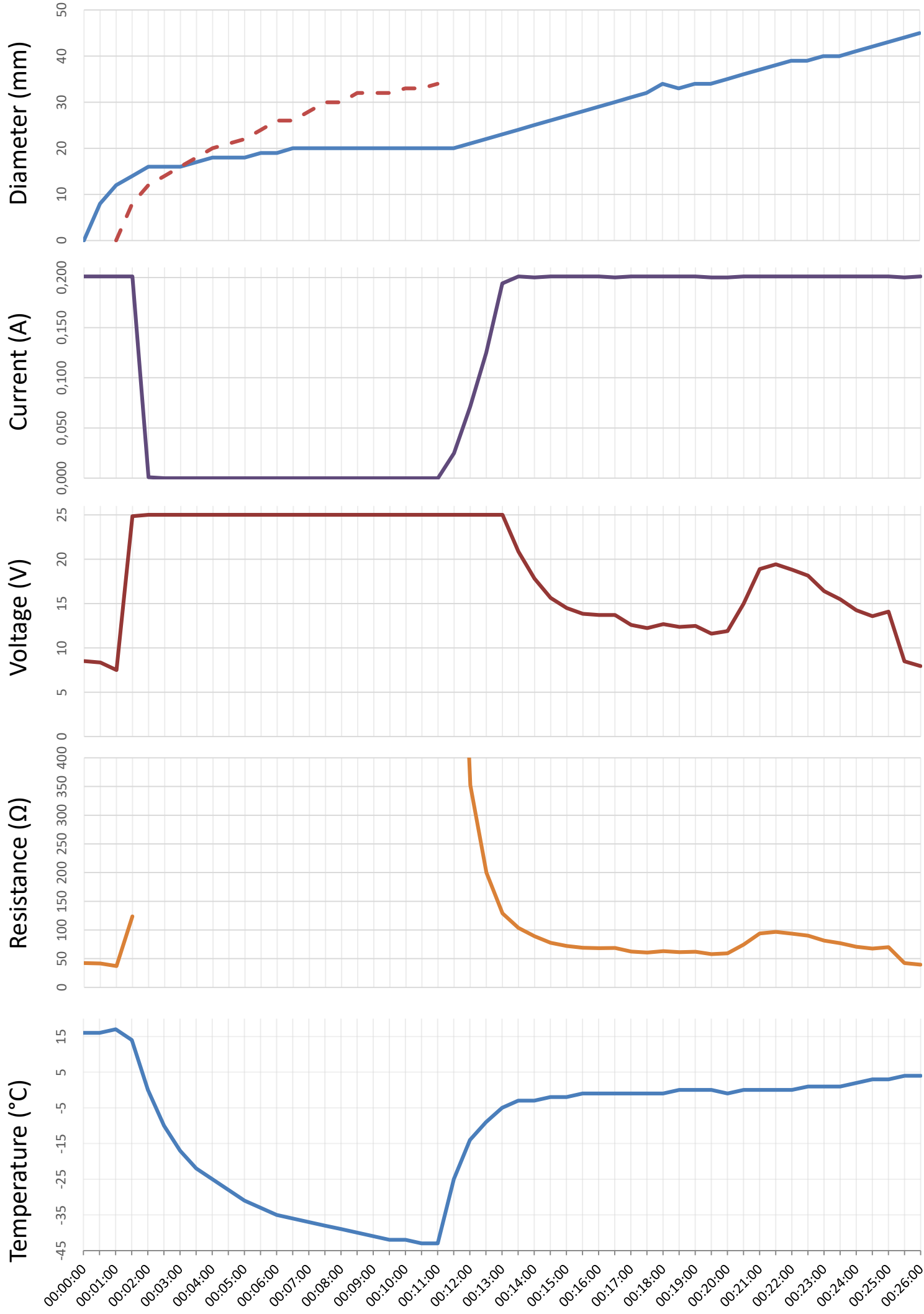

| time     | ∅ pH front (mm) | ∅ ice front (mm) |  | V (V) | I (A) | R (Ω) | T <sub>tip</sub> (°C) |
|----------|-----------------|------------------|--|-------|-------|-------|-----------------------|
| 00:00:00 | 0               |                  |  | 9     | 0,201 | 42    | 16                    |
| 00:00:30 | 8               |                  |  | 8     | 0,201 | 42    | 16                    |
| 00:01:00 | 12              | 0                |  | 7     | 0,201 | 37    | 17                    |
| 00:01:30 | 14              | 8                |  | 25    | 0,201 | 124   | 14                    |
| 00:02:00 | 16              | 12               |  | 25    | 0,001 |       | 0                     |
| 00:02:30 | 16              | 14               |  | 25    | 0,000 |       | -10                   |
| 00:03:00 | 16              | 16               |  | 25    | 0,000 |       | -17                   |
| 00:03:30 | 17              | 18               |  | 25    | 0,000 |       | -22                   |
| 00:04:00 | 18              | 20               |  | 25    | 0,000 |       | -25                   |
| 00:04:30 | 18              | 21               |  | 25    | 0,000 |       | -28                   |
| 00:05:00 | 18              | 22               |  | 25    | 0,000 |       | -31                   |
| 00:05:30 | 19              | 24               |  | 25    | 0,000 |       | -33                   |
| 00:06:00 | 19              | 26               |  | 25    | 0,000 |       | -35                   |
| 00:06:30 | 20              | 26               |  | 25    | 0,000 |       | -36                   |
| 00:07:00 | 20              | 28               |  | 25    | 0,000 |       | -37                   |
| 00:07:30 | 20              | 30               |  | 25    | 0,000 |       | -38                   |
| 00:08:00 | 20              | 30               |  | 25    | 0,000 |       | -39                   |
| 00:08:30 | 20              | 32               |  | 25    | 0,000 |       | -40                   |
| 00:09:00 | 20              | 32               |  | 25    | 0,000 |       | -41                   |
| 00:09:30 | 20              | 32               |  | 25    | 0,000 |       | -42                   |
| 00:10:00 | 20              | 33               |  | 25    | 0,000 |       | -42                   |
| 00:10:30 | 20              | 33               |  | 25    | 0,000 |       | -43                   |
| 00:11:00 | 20              | 34               |  | 25    | 0,000 |       | -43                   |
| 00:11:30 | 20              |                  |  | 25    | 0,025 | 1000  | -25                   |
| 00:12:00 | 21              |                  |  | 25    | 0,071 | 352   | -14                   |
| 00:12:30 | 22              |                  |  | 25    | 0,125 | 200   | -9                    |
| 00:13:00 | 23              |                  |  | 25    | 0,194 | 129   | -5                    |
| 00:13:30 | 24              |                  |  | 21    | 0,201 | 104   | -3                    |
| 00:14:00 | 25              |                  |  | 18    | 0,200 | 89    | -3                    |
| 00:14:30 | 26              |                  |  | 16    | 0,201 | 78    | -2                    |
| 00:15:00 | 27              |                  |  | 15    | 0,201 | 72    | -2                    |
| 00:15:30 | 28              |                  |  | 14    | 0,201 | 69    | -1                    |
| 00:16:00 | 29              |                  |  | 14    | 0,201 | 68    | -1                    |
| 00:16:30 | 30              |                  |  | 14    | 0,200 | 69    | -1                    |
| 00:17:00 | 31              |                  |  | 13    | 0,201 | 63    | -1                    |
| 00:17:30 | 32              |                  |  | 12    | 0,201 | 61    | -1                    |
| 00:18:00 | 34              |                  |  | 13    | 0,201 | 63    | -1                    |
| 00:18:30 | 33              |                  |  | 12    | 0,201 | 61    | 0                     |
| 00:19:00 | 34              |                  |  | 12    | 0,201 | 62    | 0                     |
| 00:19:30 | 34              |                  |  | 12    | 0,200 | 58    | 0                     |
| 00:20:00 | 35              |                  |  | 12    | 0,200 | 59    | -1                    |
| 00:20:30 | 36              |                  |  | 15    | 0,201 | 75    | 0                     |
| 00:21:00 | 37              |                  |  | 19    | 0,201 | 94    | 0                     |
| 00:21:30 | 38              |                  |  | 19    | 0,201 | 97    | 0                     |
| 00:22:00 | 39              |                  |  | 19    | 0,201 | 94    | 0                     |
| 00:22:30 | 39              |                  |  | 18    | 0,201 | 90    | 1                     |
| 00:23:00 | 40              |                  |  | 16    | 0,201 | 82    | 1                     |
| 00:23:30 | 40              |                  |  | 15    | 0,201 | 77    | 1                     |

|          |    |  |  |    |       |    |   |
|----------|----|--|--|----|-------|----|---|
| 00:24:00 | 41 |  |  | 14 | 0,201 | 71 | 2 |
| 00:24:30 | 42 |  |  | 14 | 0,201 | 68 | 3 |
| 00:25:00 | 43 |  |  | 14 | 0,201 | 70 | 3 |
| 00:25:30 | 44 |  |  | 8  | 0,200 | 42 | 4 |
| 00:26:00 | 45 |  |  | 8  | 0,201 | 40 | 4 |

Supplement: Supplemental Information 1 — The second page shows the raw data from which we built the graphs. The entire experiment lasted 26 minutes. [file peerj-05-2810-s001.pdf]

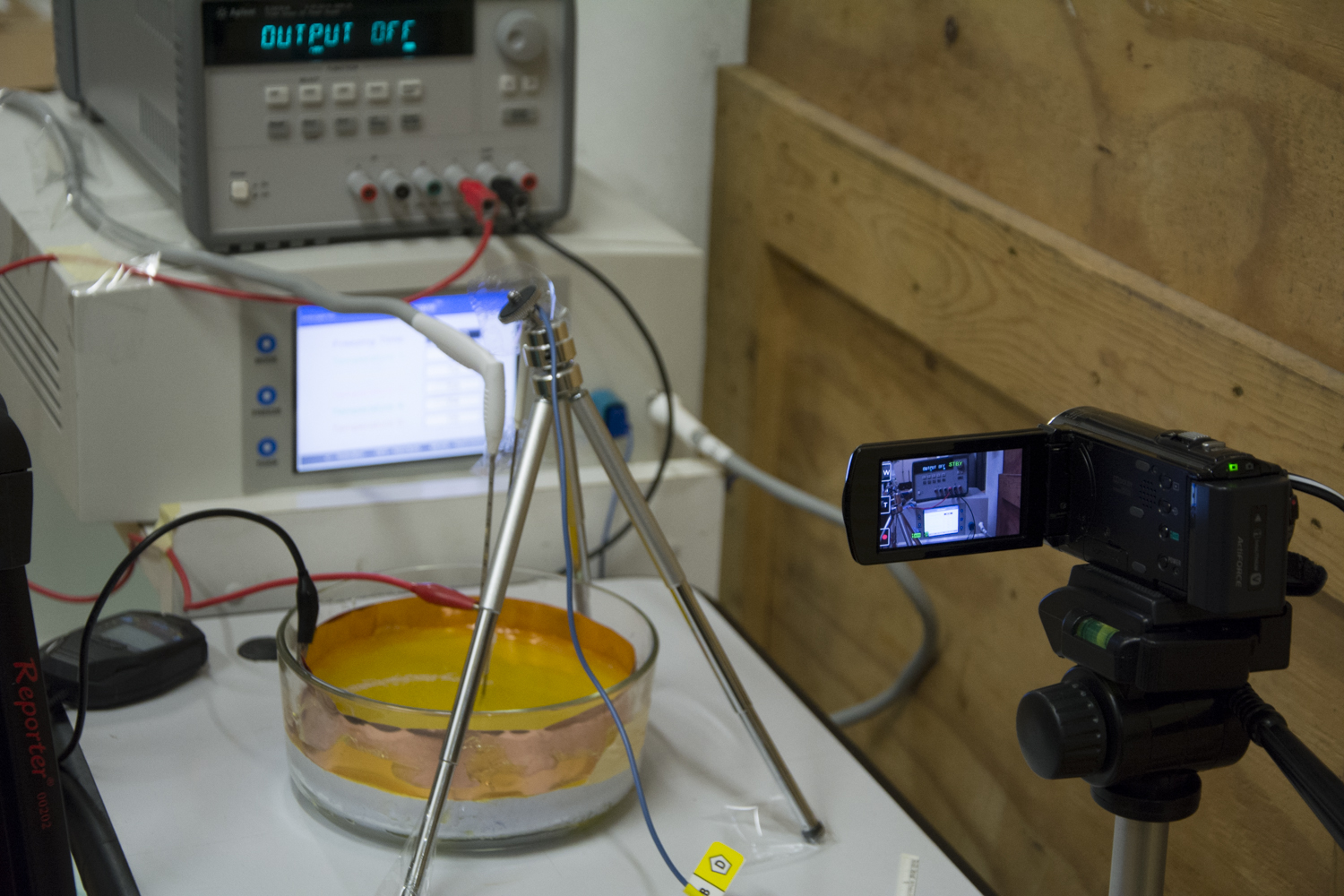

Supplement: Supplemental Information 2 — In particular: 160720-015-Exp_Cryoelettro shows details of the experiment, with camera, suppliers, probe and saline agar solution; 160720-016-Exp_Cryoelettro is a closer view of the setup. Photos from 160720-030-Exp_Cryoelettro to 160720-055-Exp_Cryoelettro shows the progression mentioned before from minute 0:00 to minute 12:30; each photo is taken every 30 s. [file peerj-05-2810-s002.zip › PeerJ1/160720-015-Exp_Cryoelettro.jpg]

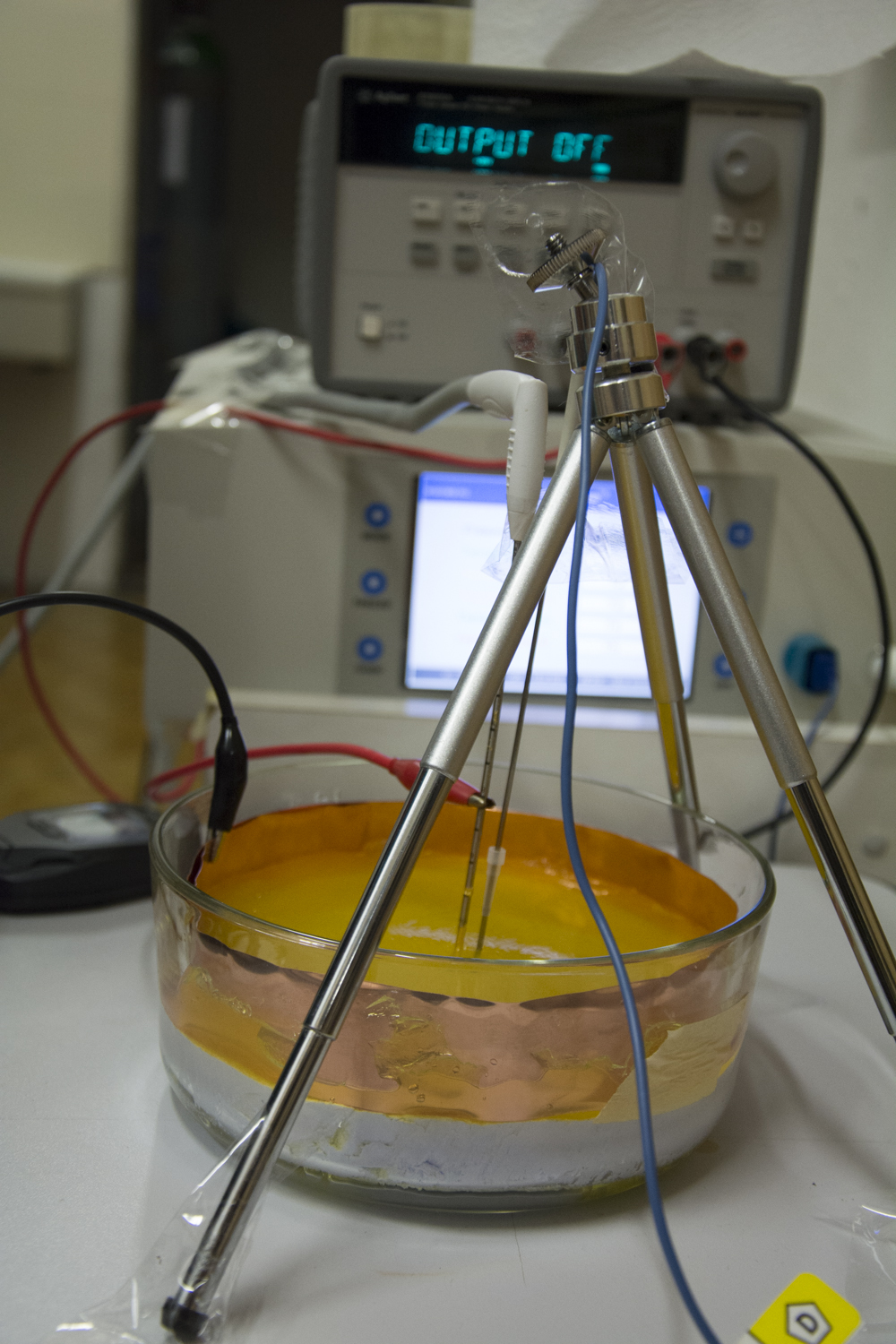

Supplement: Supplemental Information 2 — In particular: 160720-015-Exp_Cryoelettro shows details of the experiment, with camera, suppliers, probe and saline agar solution; 160720-016-Exp_Cryoelettro is a closer view of the setup. Photos from 160720-030-Exp_Cryoelettro to 160720-055-Exp_Cryoelettro shows the progression mentioned before from minute 0:00 to minute 12:30; each photo is taken every 30 s. [file peerj-05-2810-s002.zip › PeerJ1/160720-016-Exp_Cryoelettro.jpg]

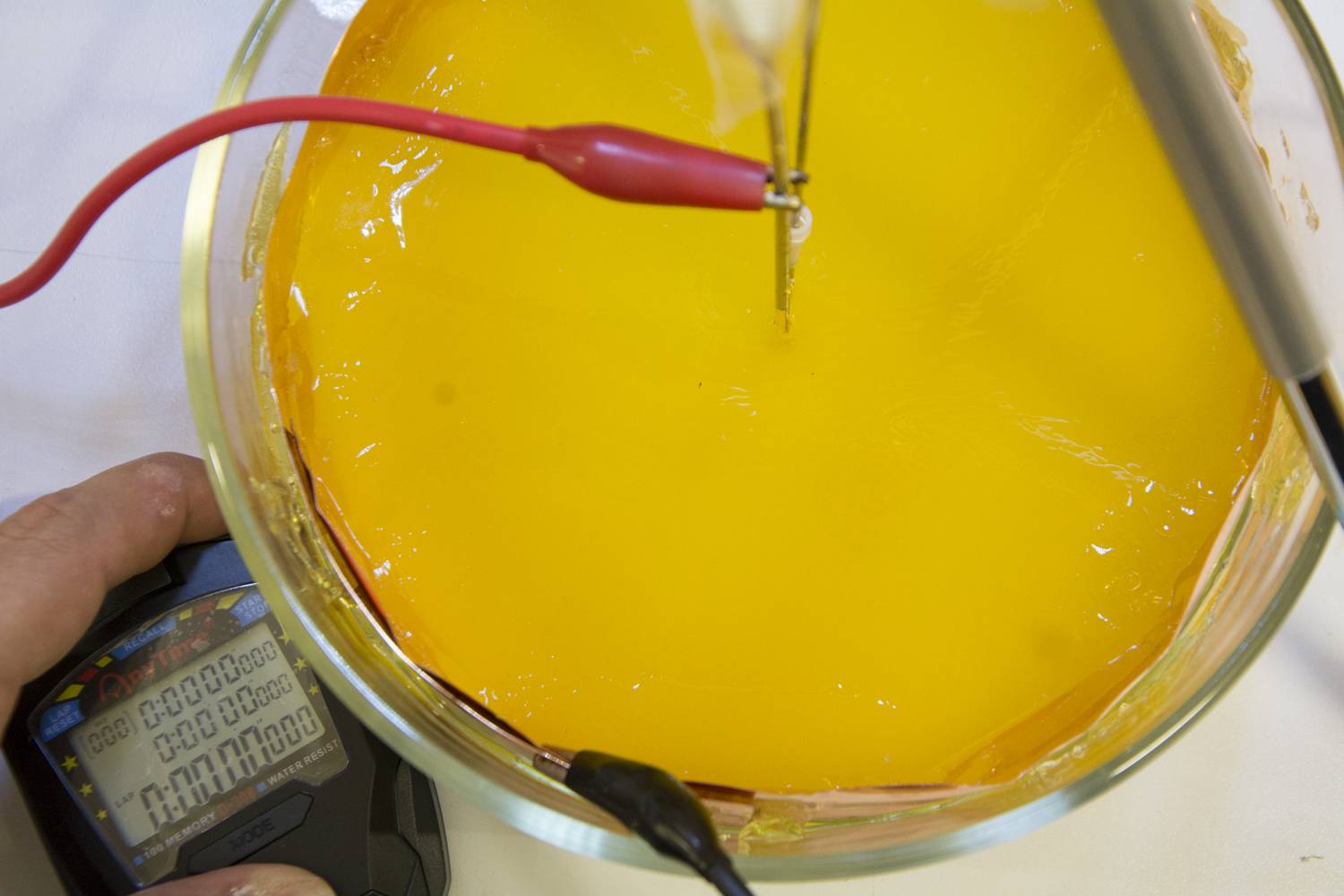

Supplement: Supplemental Information 2 — In particular: 160720-015-Exp_Cryoelettro shows details of the experiment, with camera, suppliers, probe and saline agar solution; 160720-016-Exp_Cryoelettro is a closer view of the setup. Photos from 160720-030-Exp_Cryoelettro to 160720-055-Exp_Cryoelettro shows the progression mentioned before from minute 0:00 to minute 12:30; each photo is taken every 30 s. [file peerj-05-2810-s002.zip › PeerJ1/160720-030-Exp_Cryoelettro.jpg]

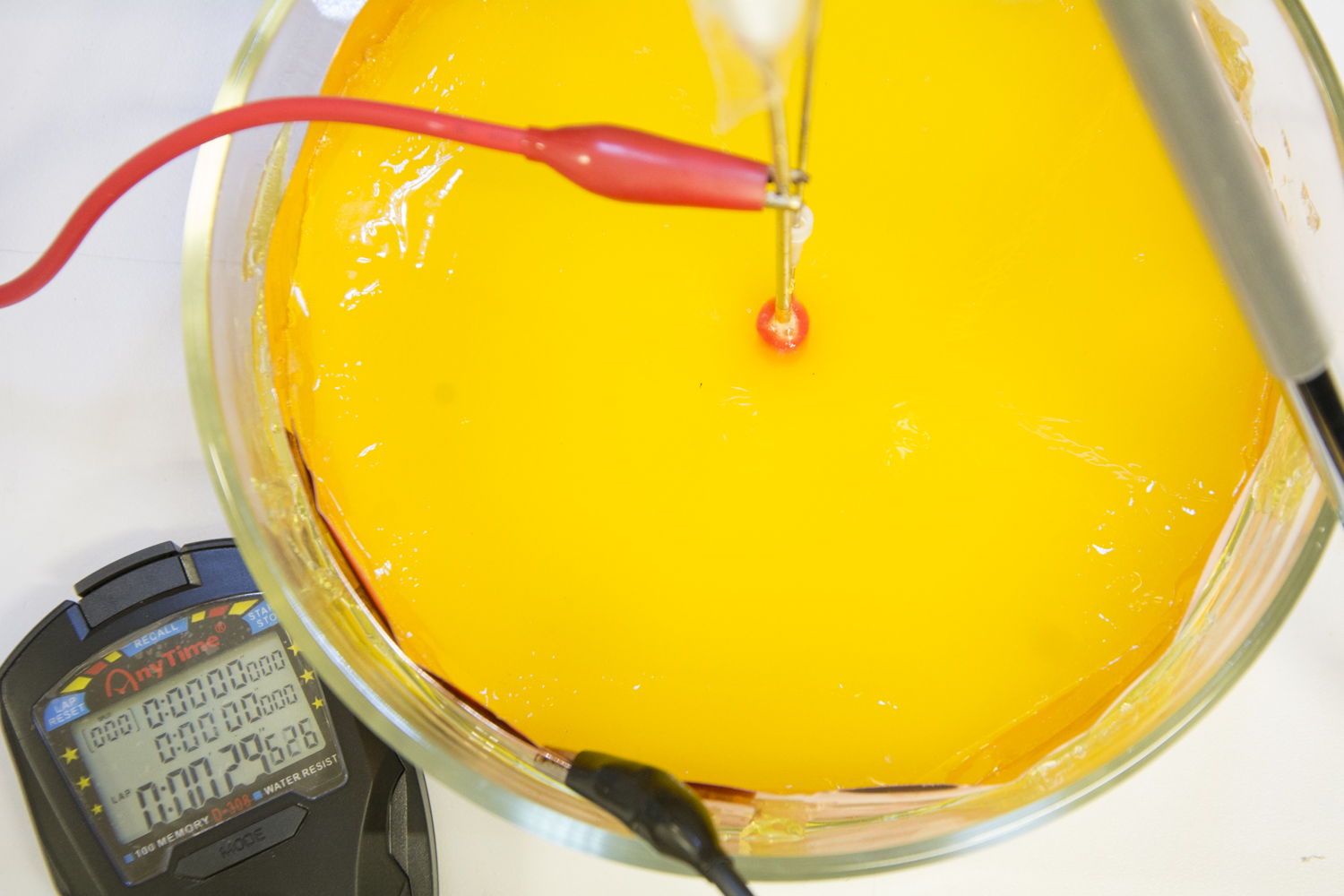

Supplement: Supplemental Information 2 — In particular: 160720-015-Exp_Cryoelettro shows details of the experiment, with camera, suppliers, probe and saline agar solution; 160720-016-Exp_Cryoelettro is a closer view of the setup. Photos from 160720-030-Exp_Cryoelettro to 160720-055-Exp_Cryoelettro shows the progression mentioned before from minute 0:00 to minute 12:30; each photo is taken every 30 s. [file peerj-05-2810-s002.zip › PeerJ1/160720-031-Exp_Cryoelettro.jpg]

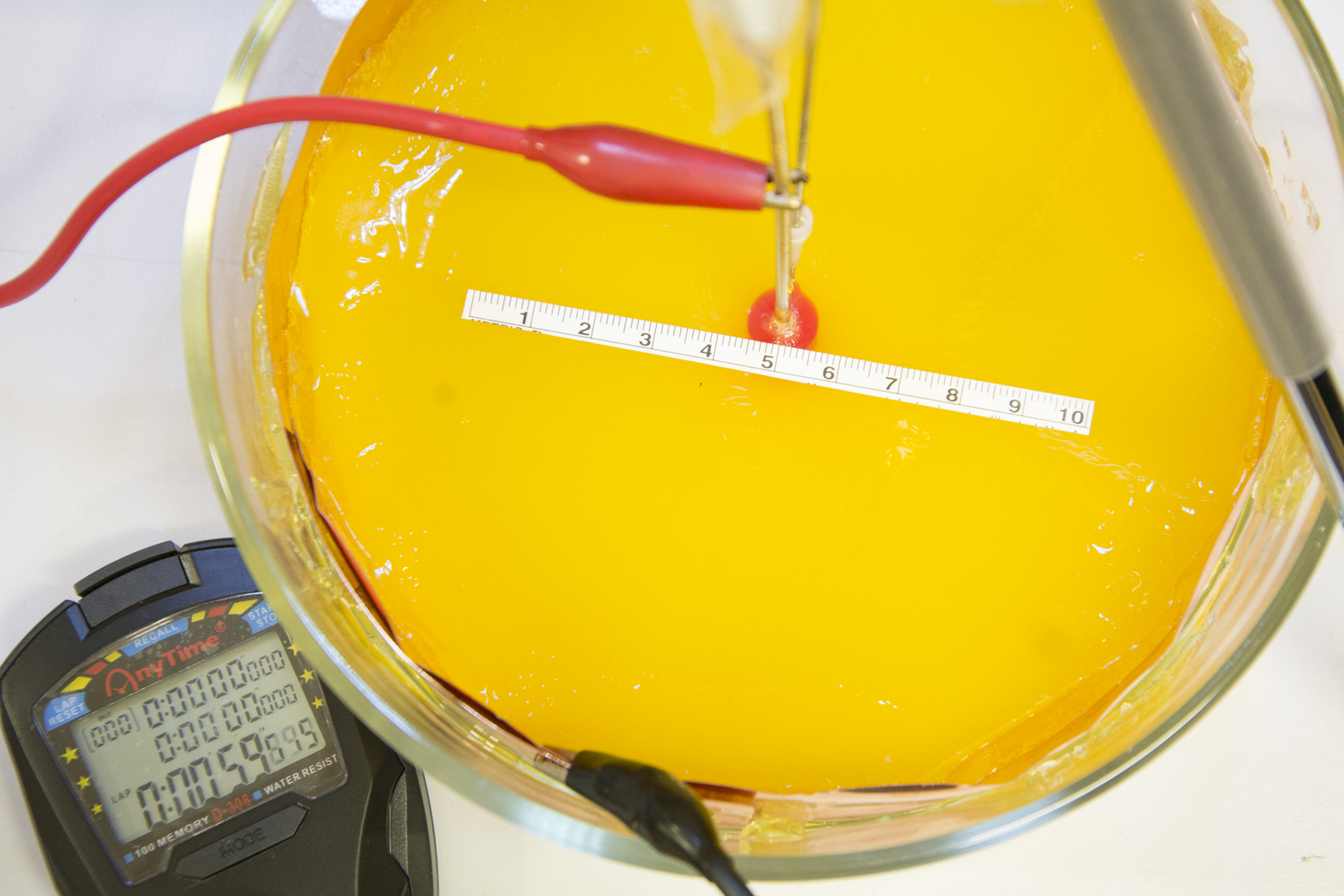

Supplement: Supplemental Information 2 — In particular: 160720-015-Exp_Cryoelettro shows details of the experiment, with camera, suppliers, probe and saline agar solution; 160720-016-Exp_Cryoelettro is a closer view of the setup. Photos from 160720-030-Exp_Cryoelettro to 160720-055-Exp_Cryoelettro shows the progression mentioned before from minute 0:00 to minute 12:30; each photo is taken every 30 s. [file peerj-05-2810-s002.zip › PeerJ1/160720-032-Exp_Cryoelettro.jpg]

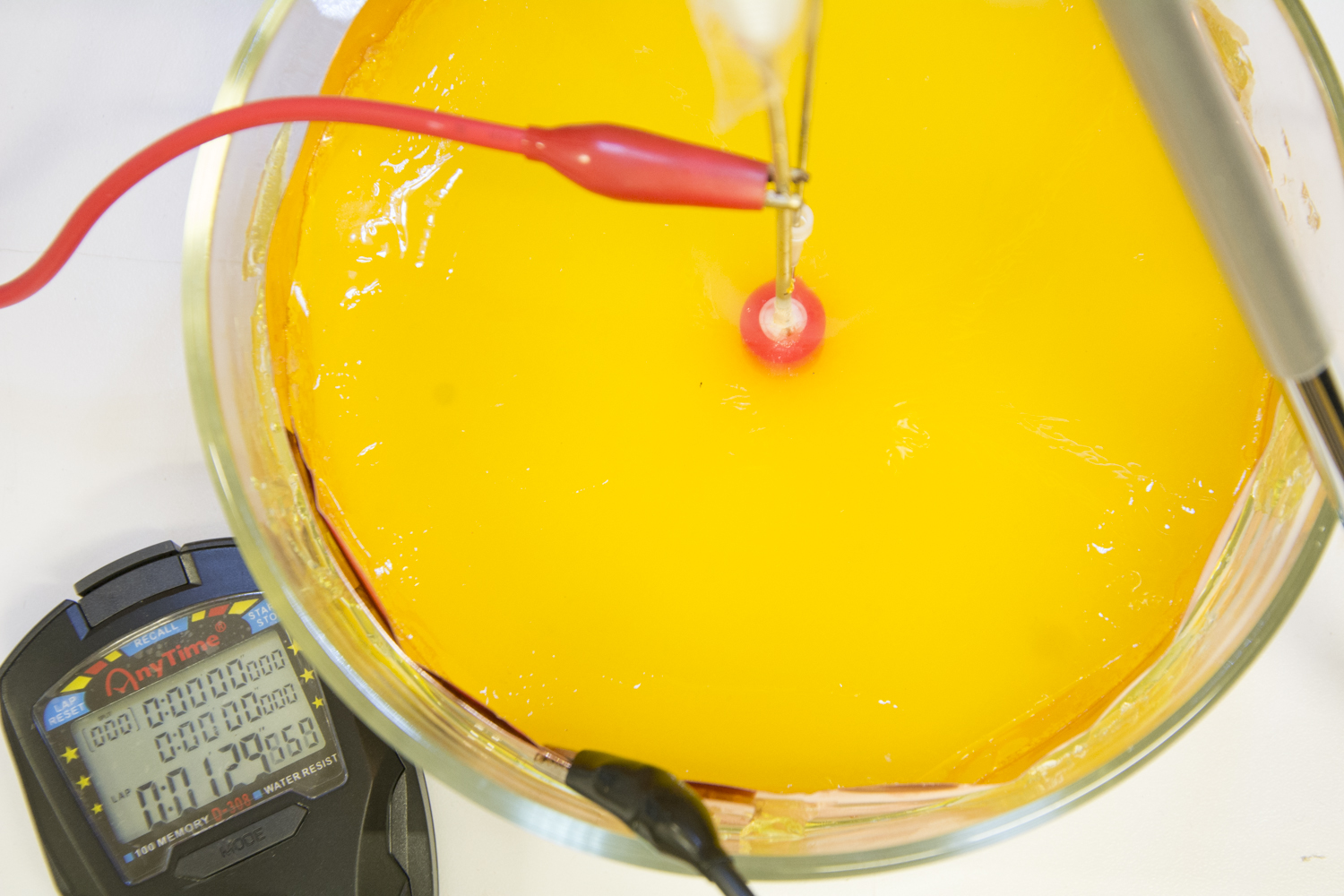

Supplement: Supplemental Information 2 — In particular: 160720-015-Exp_Cryoelettro shows details of the experiment, with camera, suppliers, probe and saline agar solution; 160720-016-Exp_Cryoelettro is a closer view of the setup. Photos from 160720-030-Exp_Cryoelettro to 160720-055-Exp_Cryoelettro shows the progression mentioned before from minute 0:00 to minute 12:30; each photo is taken every 30 s. [file peerj-05-2810-s002.zip › PeerJ1/160720-033-Exp_Cryoelettro.jpg]

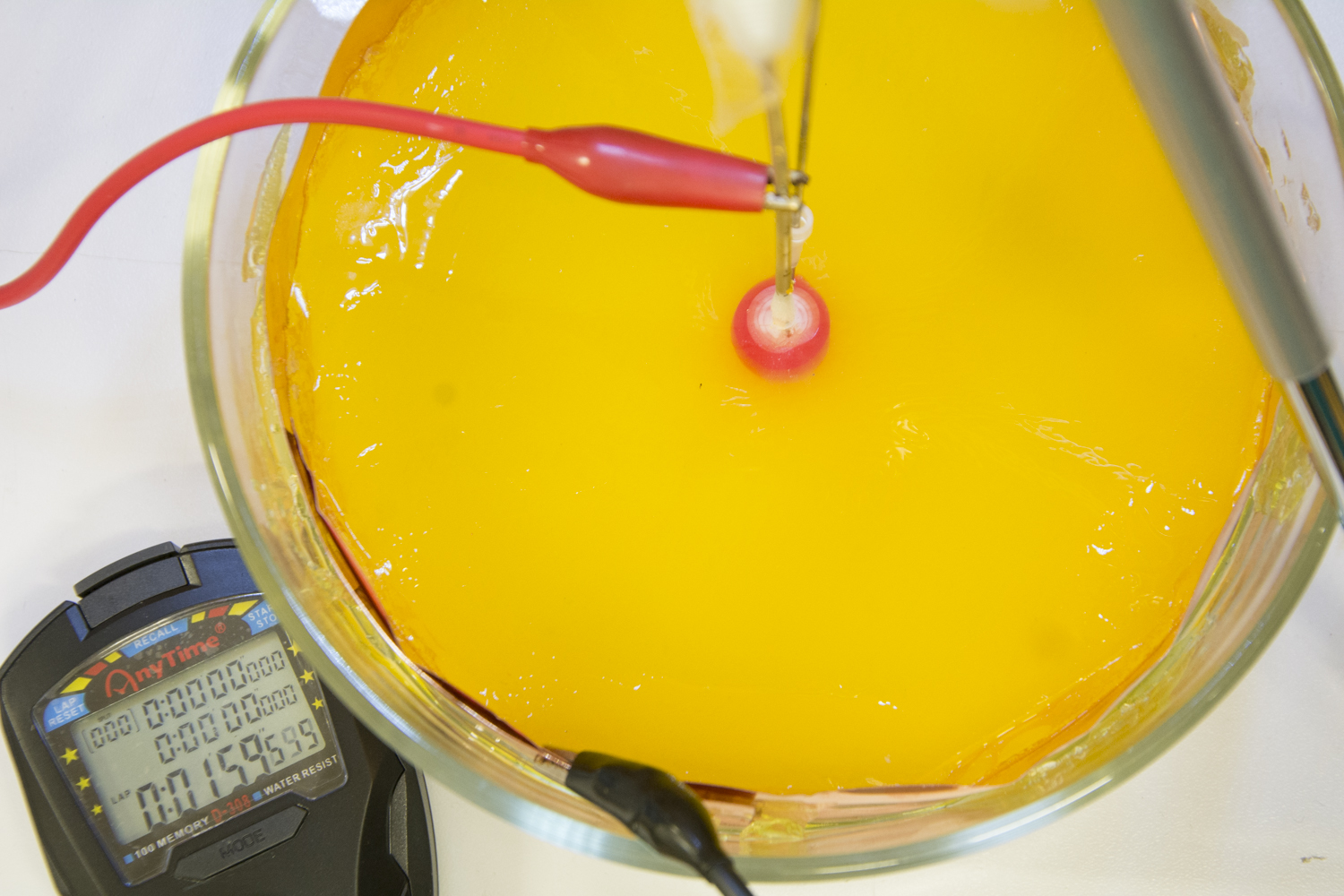

Supplement: Supplemental Information 2 — In particular: 160720-015-Exp_Cryoelettro shows details of the experiment, with camera, suppliers, probe and saline agar solution; 160720-016-Exp_Cryoelettro is a closer view of the setup. Photos from 160720-030-Exp_Cryoelettro to 160720-055-Exp_Cryoelettro shows the progression mentioned before from minute 0:00 to minute 12:30; each photo is taken every 30 s. [file peerj-05-2810-s002.zip › PeerJ1/160720-034-Exp_Cryoelettro.jpg]

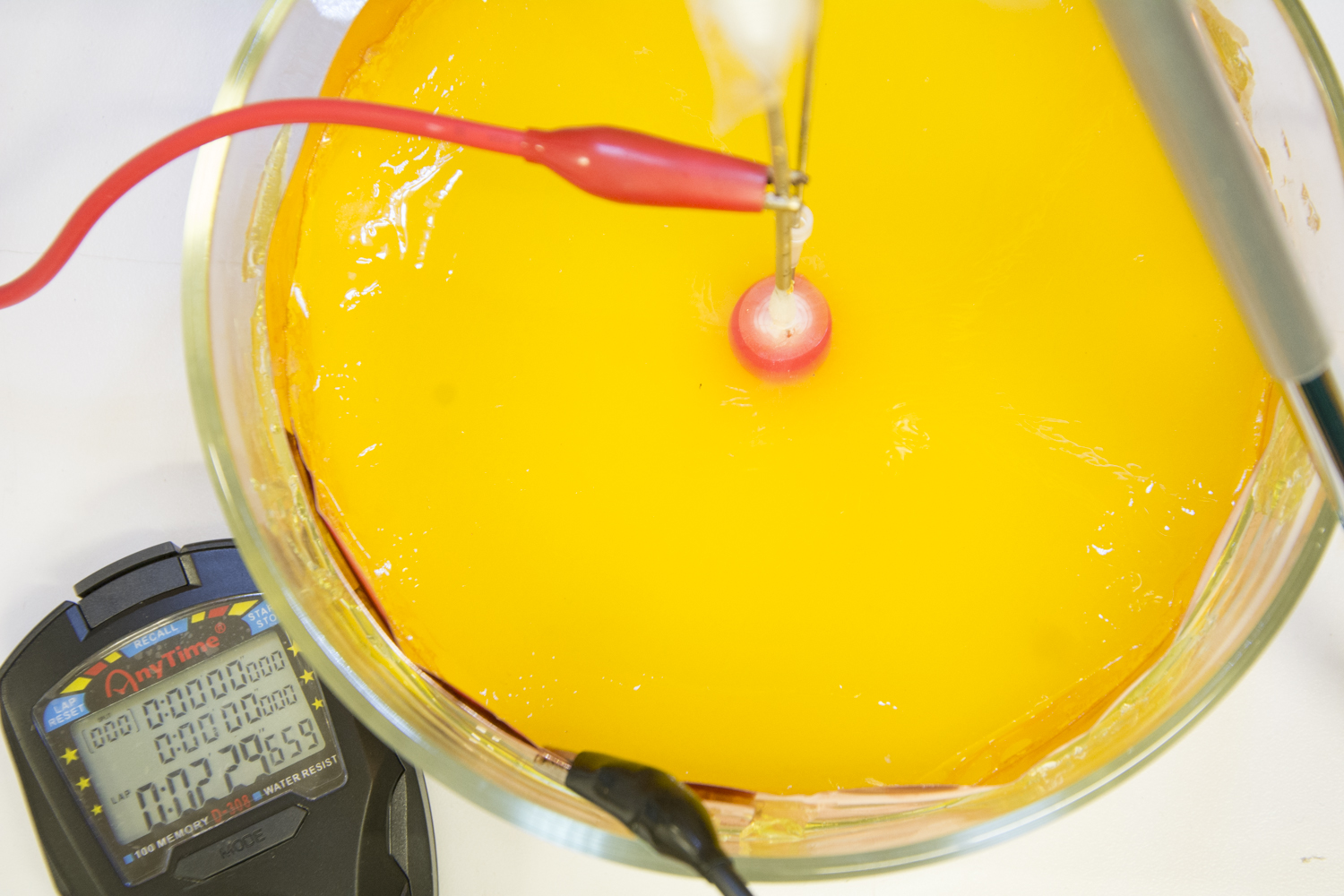

Supplement: Supplemental Information 2 — In particular: 160720-015-Exp_Cryoelettro shows details of the experiment, with camera, suppliers, probe and saline agar solution; 160720-016-Exp_Cryoelettro is a closer view of the setup. Photos from 160720-030-Exp_Cryoelettro to 160720-055-Exp_Cryoelettro shows the progression mentioned before from minute 0:00 to minute 12:30; each photo is taken every 30 s. [file peerj-05-2810-s002.zip › PeerJ1/160720-035-Exp_Cryoelettro.jpg]

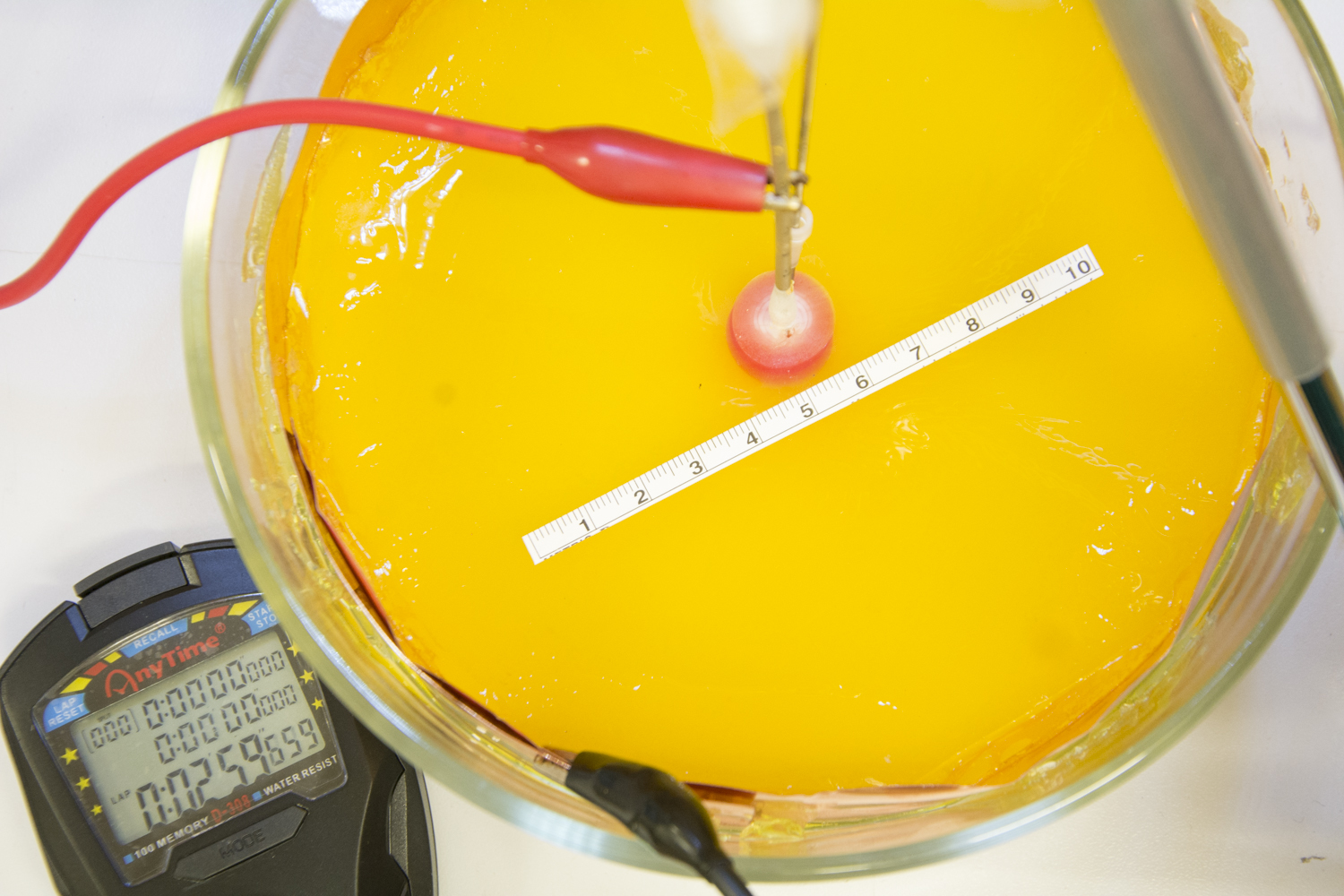

Supplement: Supplemental Information 2 — In particular: 160720-015-Exp_Cryoelettro shows details of the experiment, with camera, suppliers, probe and saline agar solution; 160720-016-Exp_Cryoelettro is a closer view of the setup. Photos from 160720-030-Exp_Cryoelettro to 160720-055-Exp_Cryoelettro shows the progression mentioned before from minute 0:00 to minute 12:30; each photo is taken every 30 s. [file peerj-05-2810-s002.zip › PeerJ1/160720-036-Exp_Cryoelettro.jpg]

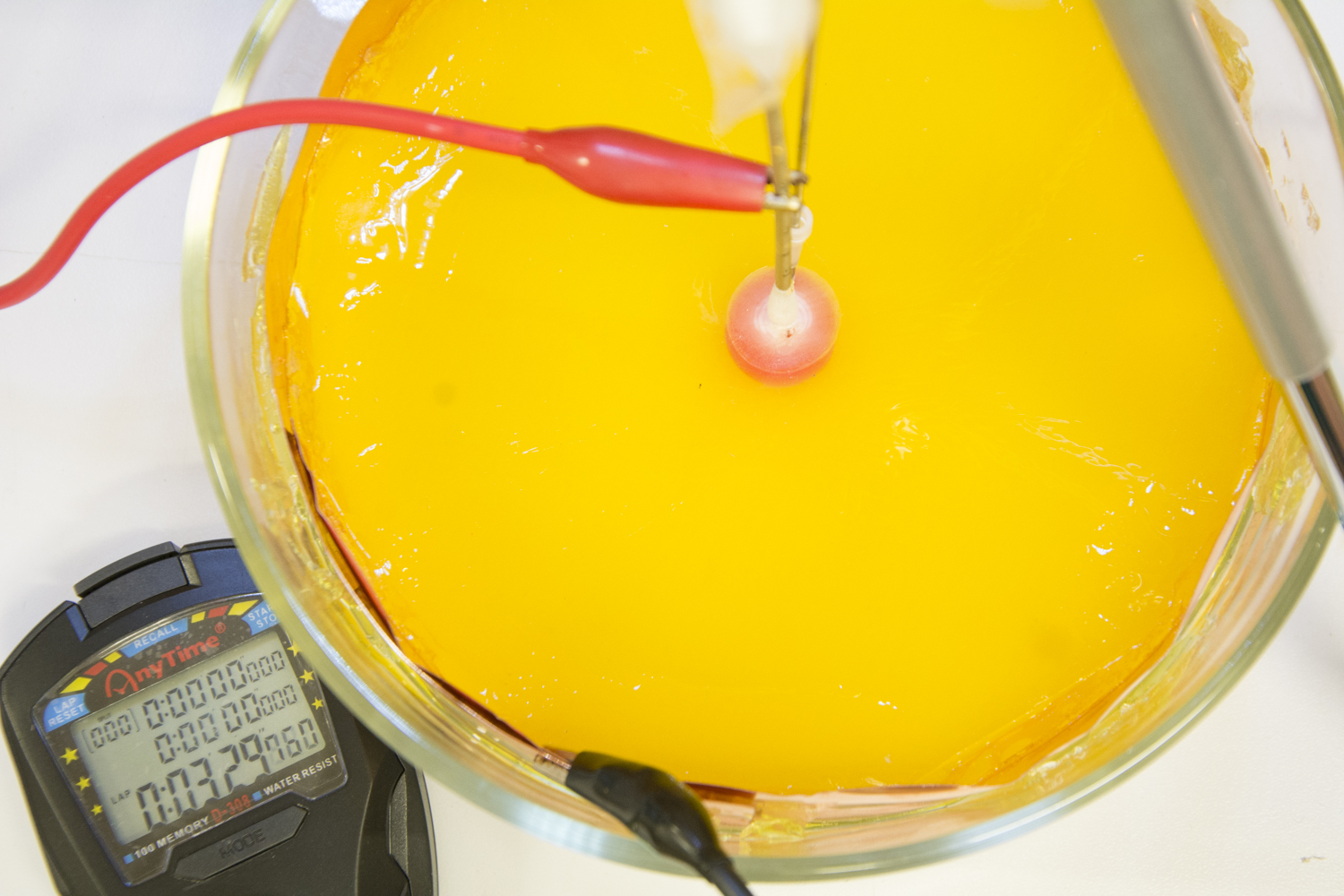

Supplement: Supplemental Information 2 — In particular: 160720-015-Exp_Cryoelettro shows details of the experiment, with camera, suppliers, probe and saline agar solution; 160720-016-Exp_Cryoelettro is a closer view of the setup. Photos from 160720-030-Exp_Cryoelettro to 160720-055-Exp_Cryoelettro shows the progression mentioned before from minute 0:00 to minute 12:30; each photo is taken every 30 s. [file peerj-05-2810-s002.zip › PeerJ1/160720-037-Exp_Cryoelettro.jpg]

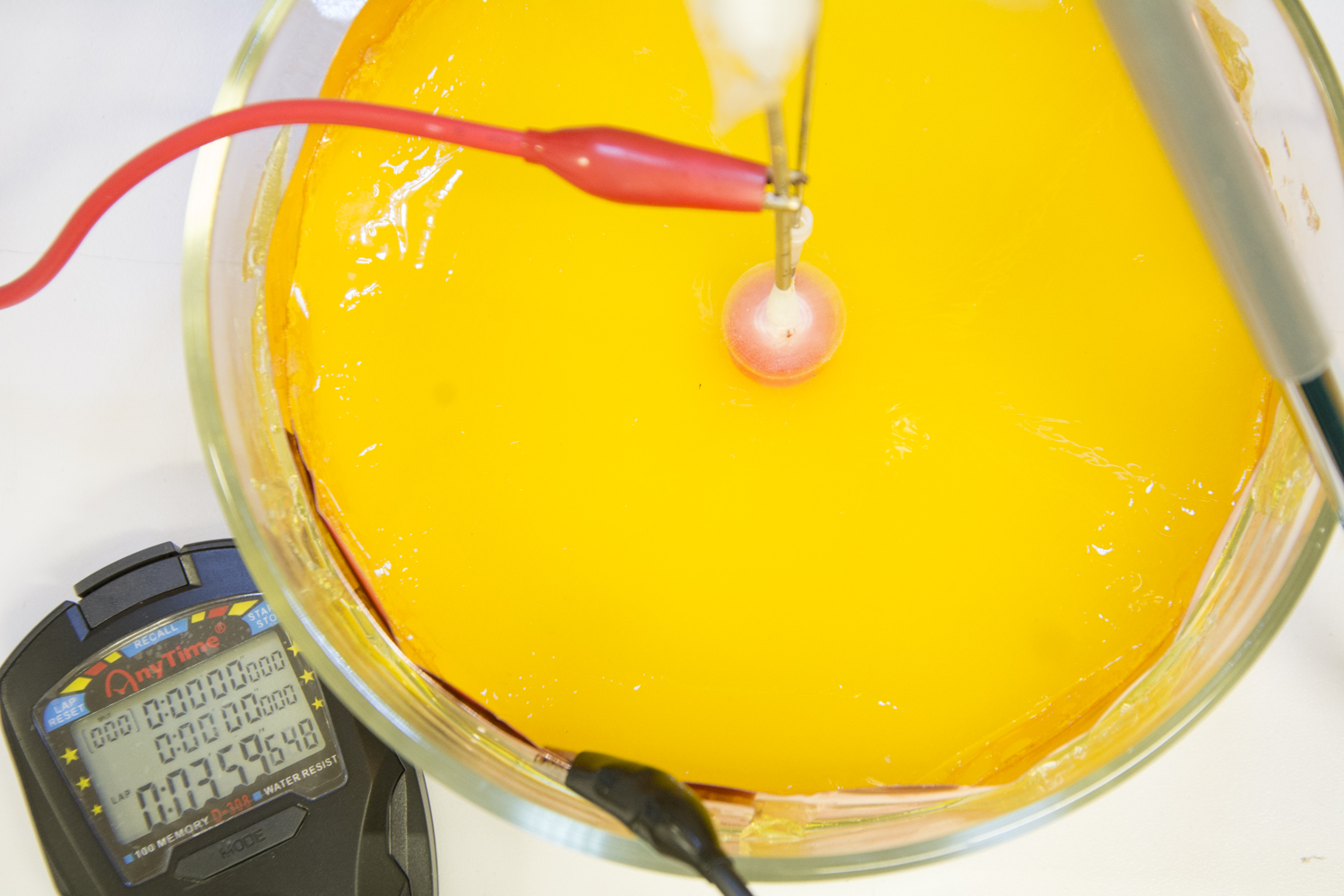

Supplement: Supplemental Information 2 — In particular: 160720-015-Exp_Cryoelettro shows details of the experiment, with camera, suppliers, probe and saline agar solution; 160720-016-Exp_Cryoelettro is a closer view of the setup. Photos from 160720-030-Exp_Cryoelettro to 160720-055-Exp_Cryoelettro shows the progression mentioned before from minute 0:00 to minute 12:30; each photo is taken every 30 s. [file peerj-05-2810-s002.zip › PeerJ1/160720-038-Exp_Cryoelettro.jpg]

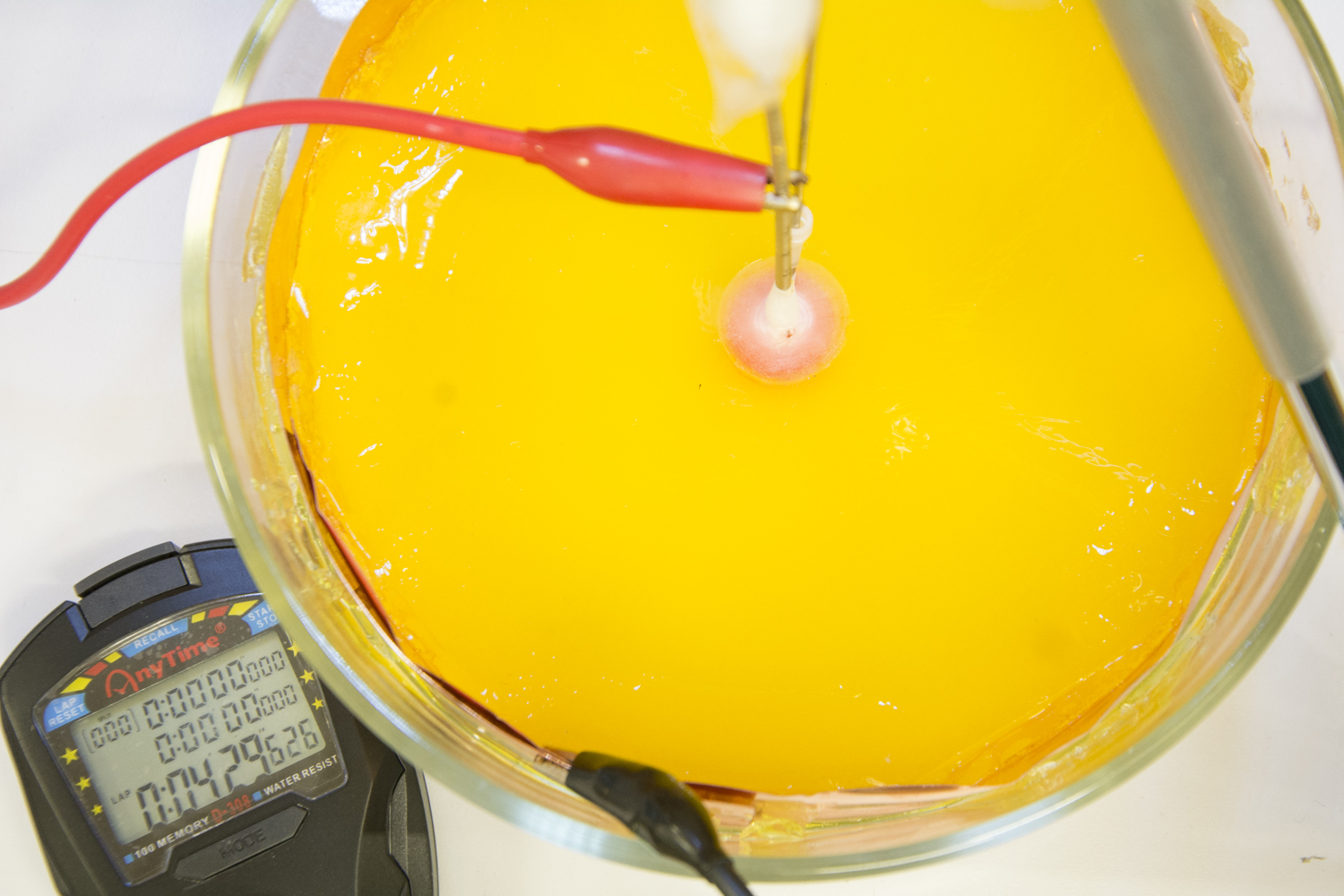

Supplement: Supplemental Information 2 — In particular: 160720-015-Exp_Cryoelettro shows details of the experiment, with camera, suppliers, probe and saline agar solution; 160720-016-Exp_Cryoelettro is a closer view of the setup. Photos from 160720-030-Exp_Cryoelettro to 160720-055-Exp_Cryoelettro shows the progression mentioned before from minute 0:00 to minute 12:30; each photo is taken every 30 s. [file peerj-05-2810-s002.zip › PeerJ1/160720-039-Exp_Cryoelettro.jpg]

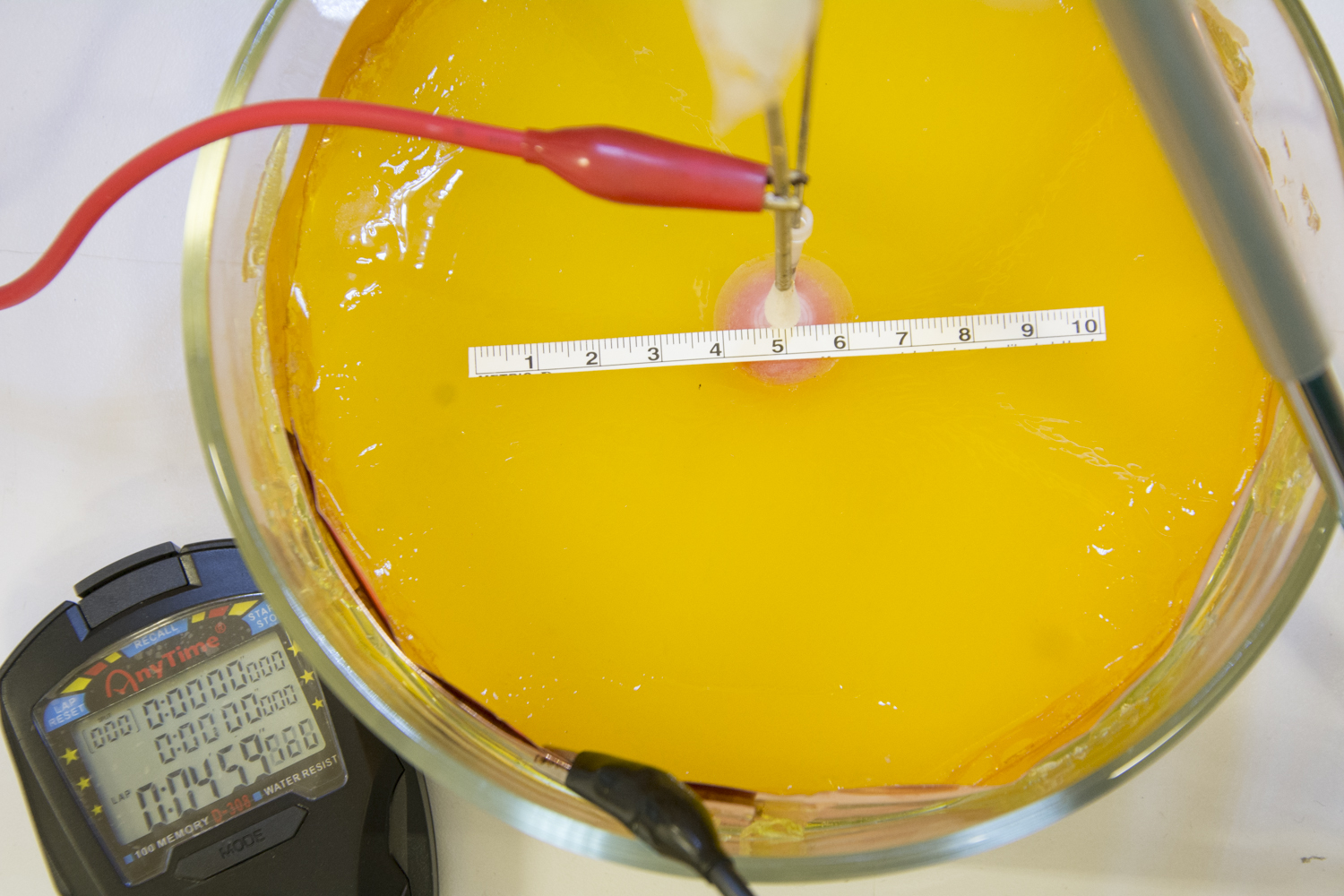

Supplement: Supplemental Information 2 — In particular: 160720-015-Exp_Cryoelettro shows details of the experiment, with camera, suppliers, probe and saline agar solution; 160720-016-Exp_Cryoelettro is a closer view of the setup. Photos from 160720-030-Exp_Cryoelettro to 160720-055-Exp_Cryoelettro shows the progression mentioned before from minute 0:00 to minute 12:30; each photo is taken every 30 s. [file peerj-05-2810-s002.zip › PeerJ1/160720-040-Exp_Cryoelettro.jpg]

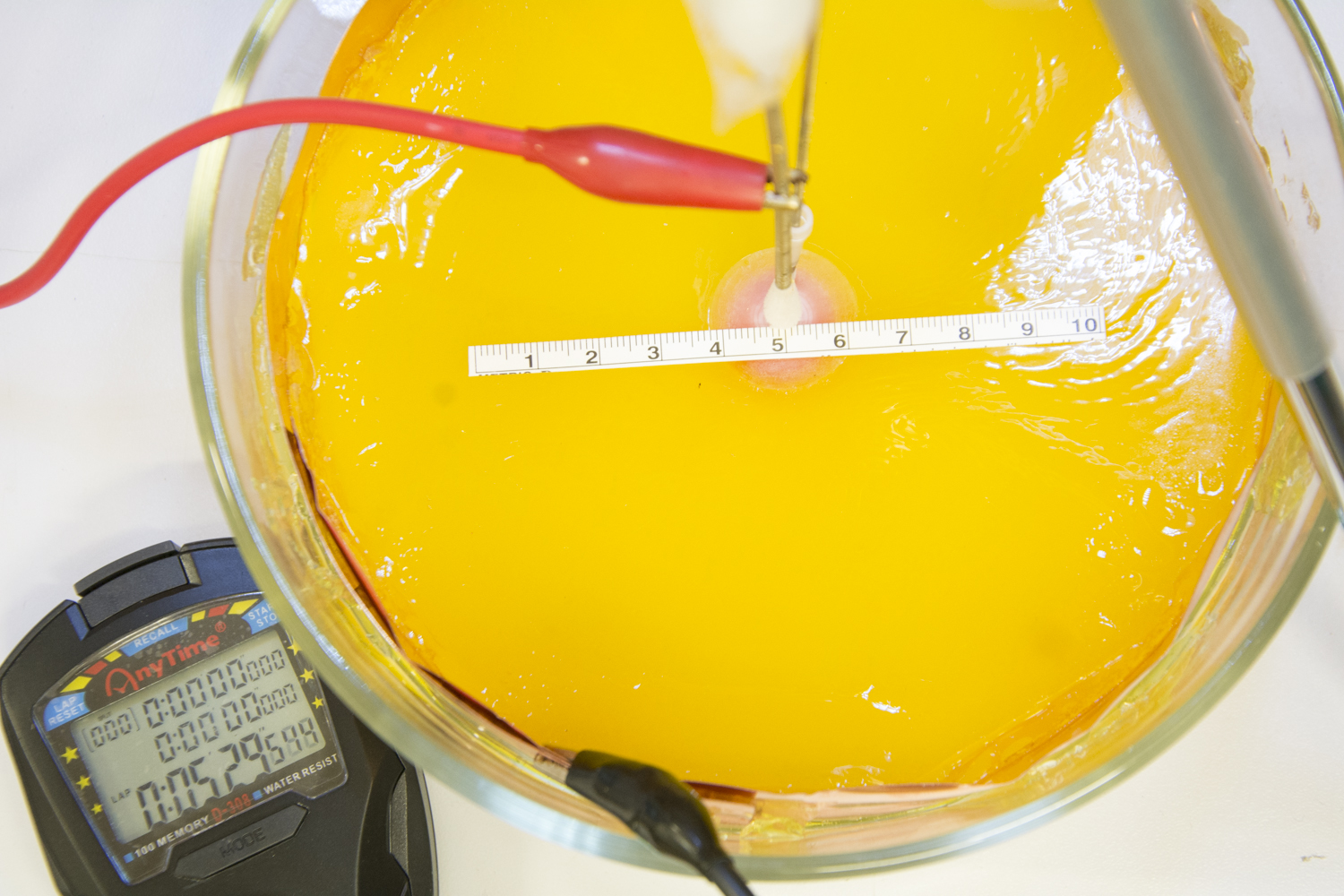

Supplement: Supplemental Information 2 — In particular: 160720-015-Exp_Cryoelettro shows details of the experiment, with camera, suppliers, probe and saline agar solution; 160720-016-Exp_Cryoelettro is a closer view of the setup. Photos from 160720-030-Exp_Cryoelettro to 160720-055-Exp_Cryoelettro shows the progression mentioned before from minute 0:00 to minute 12:30; each photo is taken every 30 s. [file peerj-05-2810-s002.zip › PeerJ1/160720-041-Exp_Cryoelettro.jpg]

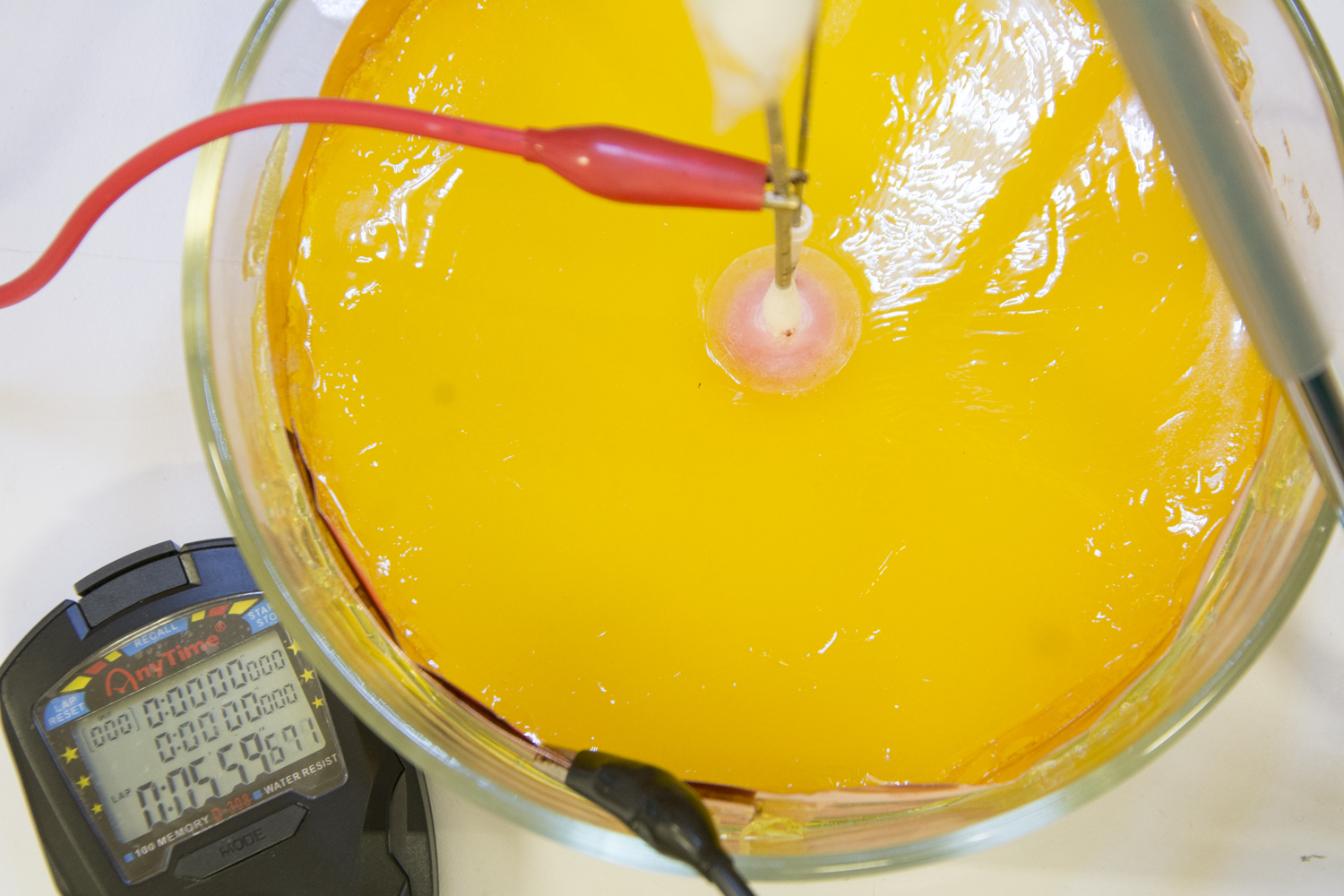

Supplement: Supplemental Information 2 — In particular: 160720-015-Exp_Cryoelettro shows details of the experiment, with camera, suppliers, probe and saline agar solution; 160720-016-Exp_Cryoelettro is a closer view of the setup. Photos from 160720-030-Exp_Cryoelettro to 160720-055-Exp_Cryoelettro shows the progression mentioned before from minute 0:00 to minute 12:30; each photo is taken every 30 s. [file peerj-05-2810-s002.zip › PeerJ1/160720-042-Exp_Cryoelettro.jpg]

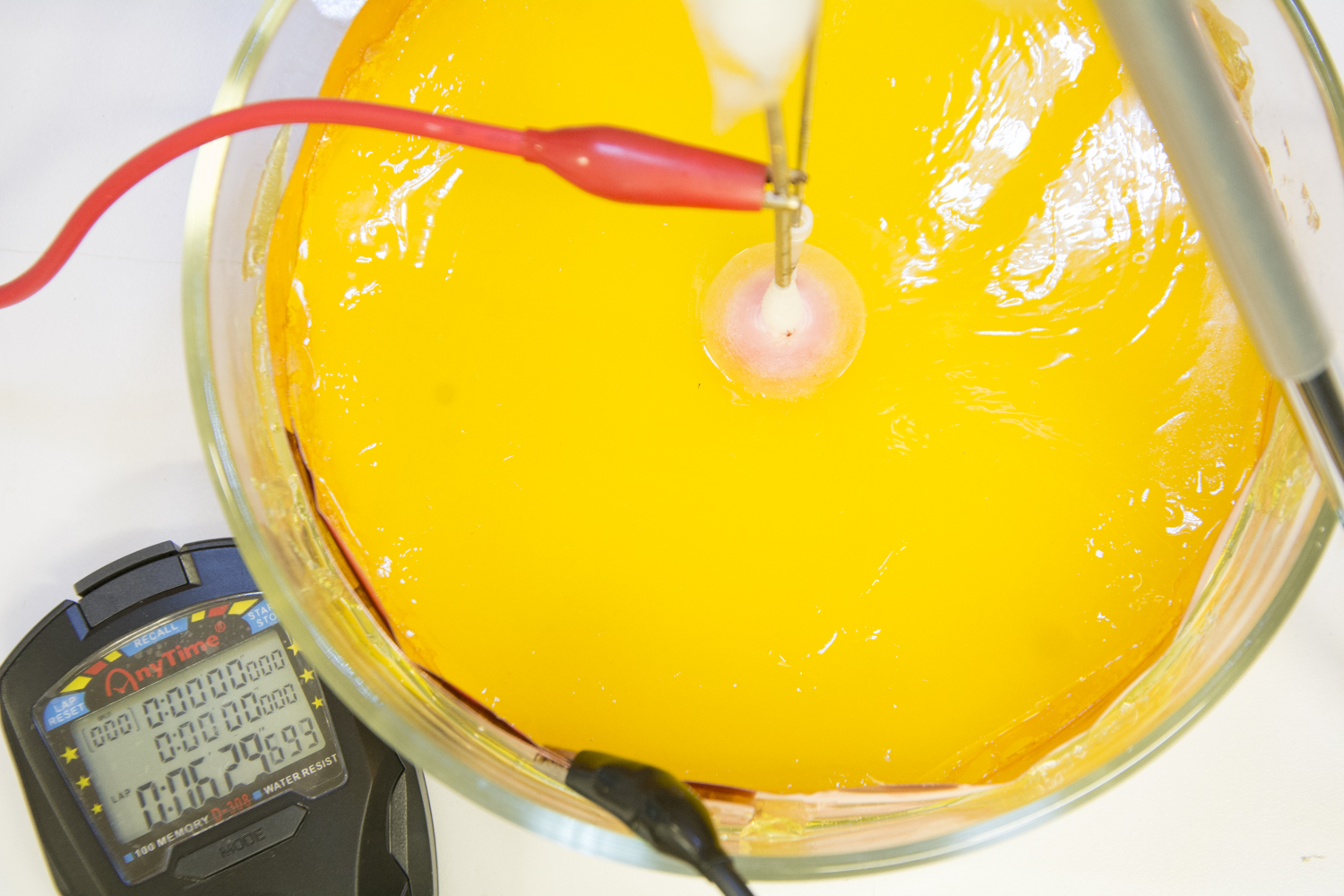

Supplement: Supplemental Information 2 — In particular: 160720-015-Exp_Cryoelettro shows details of the experiment, with camera, suppliers, probe and saline agar solution; 160720-016-Exp_Cryoelettro is a closer view of the setup. Photos from 160720-030-Exp_Cryoelettro to 160720-055-Exp_Cryoelettro shows the progression mentioned before from minute 0:00 to minute 12:30; each photo is taken every 30 s. [file peerj-05-2810-s002.zip › PeerJ1/160720-043-Exp_Cryoelettro.jpg]

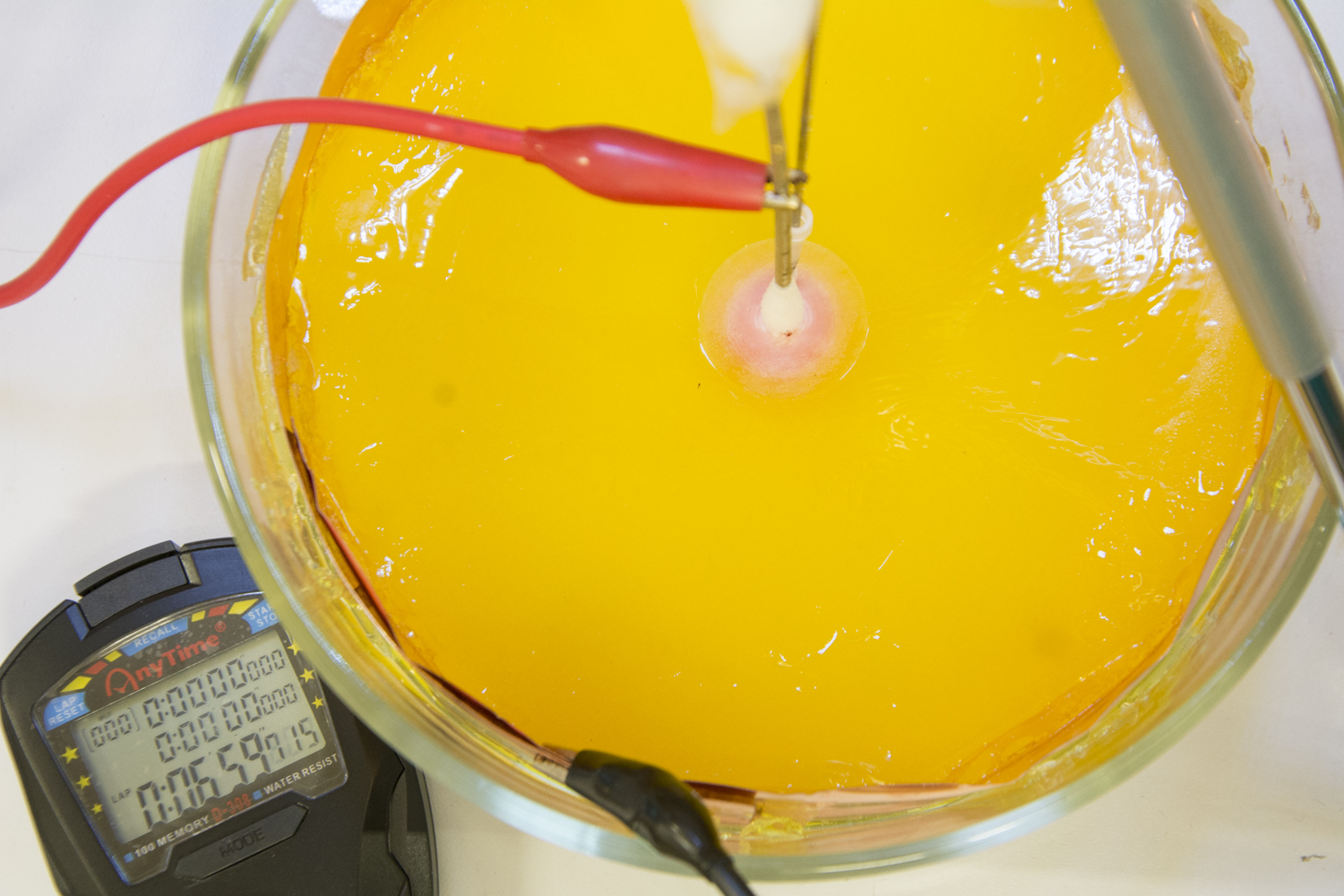

Supplement: Supplemental Information 2 — In particular: 160720-015-Exp_Cryoelettro shows details of the experiment, with camera, suppliers, probe and saline agar solution; 160720-016-Exp_Cryoelettro is a closer view of the setup. Photos from 160720-030-Exp_Cryoelettro to 160720-055-Exp_Cryoelettro shows the progression mentioned before from minute 0:00 to minute 12:30; each photo is taken every 30 s. [file peerj-05-2810-s002.zip › PeerJ1/160720-044-Exp_Cryoelettro.jpg]

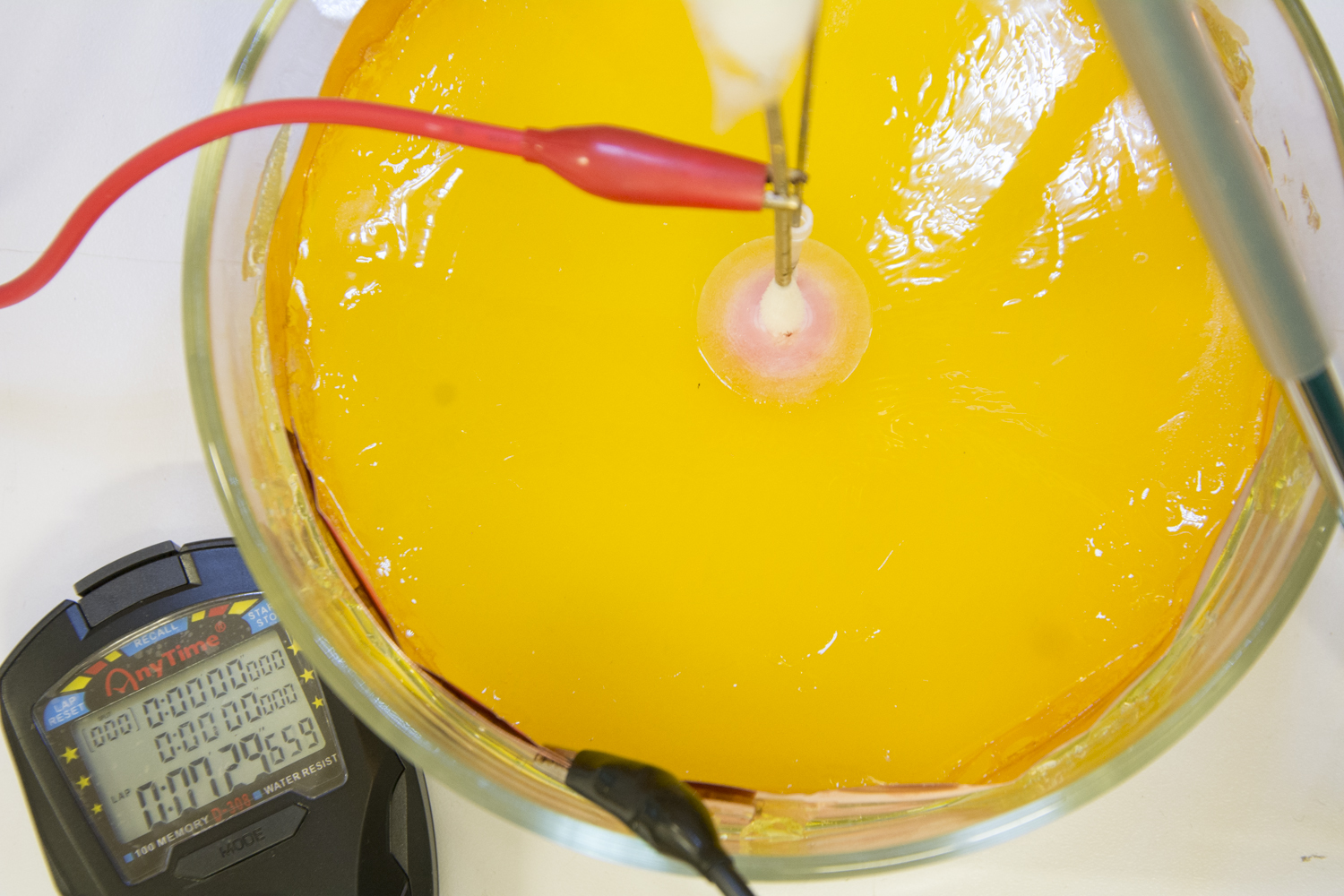

Supplement: Supplemental Information 2 — In particular: 160720-015-Exp_Cryoelettro shows details of the experiment, with camera, suppliers, probe and saline agar solution; 160720-016-Exp_Cryoelettro is a closer view of the setup. Photos from 160720-030-Exp_Cryoelettro to 160720-055-Exp_Cryoelettro shows the progression mentioned before from minute 0:00 to minute 12:30; each photo is taken every 30 s. [file peerj-05-2810-s002.zip › PeerJ1/160720-045-Exp_Cryoelettro.jpg]

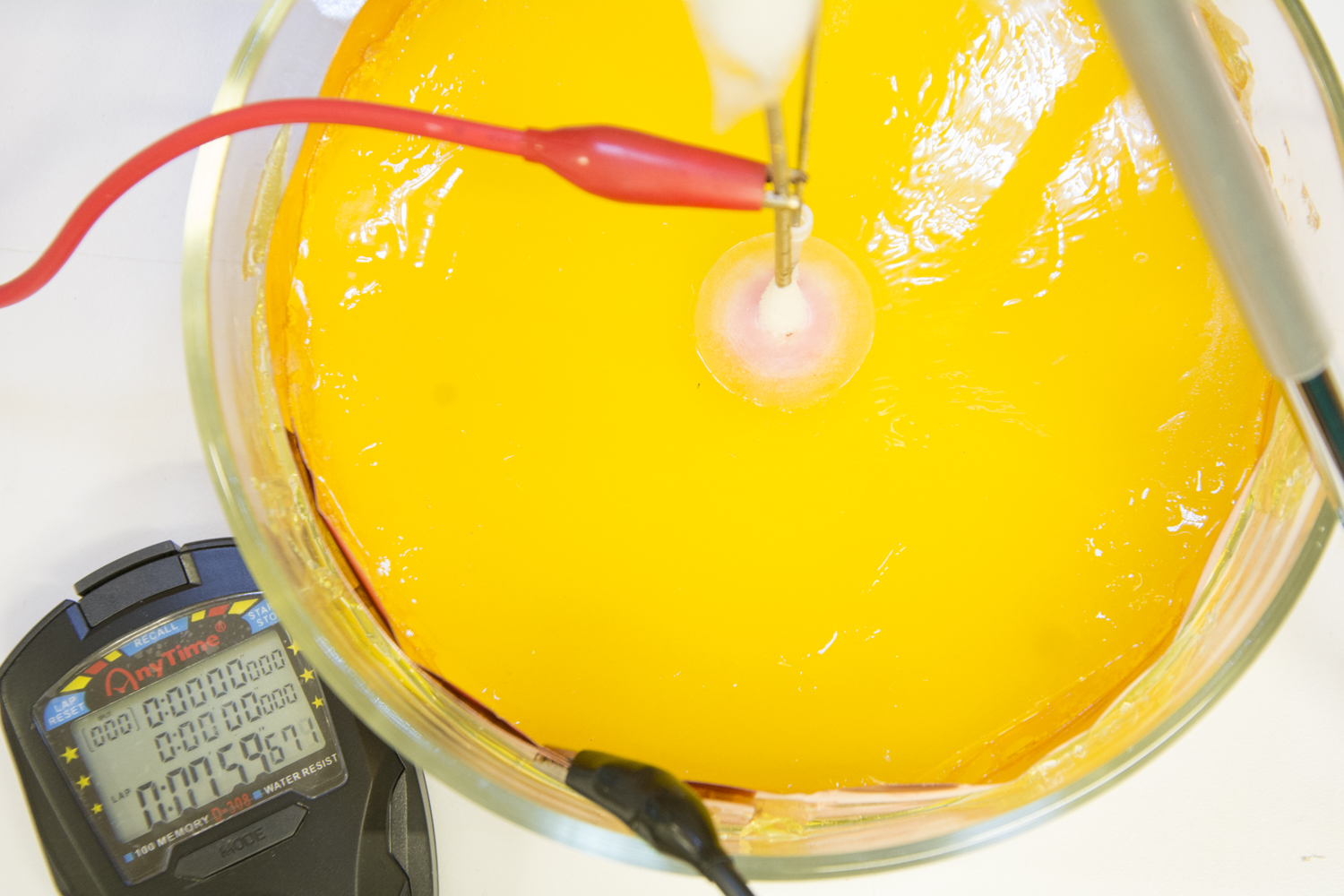

Supplement: Supplemental Information 2 — In particular: 160720-015-Exp_Cryoelettro shows details of the experiment, with camera, suppliers, probe and saline agar solution; 160720-016-Exp_Cryoelettro is a closer view of the setup. Photos from 160720-030-Exp_Cryoelettro to 160720-055-Exp_Cryoelettro shows the progression mentioned before from minute 0:00 to minute 12:30; each photo is taken every 30 s. [file peerj-05-2810-s002.zip › PeerJ1/160720-046-Exp_Cryoelettro.jpg]

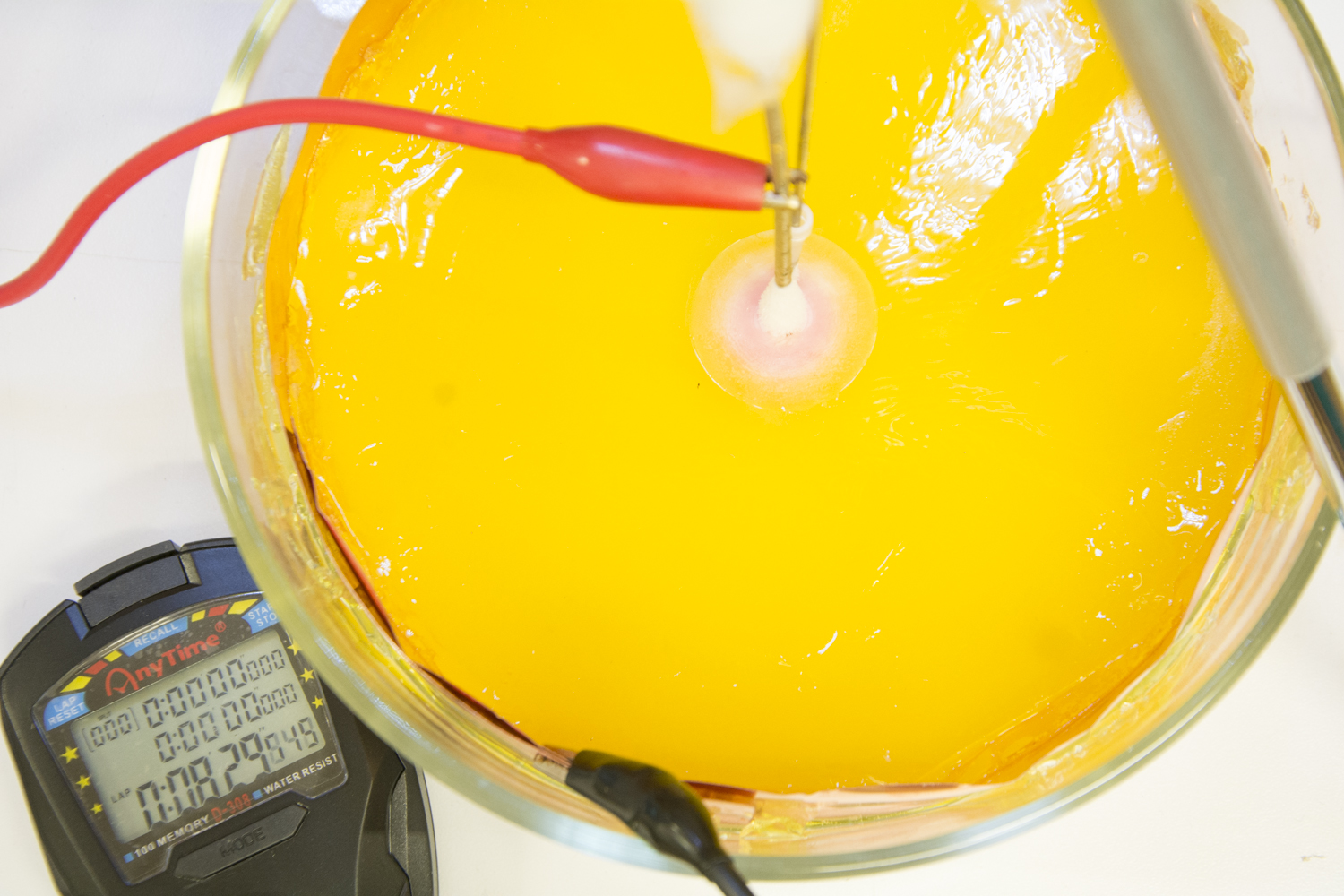

Supplement: Supplemental Information 2 — In particular: 160720-015-Exp_Cryoelettro shows details of the experiment, with camera, suppliers, probe and saline agar solution; 160720-016-Exp_Cryoelettro is a closer view of the setup. Photos from 160720-030-Exp_Cryoelettro to 160720-055-Exp_Cryoelettro shows the progression mentioned before from minute 0:00 to minute 12:30; each photo is taken every 30 s. [file peerj-05-2810-s002.zip › PeerJ1/160720-047-Exp_Cryoelettro.jpg]

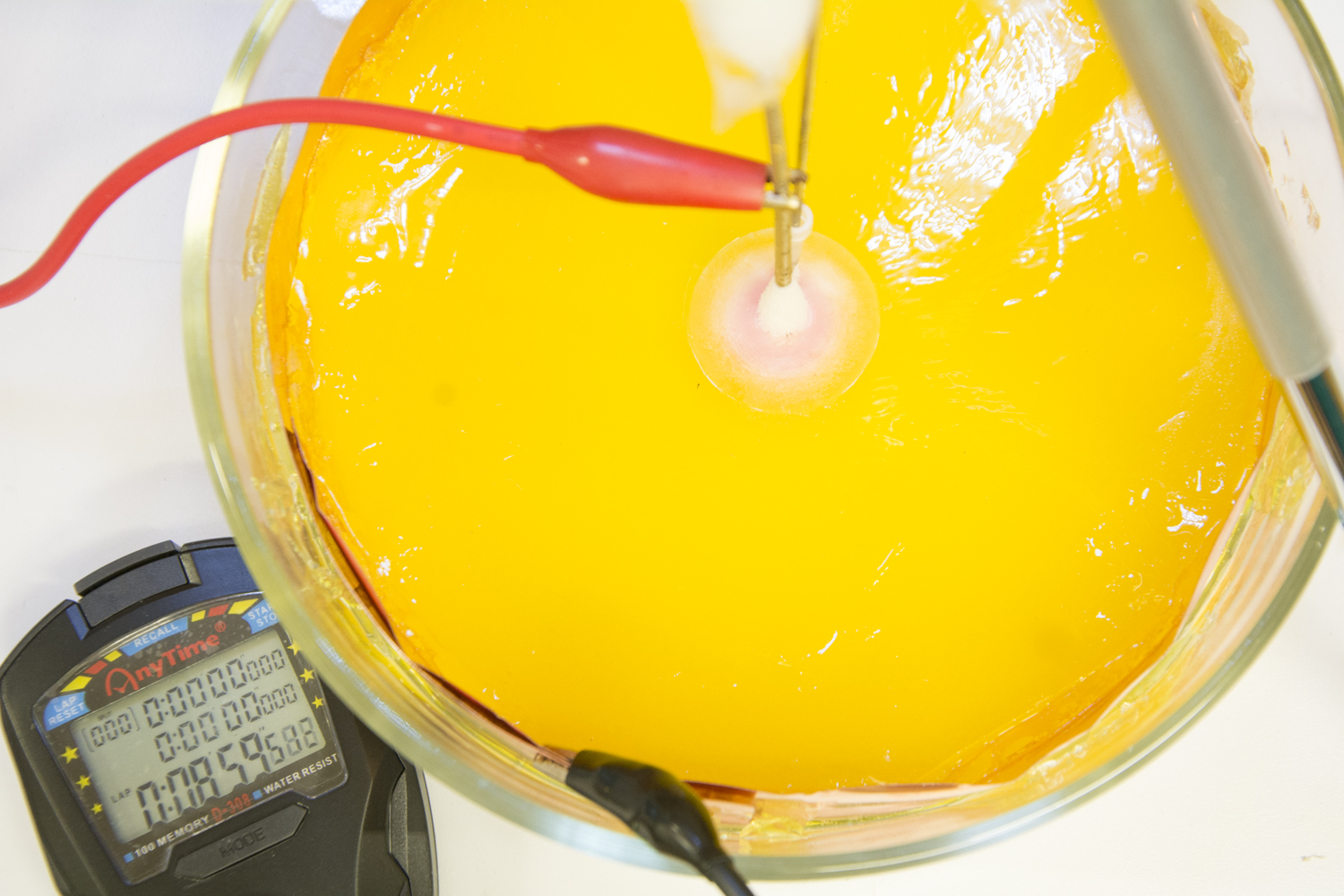

Supplement: Supplemental Information 2 — In particular: 160720-015-Exp_Cryoelettro shows details of the experiment, with camera, suppliers, probe and saline agar solution; 160720-016-Exp_Cryoelettro is a closer view of the setup. Photos from 160720-030-Exp_Cryoelettro to 160720-055-Exp_Cryoelettro shows the progression mentioned before from minute 0:00 to minute 12:30; each photo is taken every 30 s. [file peerj-05-2810-s002.zip › PeerJ1/160720-048-Exp_Cryoelettro.jpg]

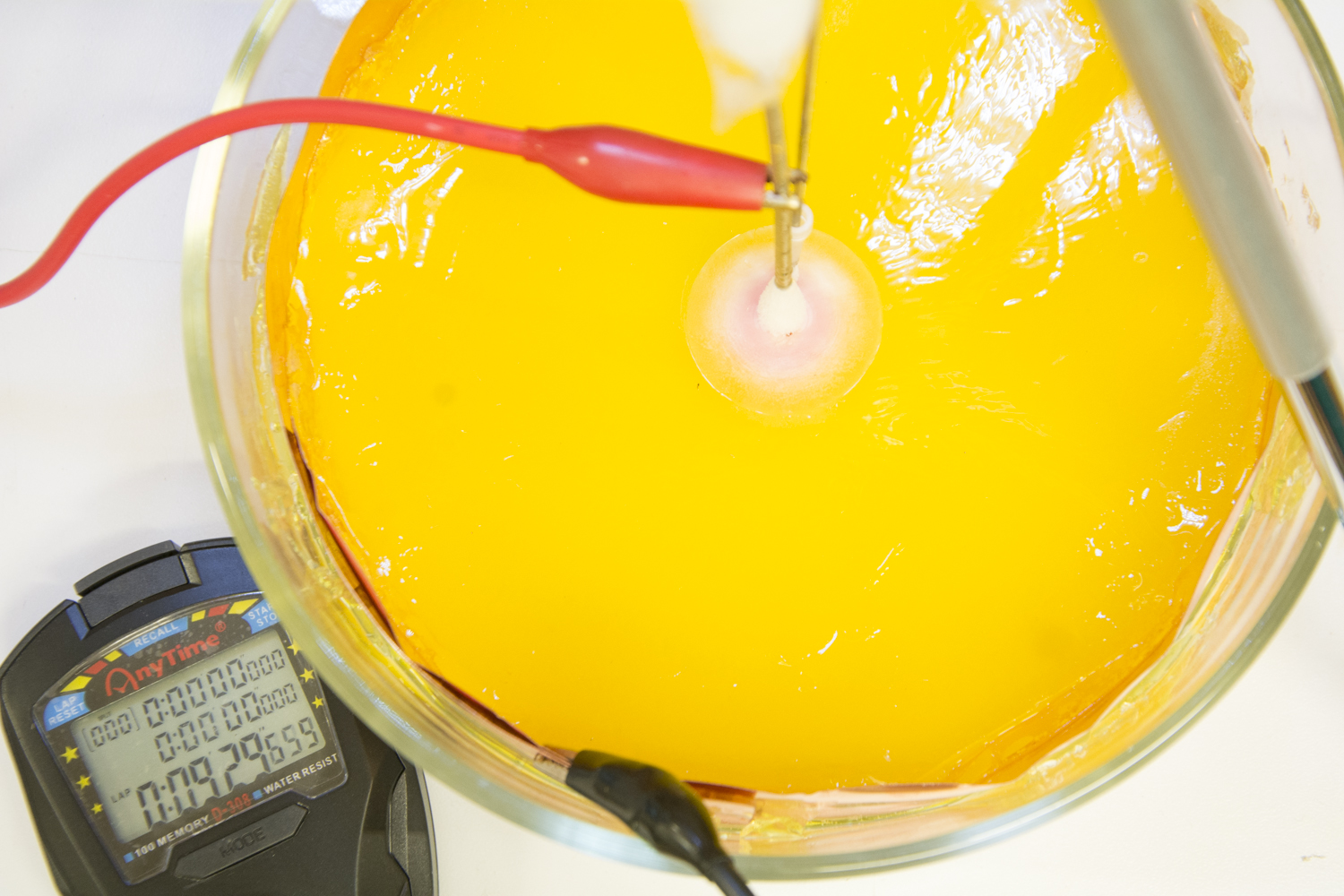

Supplement: Supplemental Information 2 — In particular: 160720-015-Exp_Cryoelettro shows details of the experiment, with camera, suppliers, probe and saline agar solution; 160720-016-Exp_Cryoelettro is a closer view of the setup. Photos from 160720-030-Exp_Cryoelettro to 160720-055-Exp_Cryoelettro shows the progression mentioned before from minute 0:00 to minute 12:30; each photo is taken every 30 s. [file peerj-05-2810-s002.zip › PeerJ1/160720-049-Exp_Cryoelettro.jpg]

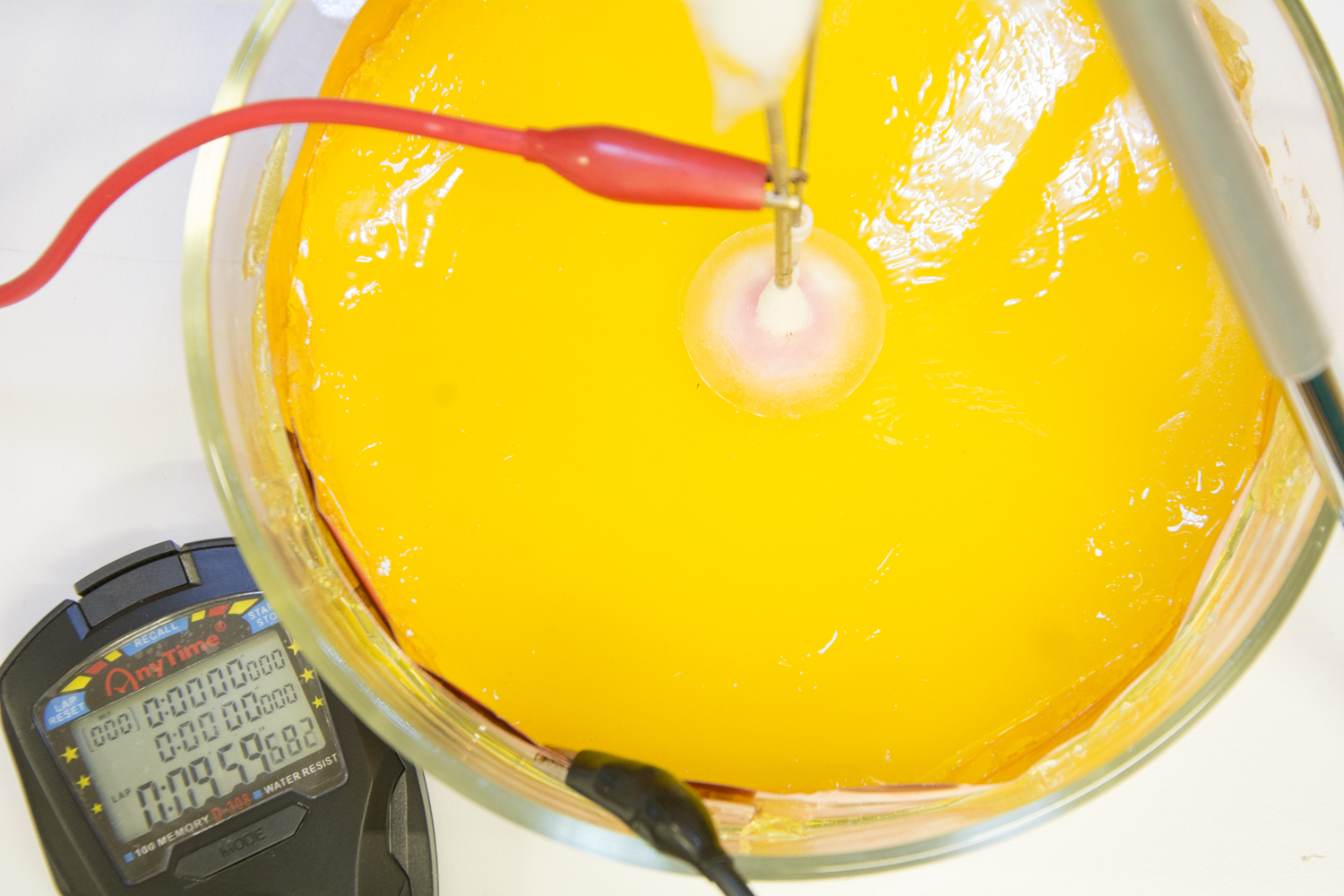

Supplement: Supplemental Information 2 — In particular: 160720-015-Exp_Cryoelettro shows details of the experiment, with camera, suppliers, probe and saline agar solution; 160720-016-Exp_Cryoelettro is a closer view of the setup. Photos from 160720-030-Exp_Cryoelettro to 160720-055-Exp_Cryoelettro shows the progression mentioned before from minute 0:00 to minute 12:30; each photo is taken every 30 s. [file peerj-05-2810-s002.zip › PeerJ1/160720-050-Exp_Cryoelettro.jpg]

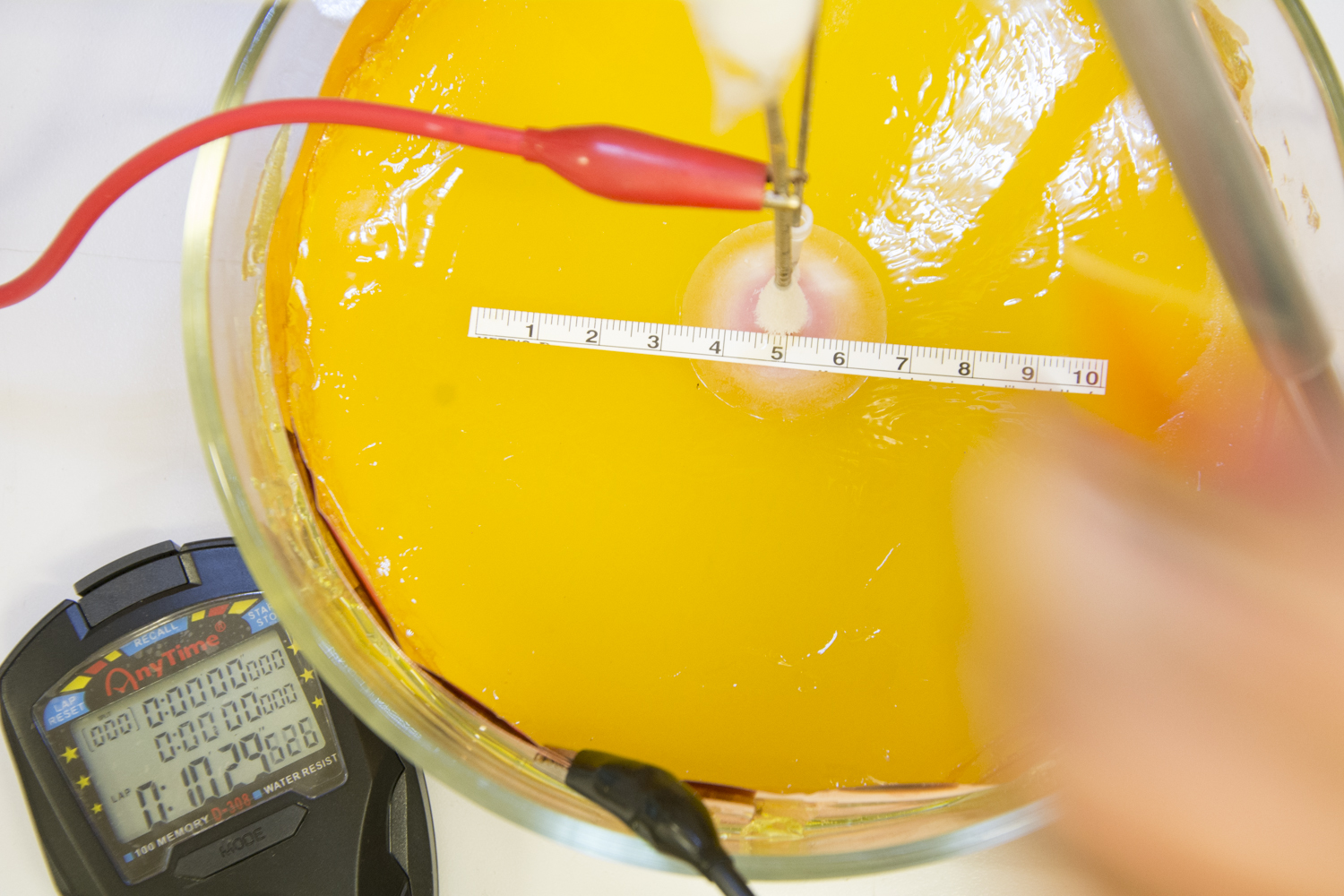

Supplement: Supplemental Information 2 — In particular: 160720-015-Exp_Cryoelettro shows details of the experiment, with camera, suppliers, probe and saline agar solution; 160720-016-Exp_Cryoelettro is a closer view of the setup. Photos from 160720-030-Exp_Cryoelettro to 160720-055-Exp_Cryoelettro shows the progression mentioned before from minute 0:00 to minute 12:30; each photo is taken every 30 s. [file peerj-05-2810-s002.zip › PeerJ1/160720-051-Exp_Cryoelettro.jpg]

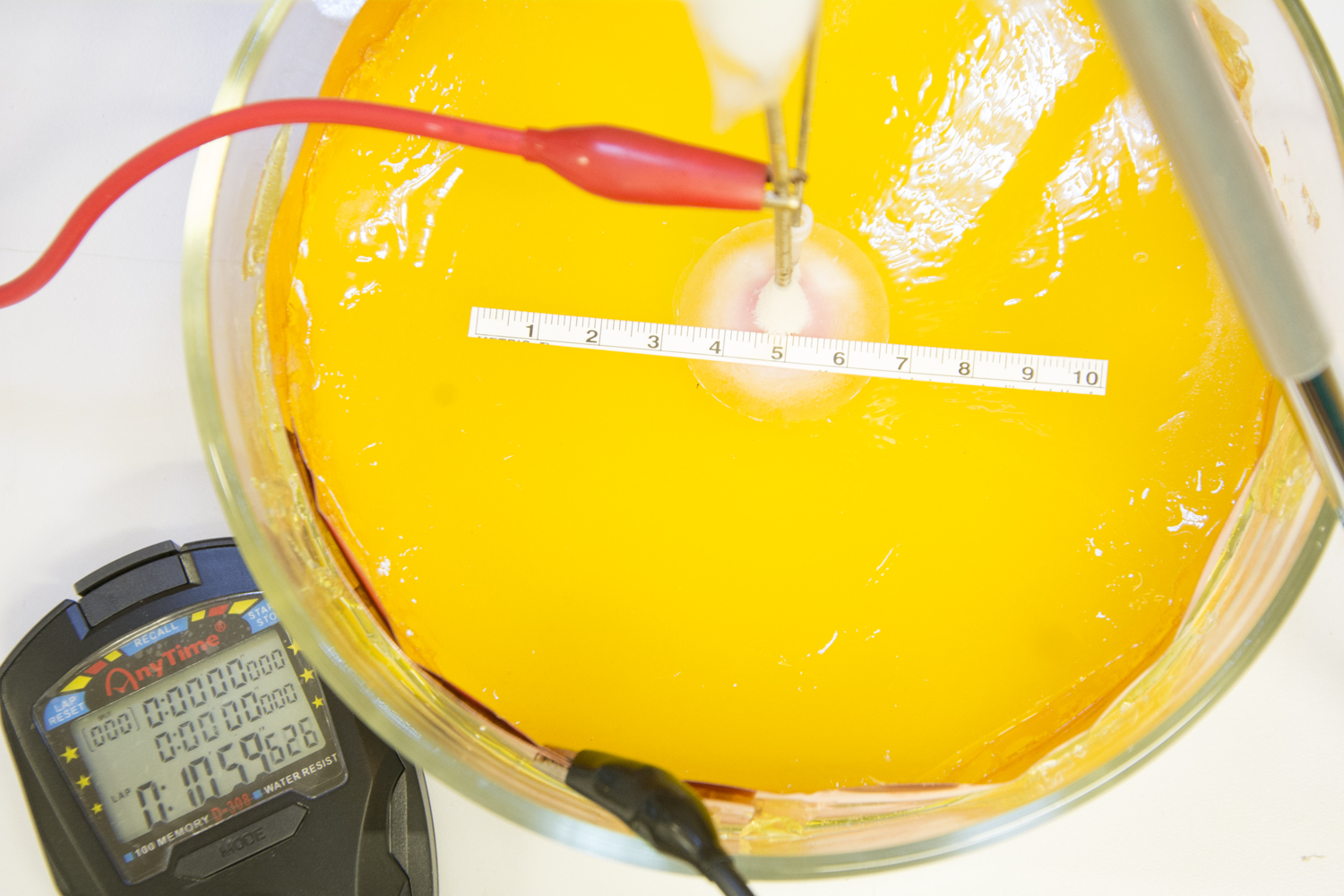

Supplement: Supplemental Information 2 — In particular: 160720-015-Exp_Cryoelettro shows details of the experiment, with camera, suppliers, probe and saline agar solution; 160720-016-Exp_Cryoelettro is a closer view of the setup. Photos from 160720-030-Exp_Cryoelettro to 160720-055-Exp_Cryoelettro shows the progression mentioned before from minute 0:00 to minute 12:30; each photo is taken every 30 s. [file peerj-05-2810-s002.zip › PeerJ1/160720-052-Exp_Cryoelettro.jpg]

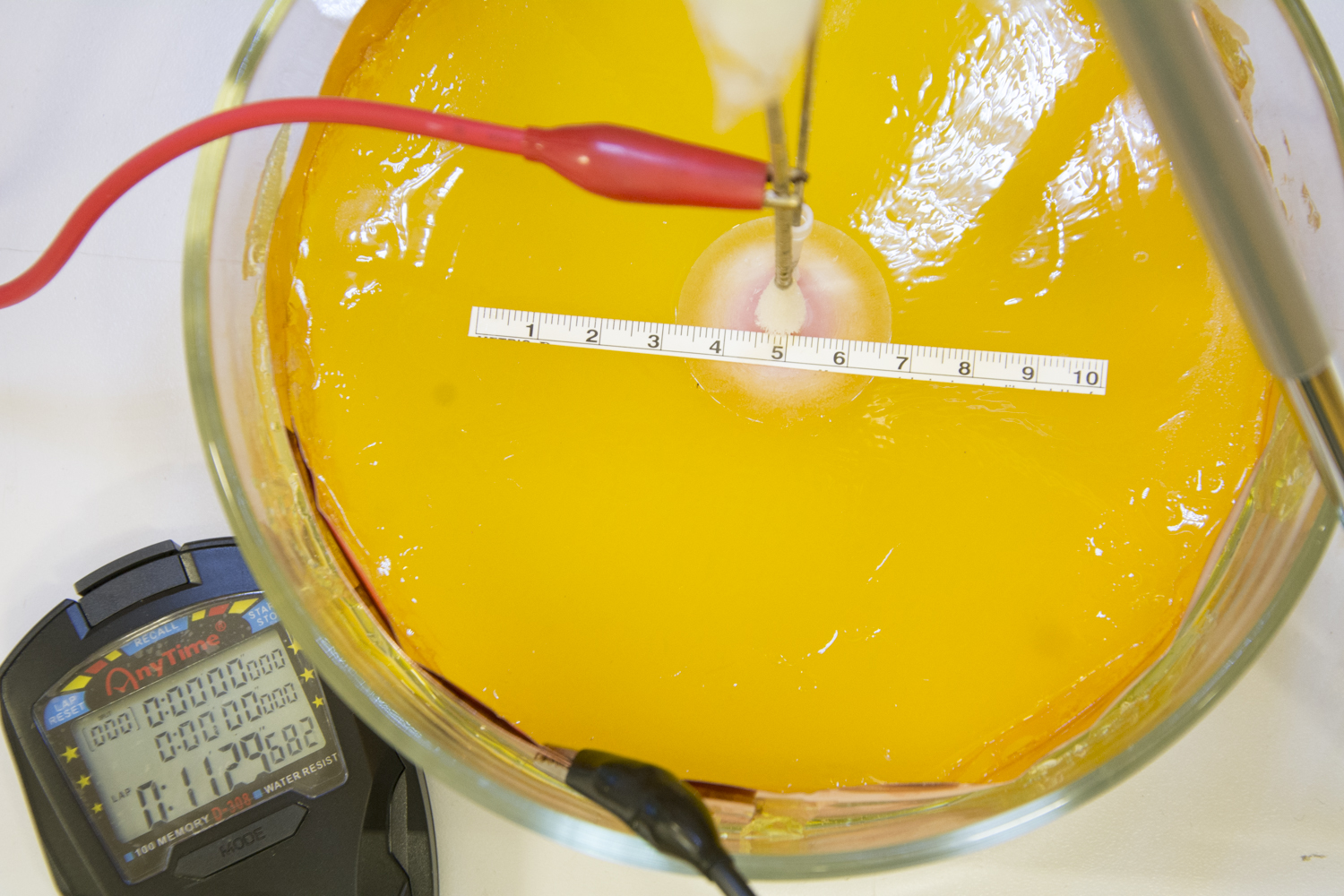

Supplement: Supplemental Information 2 — In particular: 160720-015-Exp_Cryoelettro shows details of the experiment, with camera, suppliers, probe and saline agar solution; 160720-016-Exp_Cryoelettro is a closer view of the setup. Photos from 160720-030-Exp_Cryoelettro to 160720-055-Exp_Cryoelettro shows the progression mentioned before from minute 0:00 to minute 12:30; each photo is taken every 30 s. [file peerj-05-2810-s002.zip › PeerJ1/160720-053-Exp_Cryoelettro.jpg]

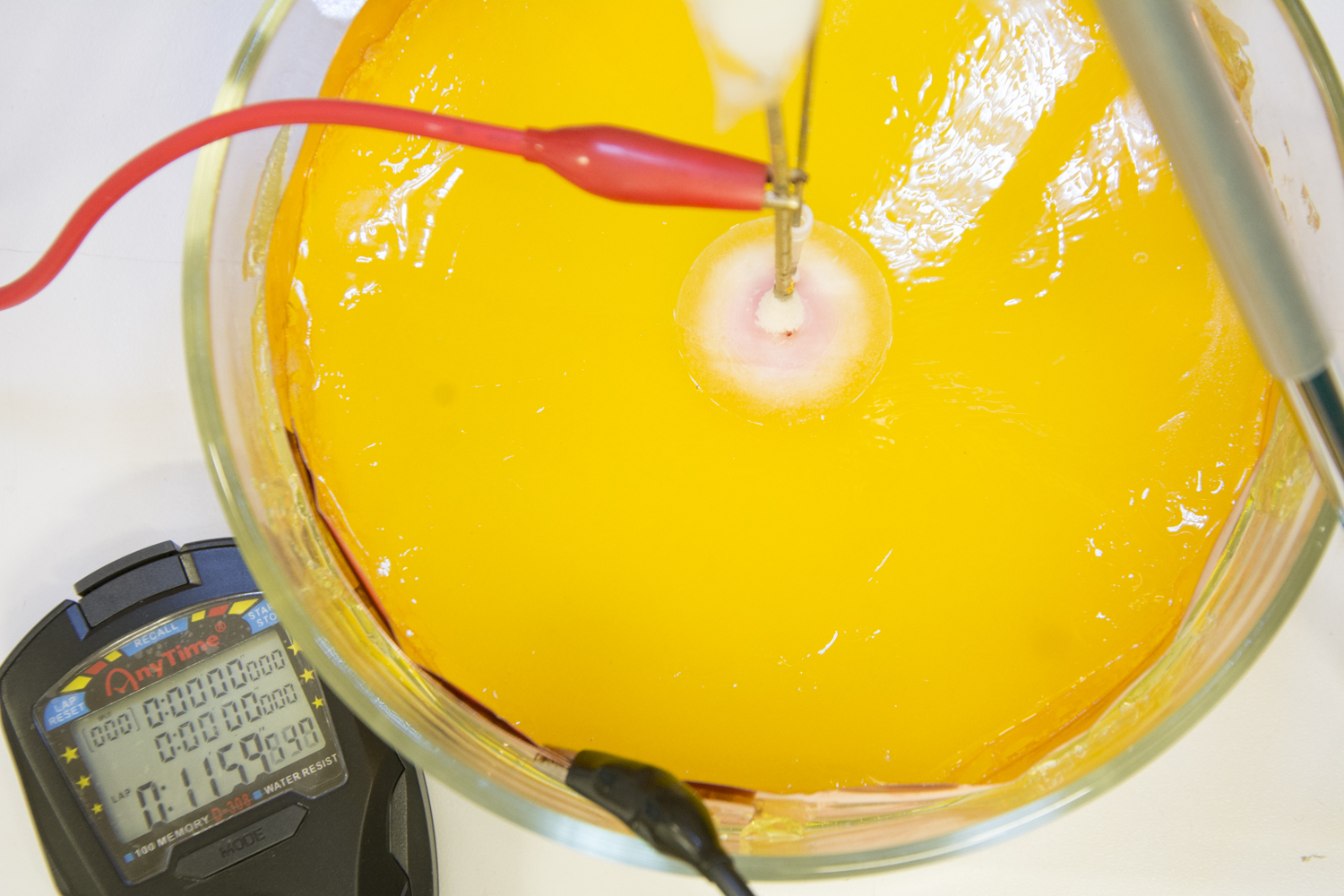

Supplement: Supplemental Information 2 — In particular: 160720-015-Exp_Cryoelettro shows details of the experiment, with camera, suppliers, probe and saline agar solution; 160720-016-Exp_Cryoelettro is a closer view of the setup. Photos from 160720-030-Exp_Cryoelettro to 160720-055-Exp_Cryoelettro shows the progression mentioned before from minute 0:00 to minute 12:30; each photo is taken every 30 s. [file peerj-05-2810-s002.zip › PeerJ1/160720-054-Exp_Cryoelettro.jpg]

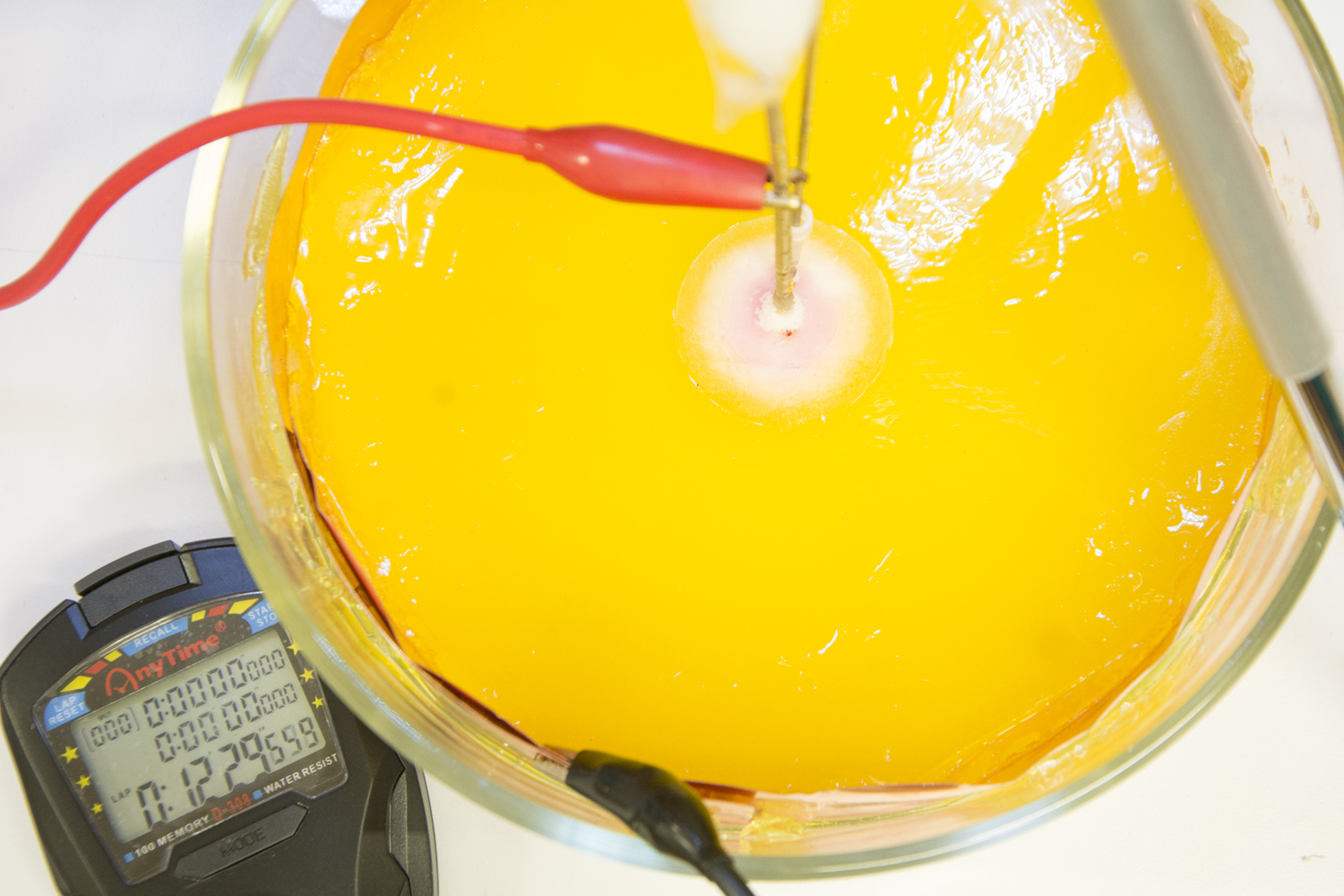

Supplement: Supplemental Information 2 — In particular: 160720-015-Exp_Cryoelettro shows details of the experiment, with camera, suppliers, probe and saline agar solution; 160720-016-Exp_Cryoelettro is a closer view of the setup. Photos from 160720-030-Exp_Cryoelettro to 160720-055-Exp_Cryoelettro shows the progression mentioned before from minute 0:00 to minute 12:30; each photo is taken every 30 s. [file peerj-05-2810-s002.zip › PeerJ1/160720-055-Exp_Cryoelettro.jpg]

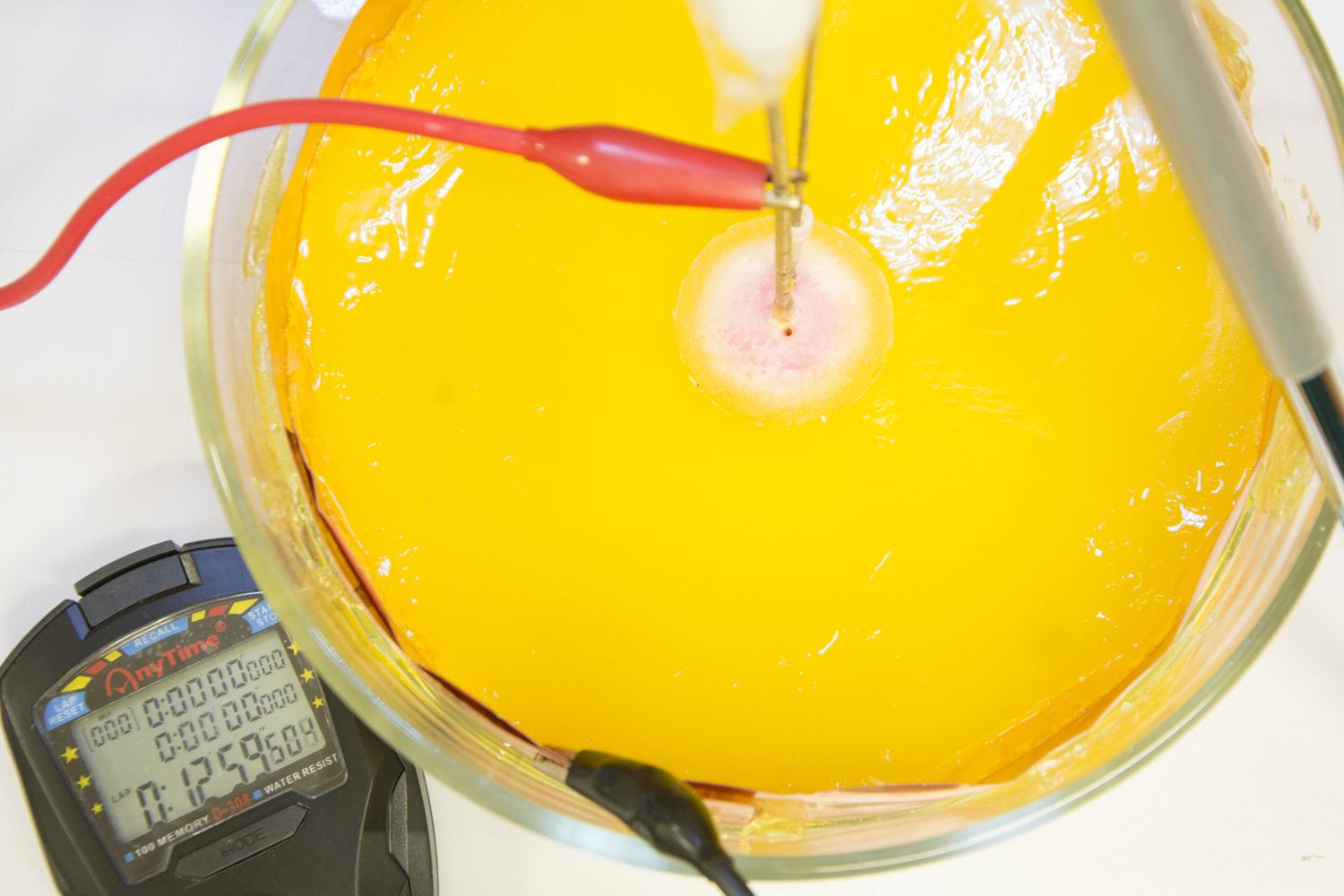

Supplement: Supplemental Information 3 — Photos from 160720-056-Exp_Cryoelettro to 160720-082-Exp_Cryoelettro shows the progression mentioned before from minute 13:00 to minute 26:00; each photo is taken every 30 s. [file peerj-05-2810-s003.zip › PeerJ2/160720-056-Exp_Cryoelettro.jpg]

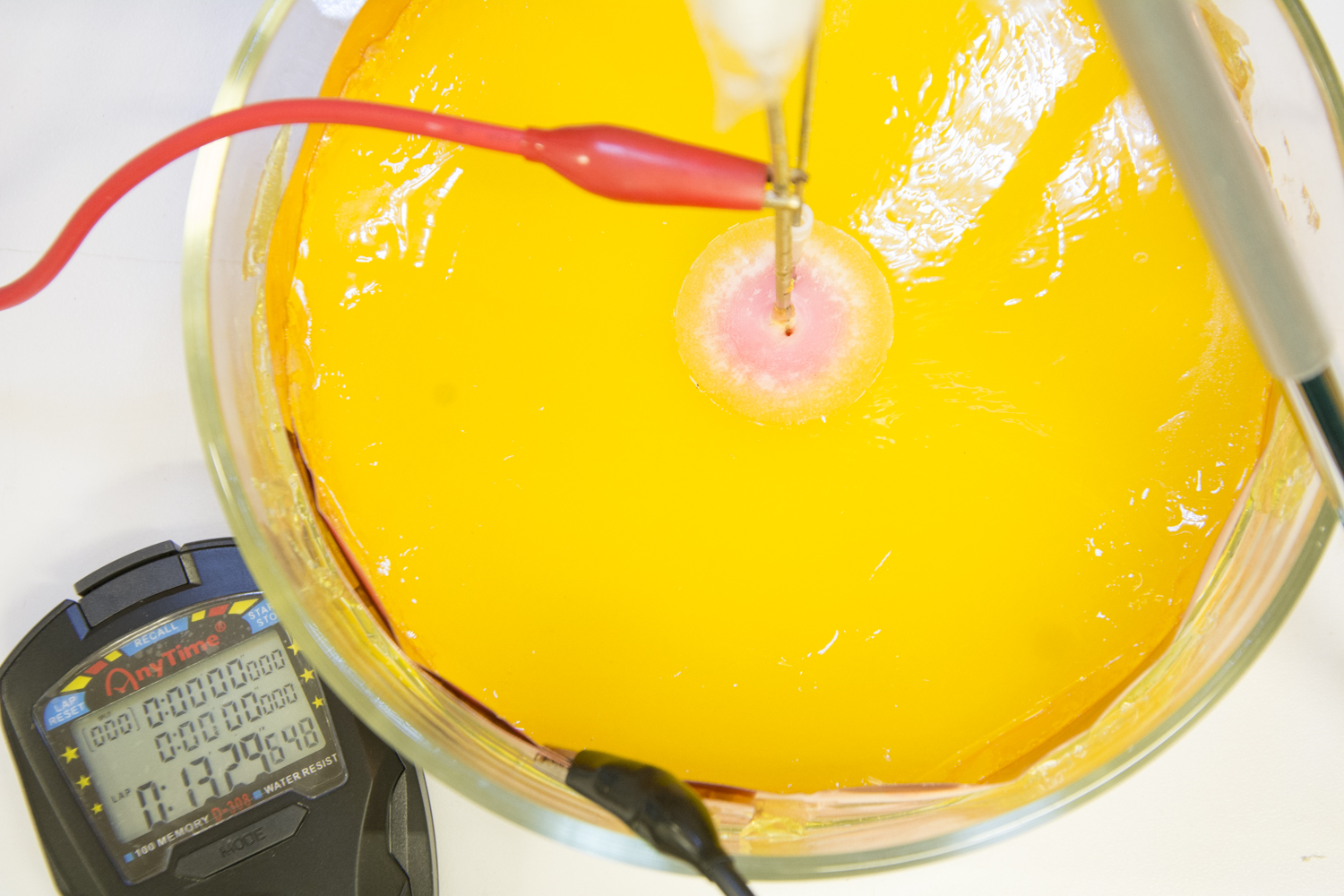

Supplement: Supplemental Information 3 — Photos from 160720-056-Exp_Cryoelettro to 160720-082-Exp_Cryoelettro shows the progression mentioned before from minute 13:00 to minute 26:00; each photo is taken every 30 s. [file peerj-05-2810-s003.zip › PeerJ2/160720-057-Exp_Cryoelettro.jpg]

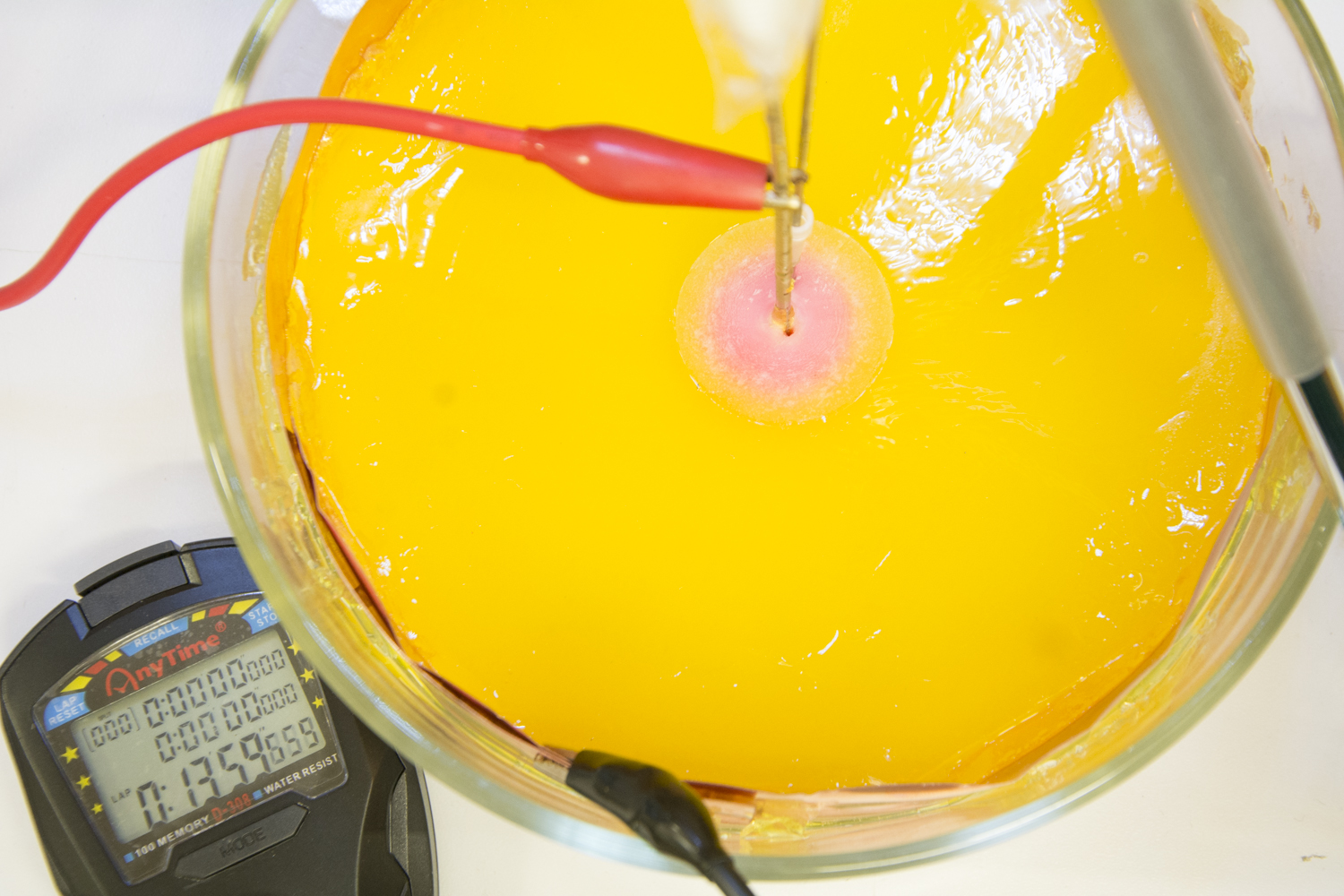

Supplement: Supplemental Information 3 — Photos from 160720-056-Exp_Cryoelettro to 160720-082-Exp_Cryoelettro shows the progression mentioned before from minute 13:00 to minute 26:00; each photo is taken every 30 s. [file peerj-05-2810-s003.zip › PeerJ2/160720-058-Exp_Cryoelettro.jpg]

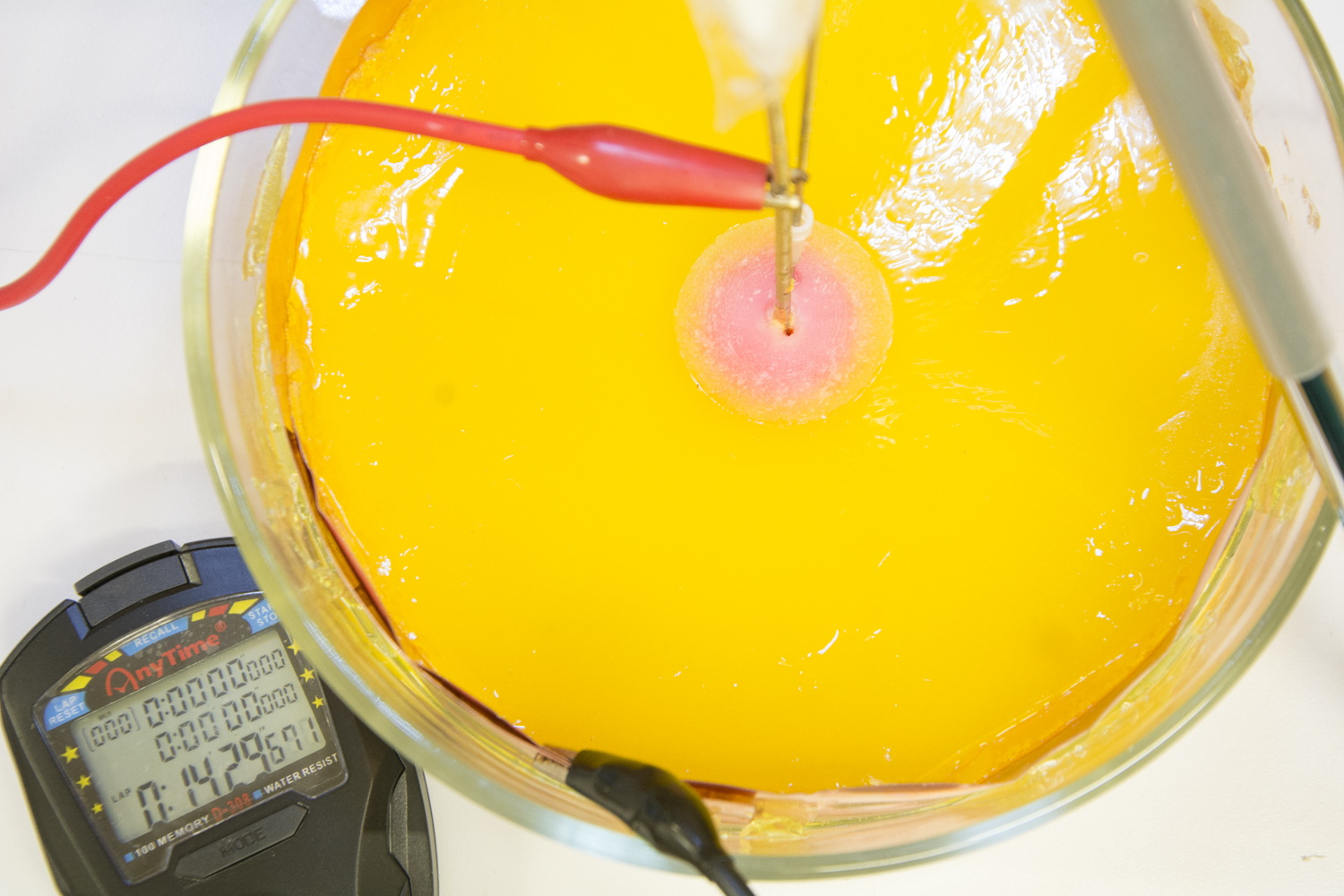

Supplement: Supplemental Information 3 — Photos from 160720-056-Exp_Cryoelettro to 160720-082-Exp_Cryoelettro shows the progression mentioned before from minute 13:00 to minute 26:00; each photo is taken every 30 s. [file peerj-05-2810-s003.zip › PeerJ2/160720-059-Exp_Cryoelettro.jpg]

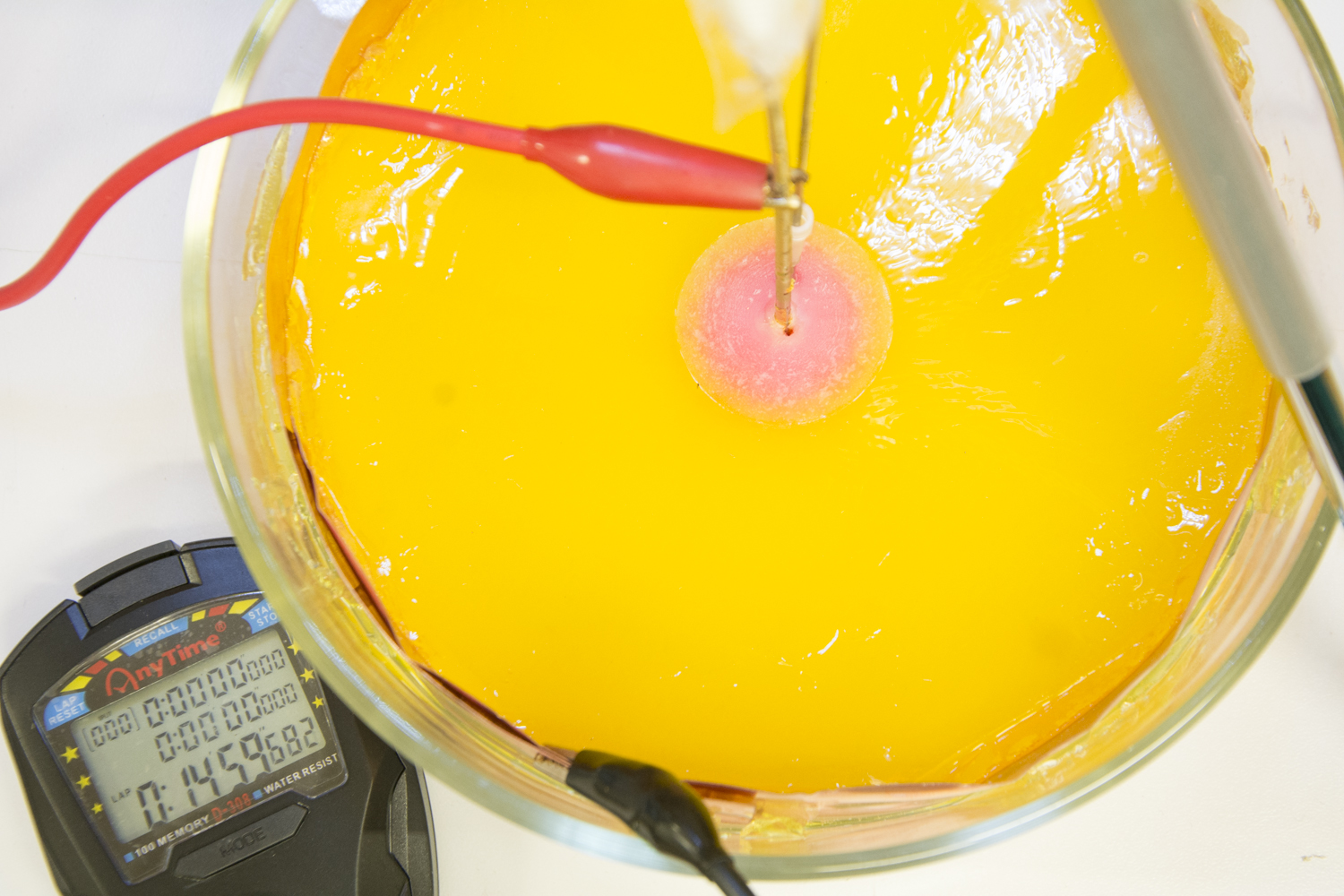

Supplement: Supplemental Information 3 — Photos from 160720-056-Exp_Cryoelettro to 160720-082-Exp_Cryoelettro shows the progression mentioned before from minute 13:00 to minute 26:00; each photo is taken every 30 s. [file peerj-05-2810-s003.zip › PeerJ2/160720-060-Exp_Cryoelettro.jpg]

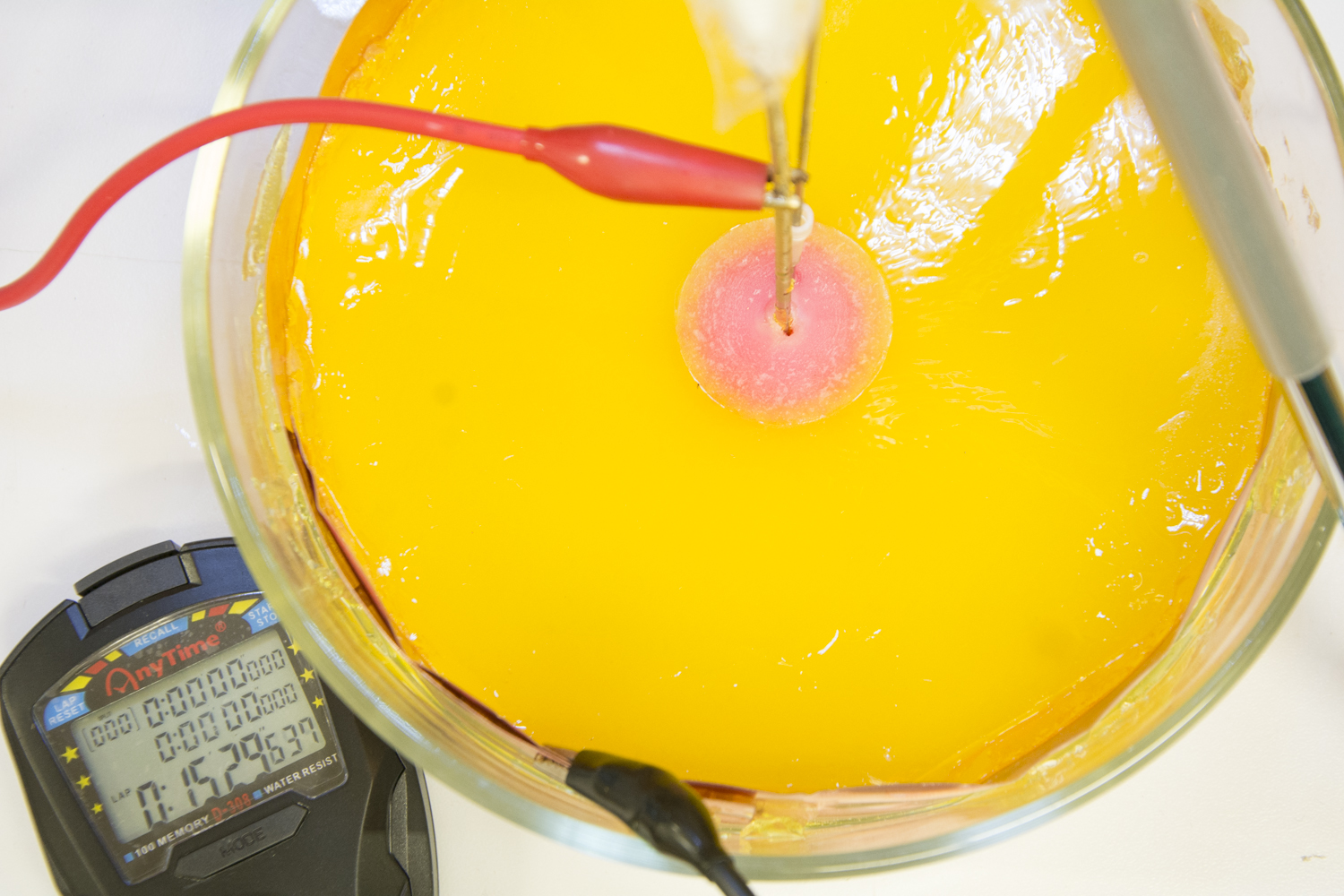

Supplement: Supplemental Information 3 — Photos from 160720-056-Exp_Cryoelettro to 160720-082-Exp_Cryoelettro shows the progression mentioned before from minute 13:00 to minute 26:00; each photo is taken every 30 s. [file peerj-05-2810-s003.zip › PeerJ2/160720-061-Exp_Cryoelettro.jpg]

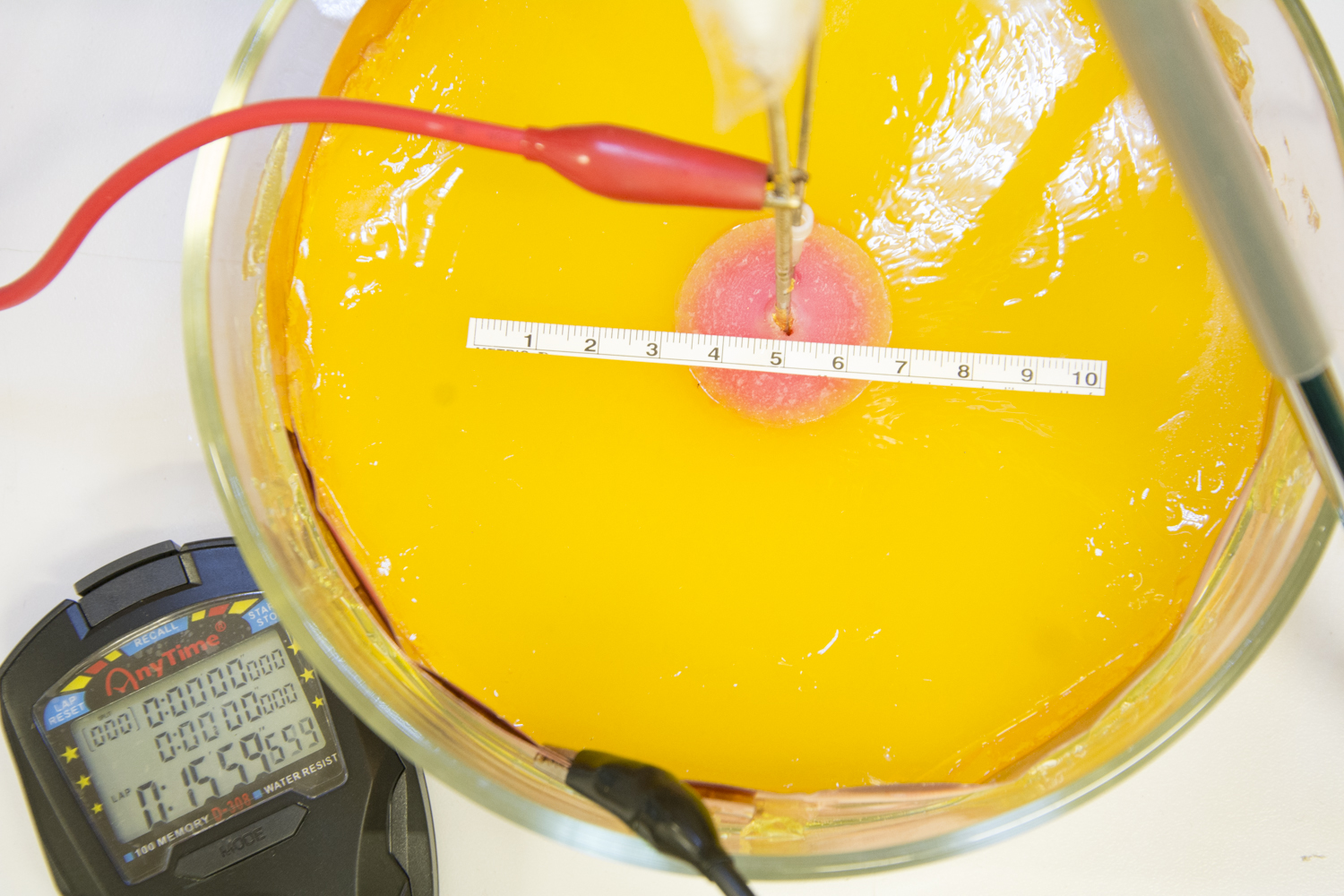

Supplement: Supplemental Information 3 — Photos from 160720-056-Exp_Cryoelettro to 160720-082-Exp_Cryoelettro shows the progression mentioned before from minute 13:00 to minute 26:00; each photo is taken every 30 s. [file peerj-05-2810-s003.zip › PeerJ2/160720-062-Exp_Cryoelettro.jpg]

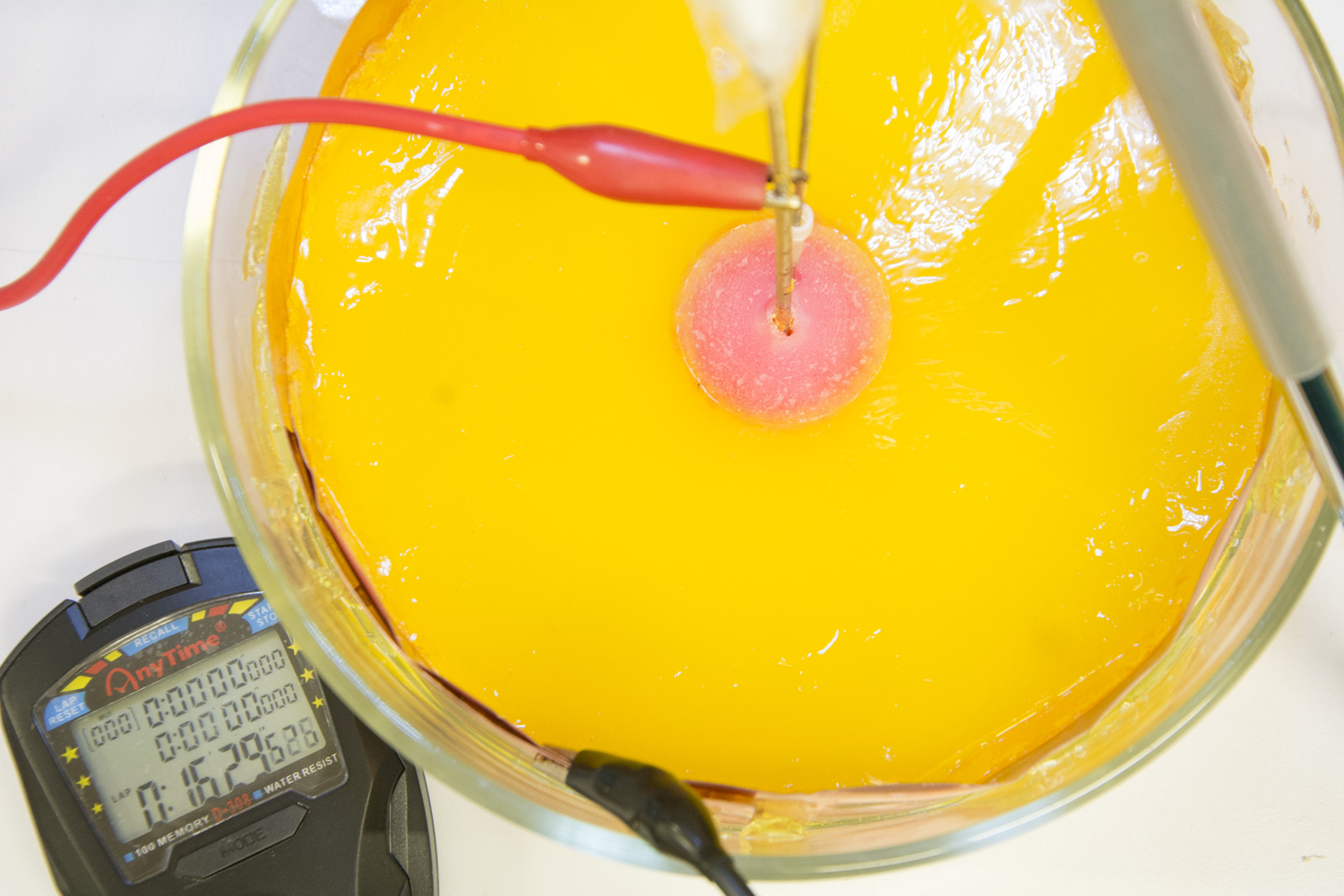

Supplement: Supplemental Information 3 — Photos from 160720-056-Exp_Cryoelettro to 160720-082-Exp_Cryoelettro shows the progression mentioned before from minute 13:00 to minute 26:00; each photo is taken every 30 s. [file peerj-05-2810-s003.zip › PeerJ2/160720-063-Exp_Cryoelettro.jpg]

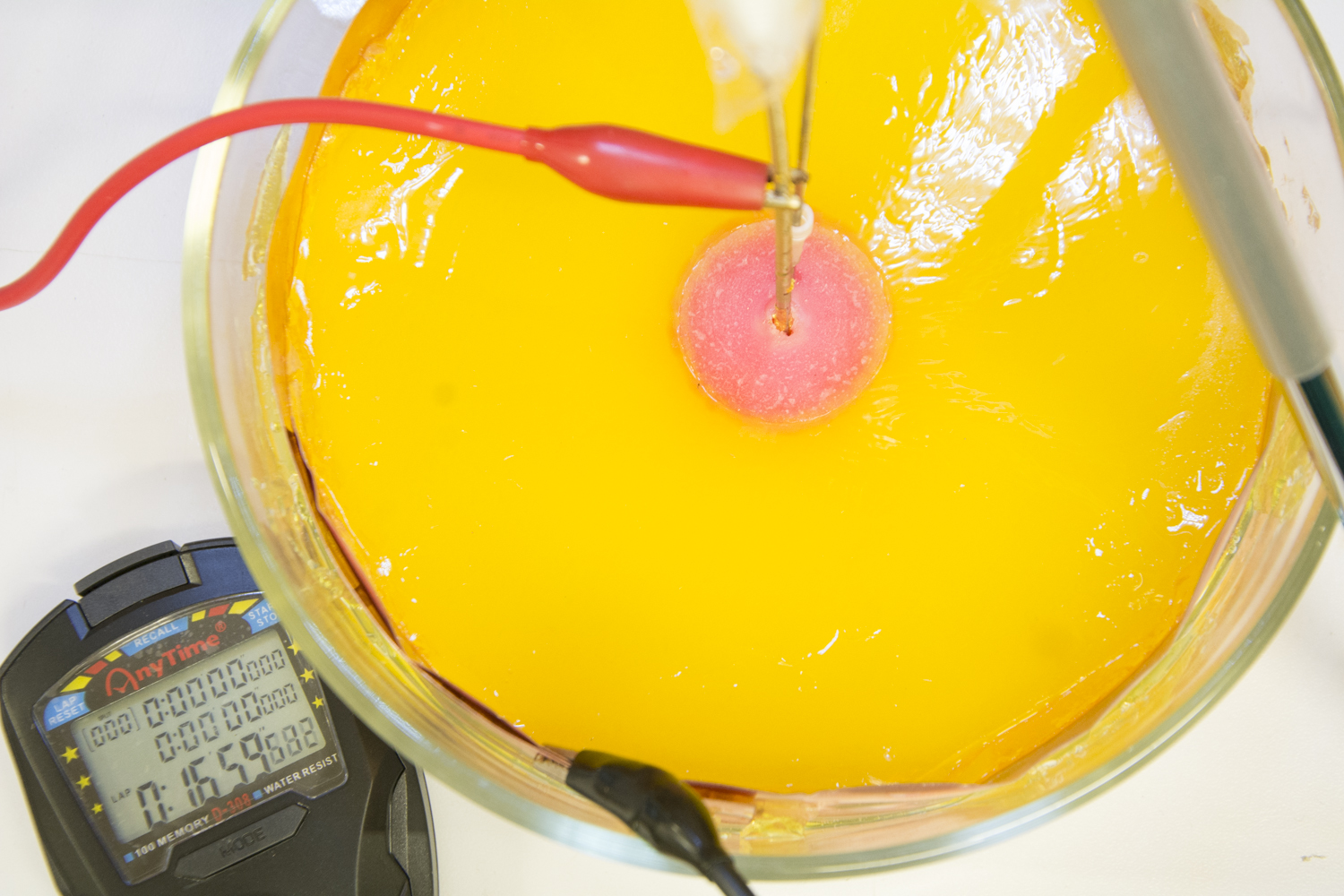

Supplement: Supplemental Information 3 — Photos from 160720-056-Exp_Cryoelettro to 160720-082-Exp_Cryoelettro shows the progression mentioned before from minute 13:00 to minute 26:00; each photo is taken every 30 s. [file peerj-05-2810-s003.zip › PeerJ2/160720-064-Exp_Cryoelettro.jpg]

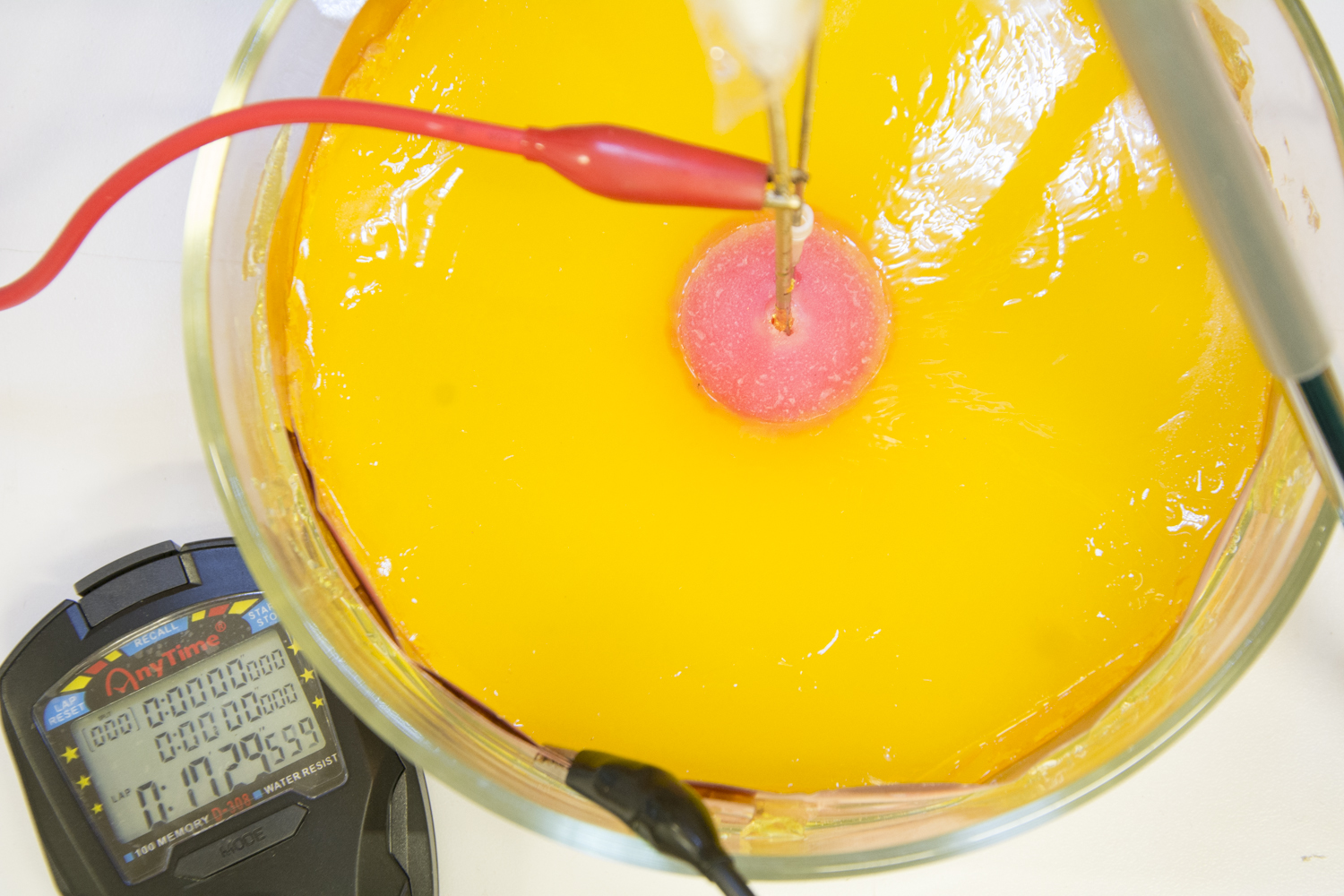

Supplement: Supplemental Information 3 — Photos from 160720-056-Exp_Cryoelettro to 160720-082-Exp_Cryoelettro shows the progression mentioned before from minute 13:00 to minute 26:00; each photo is taken every 30 s. [file peerj-05-2810-s003.zip › PeerJ2/160720-065-Exp_Cryoelettro.jpg]

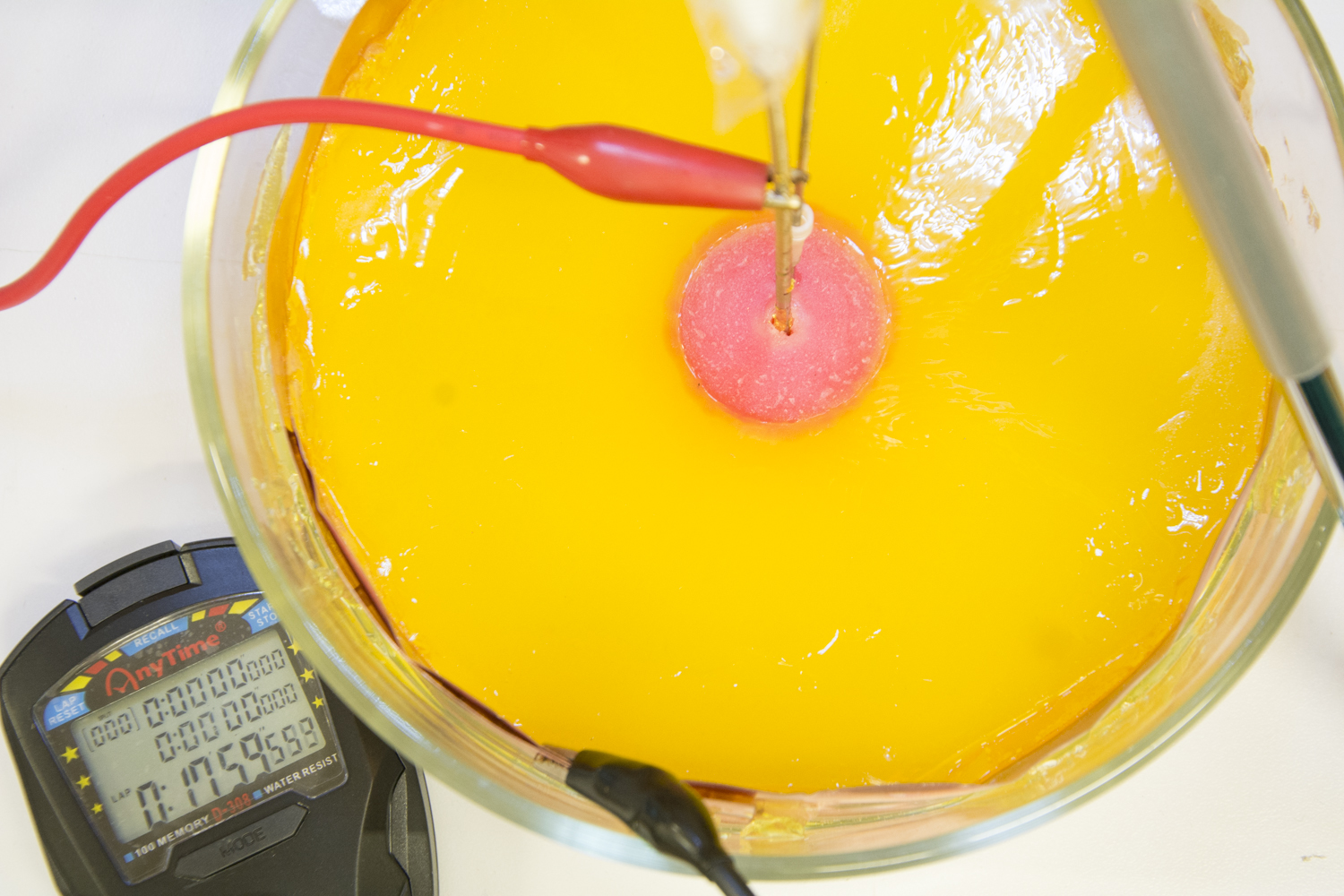

Supplement: Supplemental Information 3 — Photos from 160720-056-Exp_Cryoelettro to 160720-082-Exp_Cryoelettro shows the progression mentioned before from minute 13:00 to minute 26:00; each photo is taken every 30 s. [file peerj-05-2810-s003.zip › PeerJ2/160720-066-Exp_Cryoelettro.jpg]

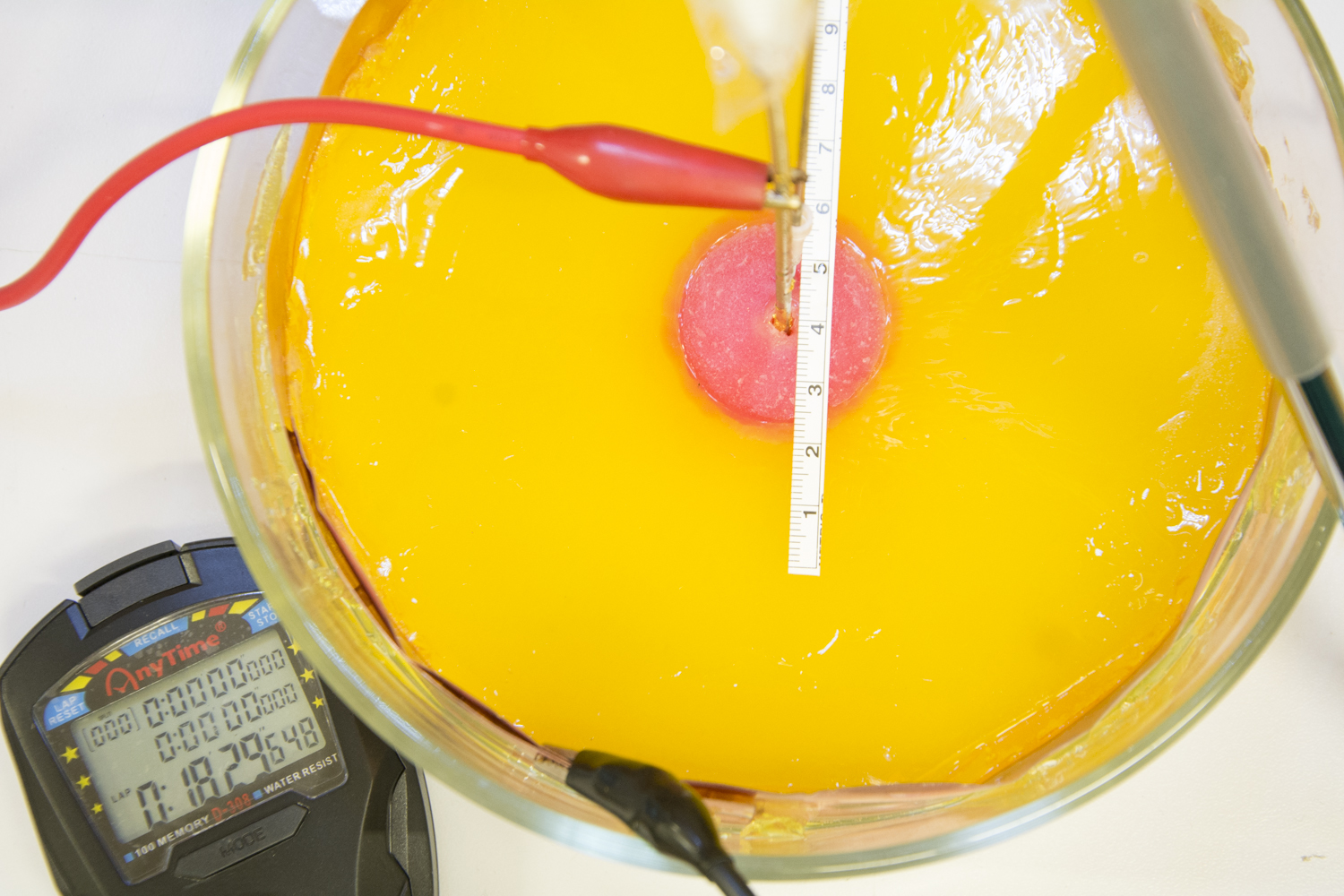

Supplement: Supplemental Information 3 — Photos from 160720-056-Exp_Cryoelettro to 160720-082-Exp_Cryoelettro shows the progression mentioned before from minute 13:00 to minute 26:00; each photo is taken every 30 s. [file peerj-05-2810-s003.zip › PeerJ2/160720-067-Exp_Cryoelettro.jpg]

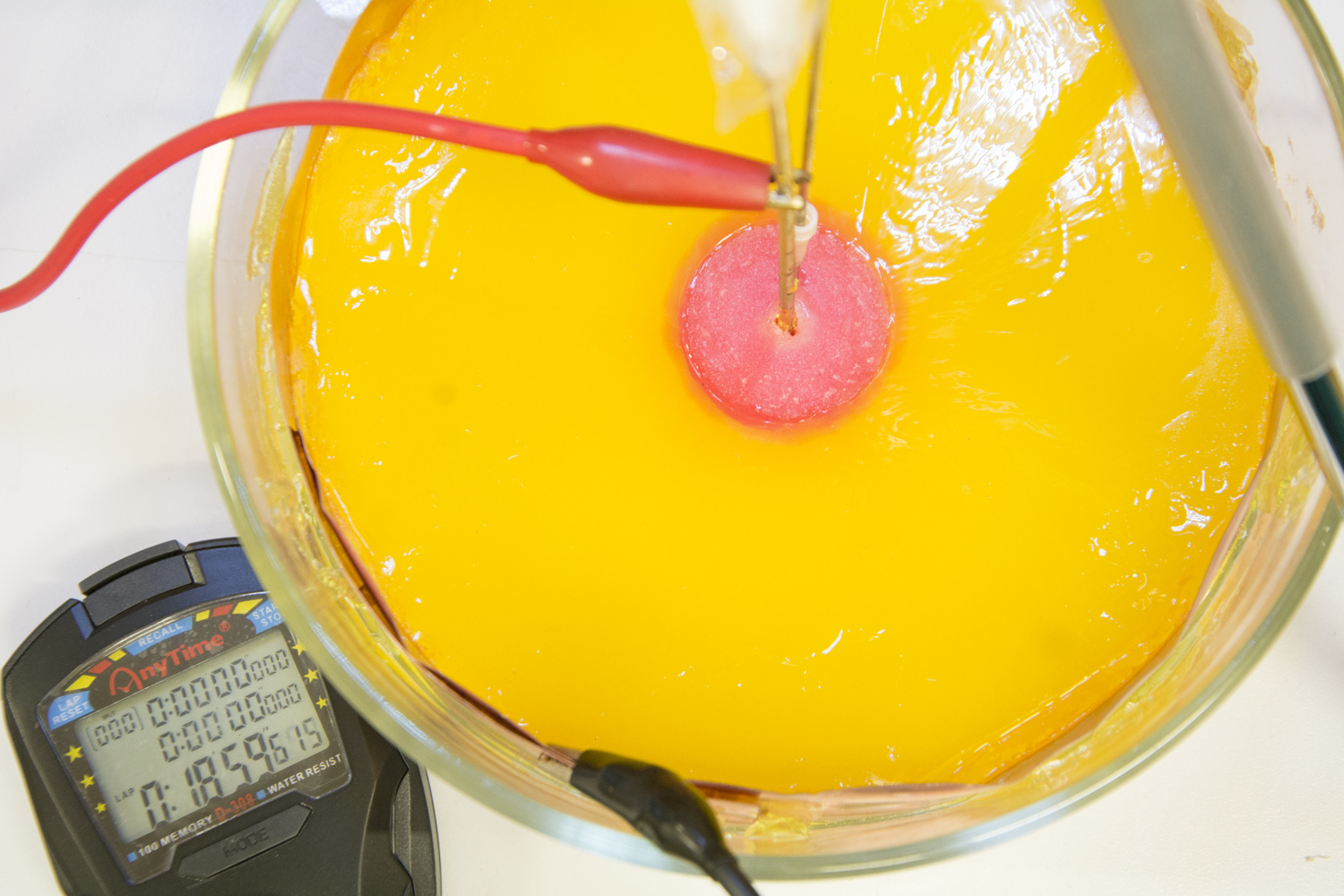

Supplement: Supplemental Information 3 — Photos from 160720-056-Exp_Cryoelettro to 160720-082-Exp_Cryoelettro shows the progression mentioned before from minute 13:00 to minute 26:00; each photo is taken every 30 s. [file peerj-05-2810-s003.zip › PeerJ2/160720-068-Exp_Cryoelettro.jpg]

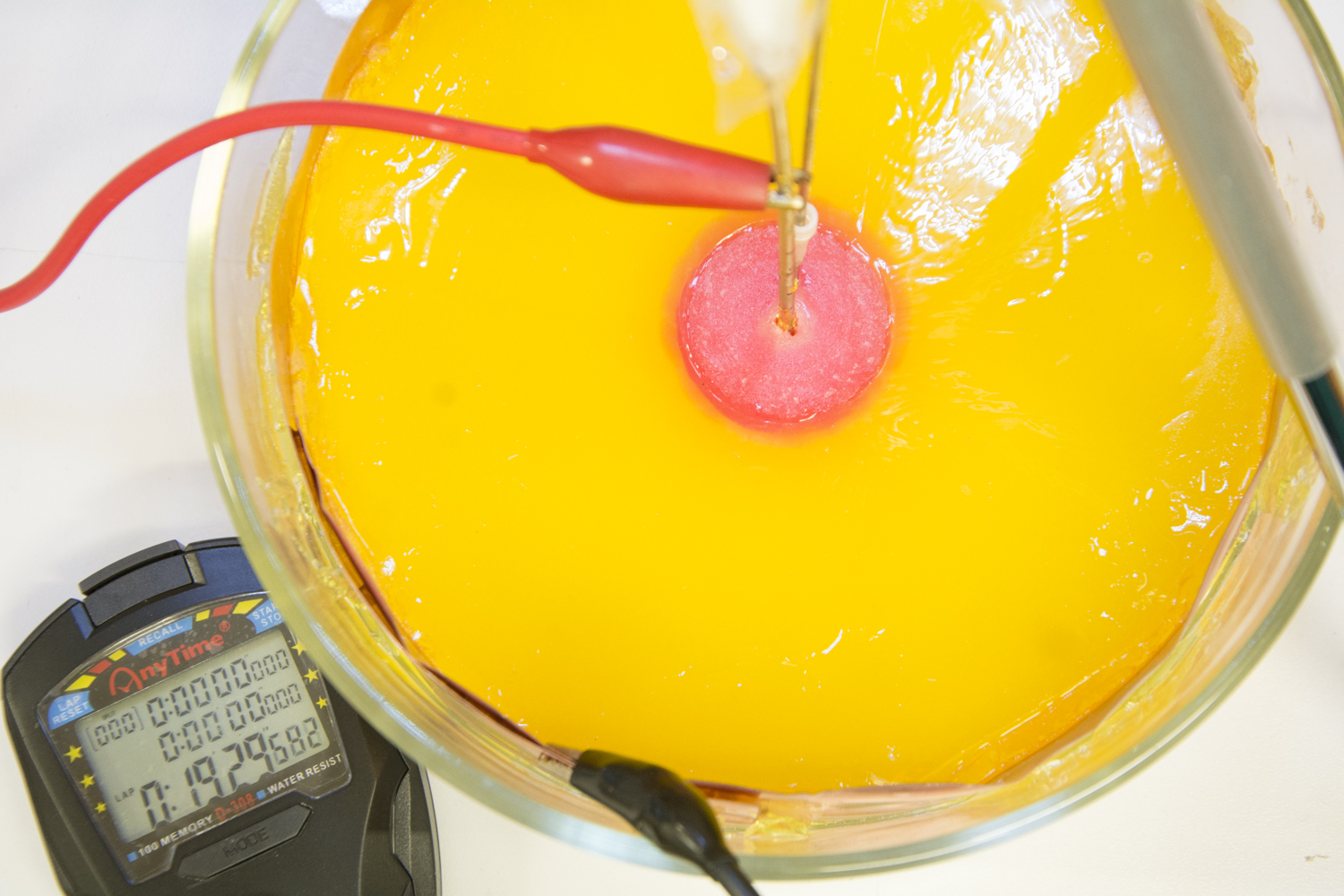

Supplement: Supplemental Information 3 — Photos from 160720-056-Exp_Cryoelettro to 160720-082-Exp_Cryoelettro shows the progression mentioned before from minute 13:00 to minute 26:00; each photo is taken every 30 s. [file peerj-05-2810-s003.zip › PeerJ2/160720-069-Exp_Cryoelettro.jpg]

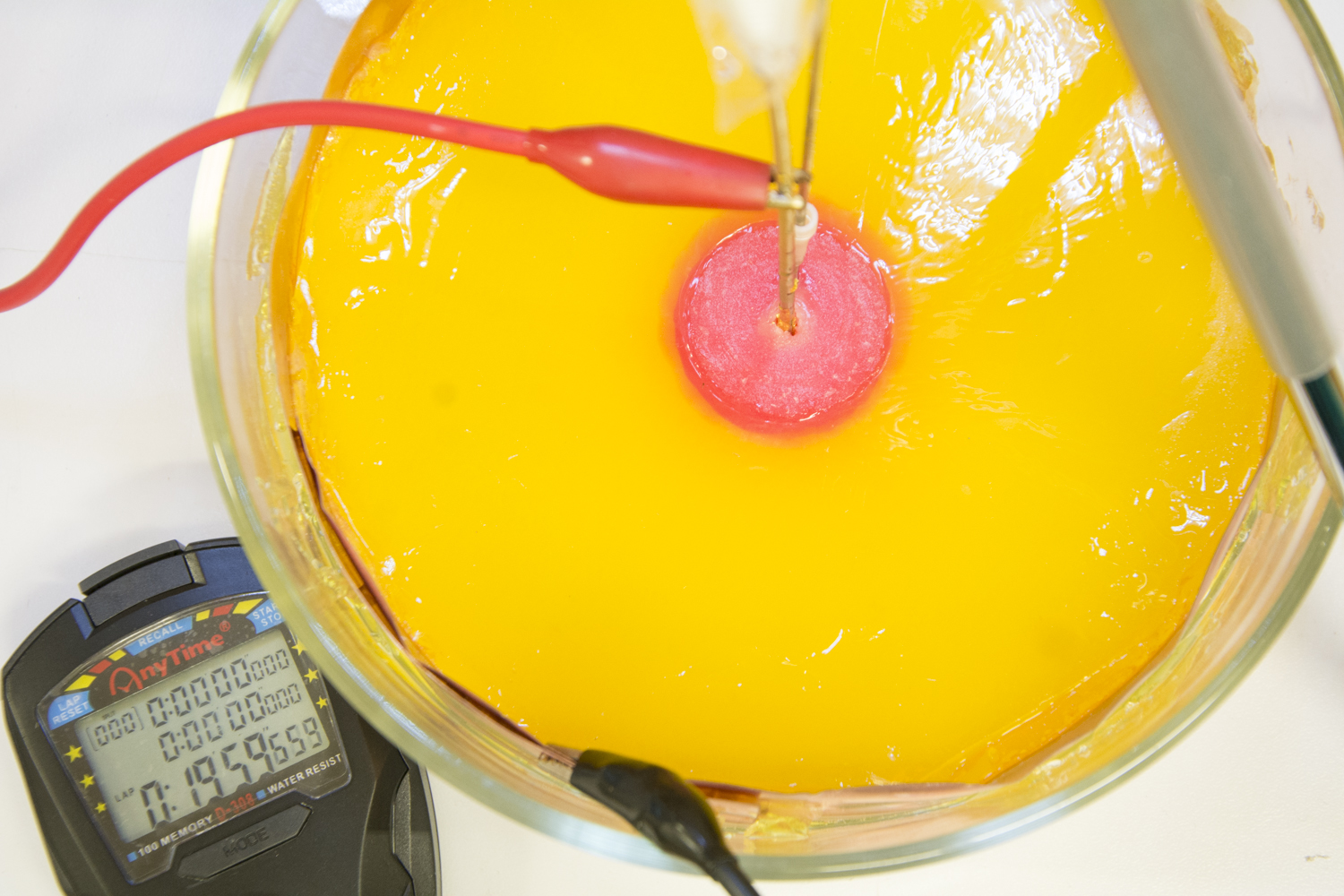

Supplement: Supplemental Information 3 — Photos from 160720-056-Exp_Cryoelettro to 160720-082-Exp_Cryoelettro shows the progression mentioned before from minute 13:00 to minute 26:00; each photo is taken every 30 s. [file peerj-05-2810-s003.zip › PeerJ2/160720-070-Exp_Cryoelettro.jpg]

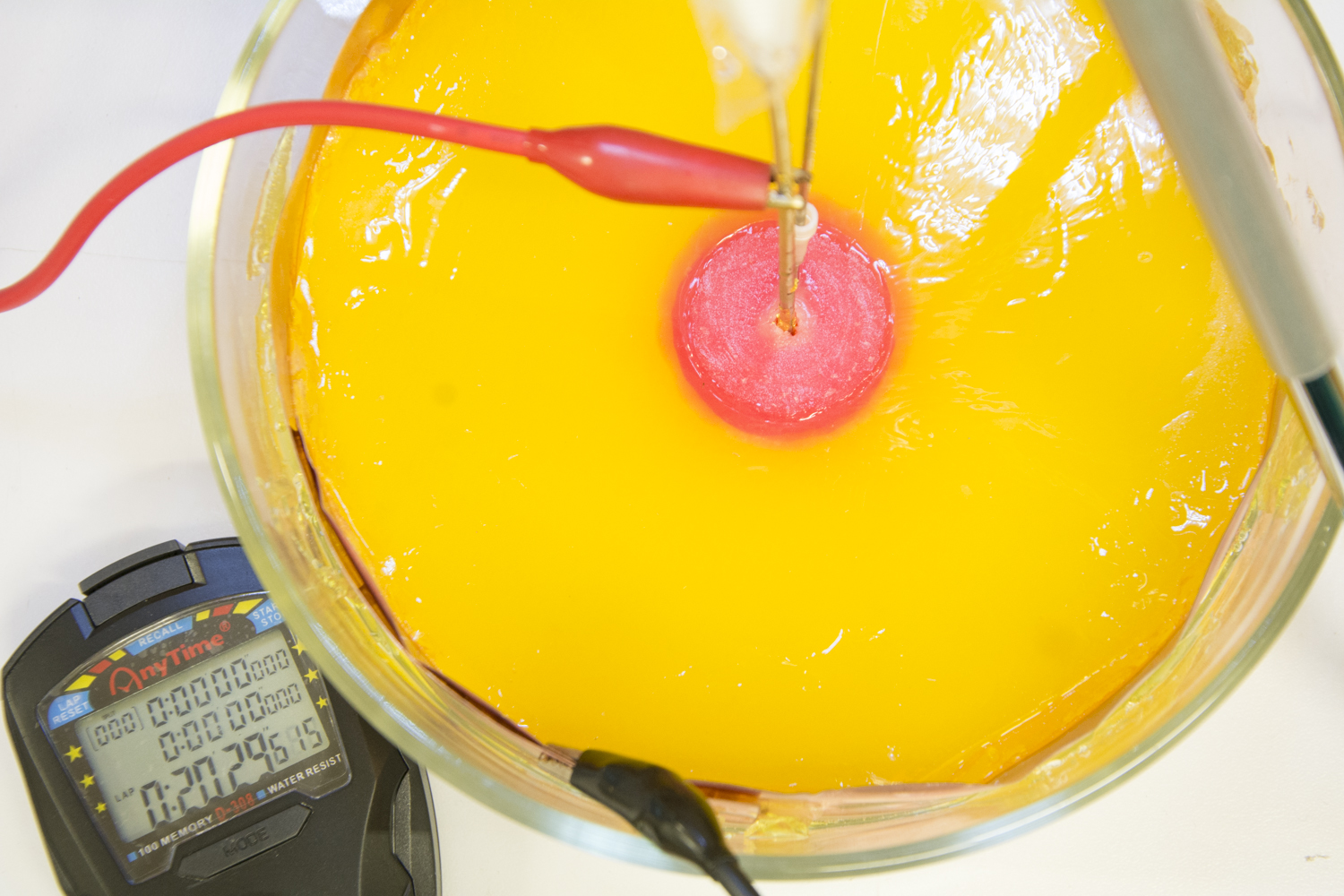

Supplement: Supplemental Information 3 — Photos from 160720-056-Exp_Cryoelettro to 160720-082-Exp_Cryoelettro shows the progression mentioned before from minute 13:00 to minute 26:00; each photo is taken every 30 s. [file peerj-05-2810-s003.zip › PeerJ2/160720-071-Exp_Cryoelettro.jpg]

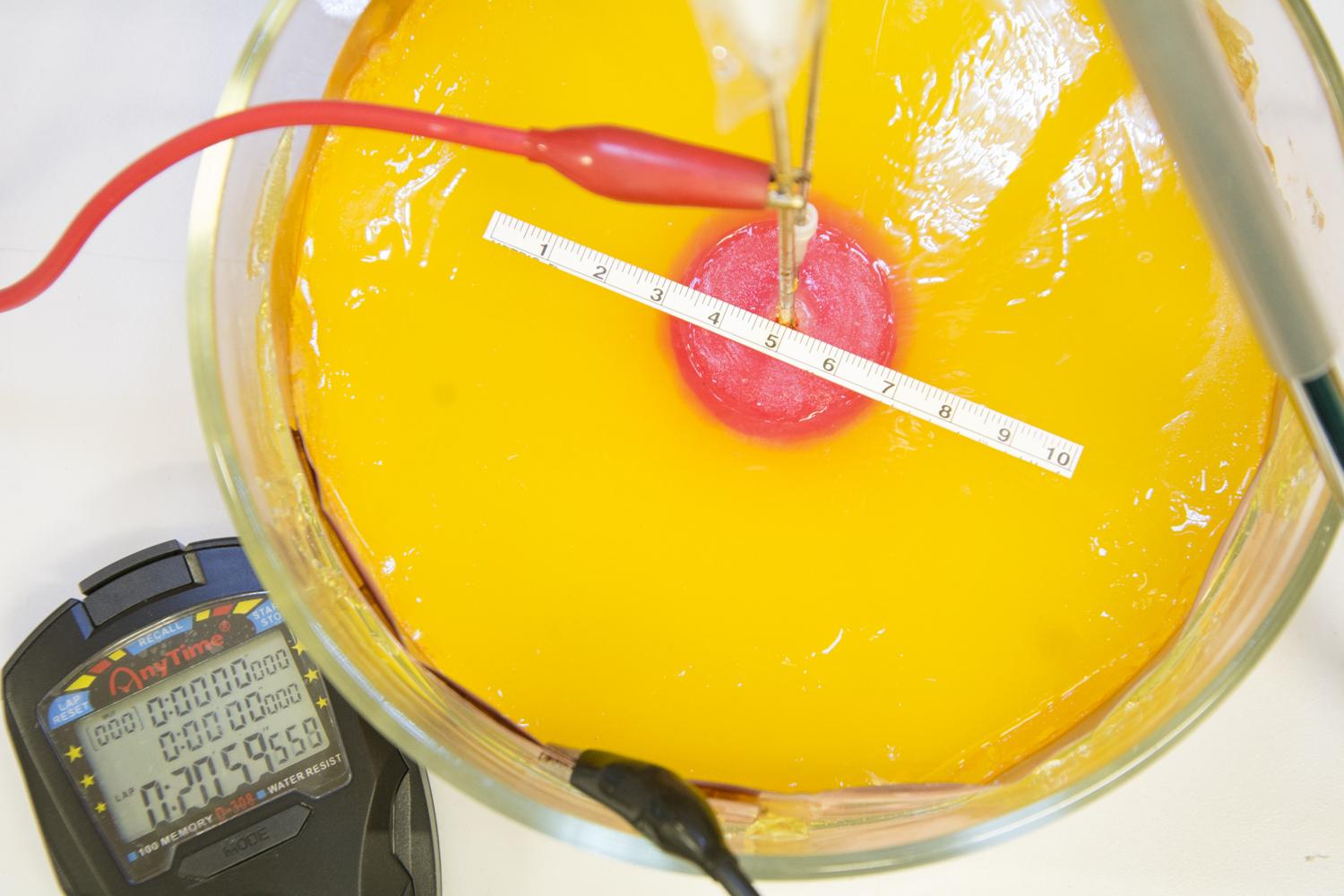

Supplement: Supplemental Information 3 — Photos from 160720-056-Exp_Cryoelettro to 160720-082-Exp_Cryoelettro shows the progression mentioned before from minute 13:00 to minute 26:00; each photo is taken every 30 s. [file peerj-05-2810-s003.zip › PeerJ2/160720-072-Exp_Cryoelettro.jpg]

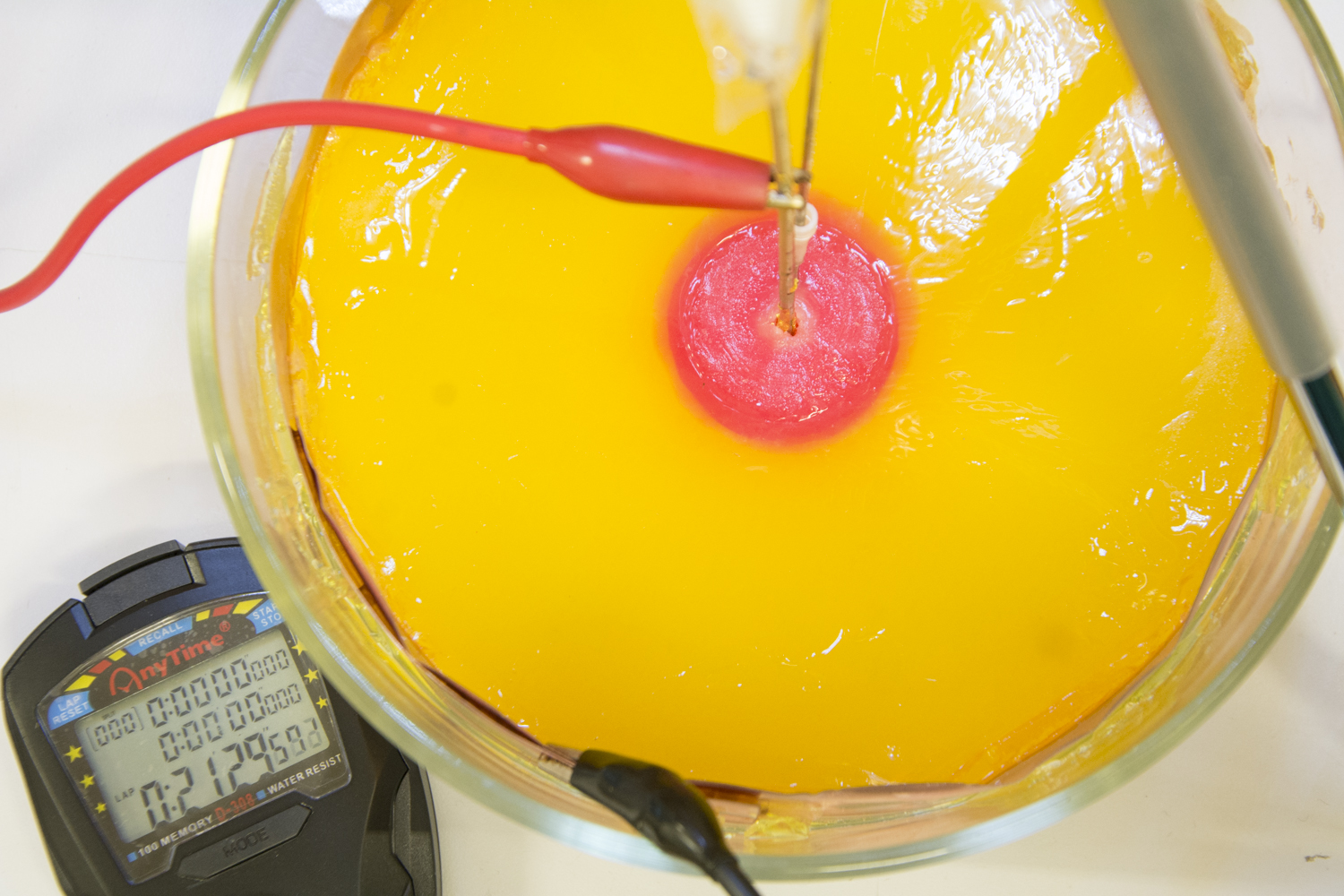

Supplement: Supplemental Information 3 — Photos from 160720-056-Exp_Cryoelettro to 160720-082-Exp_Cryoelettro shows the progression mentioned before from minute 13:00 to minute 26:00; each photo is taken every 30 s. [file peerj-05-2810-s003.zip › PeerJ2/160720-073-Exp_Cryoelettro.jpg]

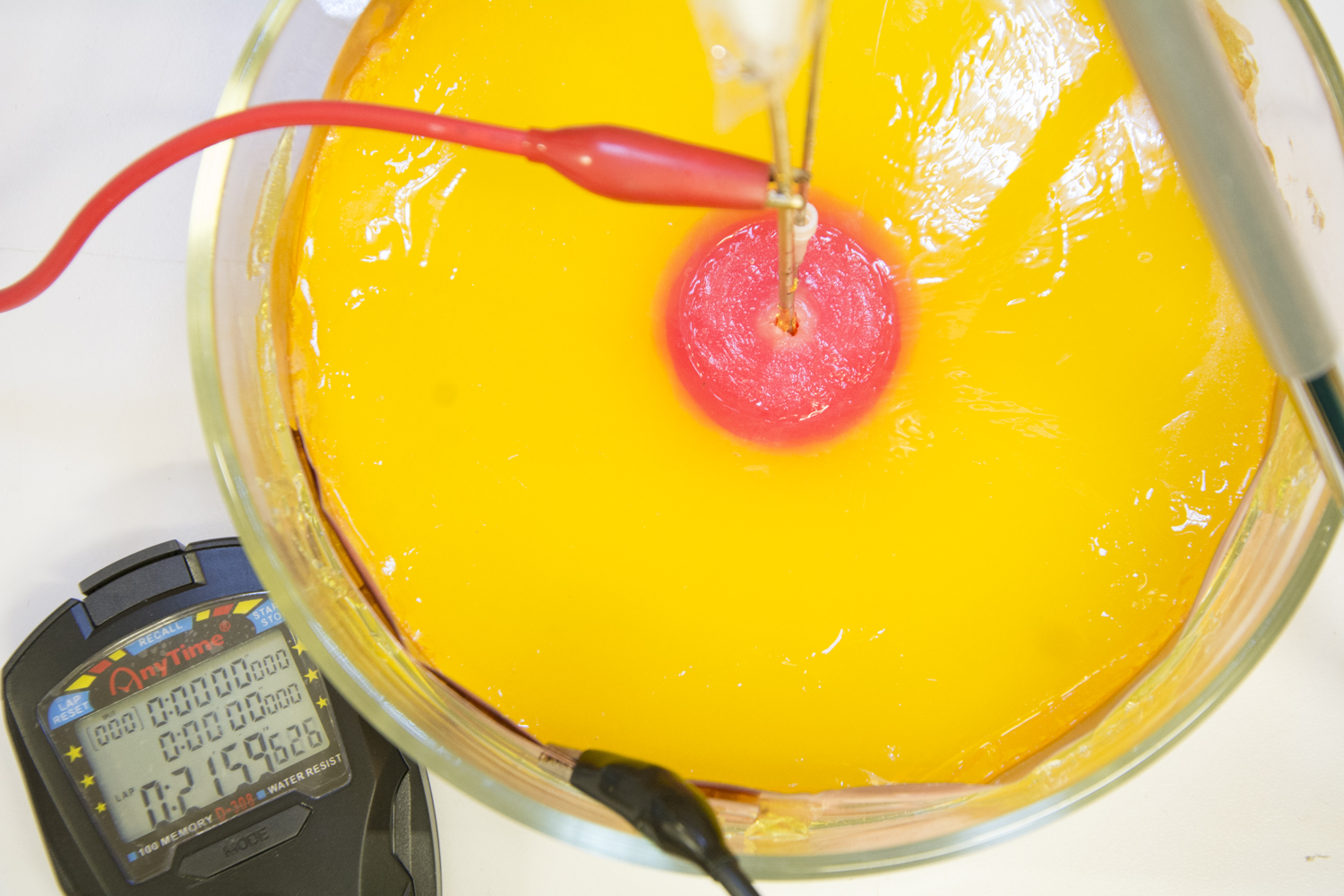

Supplement: Supplemental Information 3 — Photos from 160720-056-Exp_Cryoelettro to 160720-082-Exp_Cryoelettro shows the progression mentioned before from minute 13:00 to minute 26:00; each photo is taken every 30 s. [file peerj-05-2810-s003.zip › PeerJ2/160720-074-Exp_Cryoelettro.jpg]

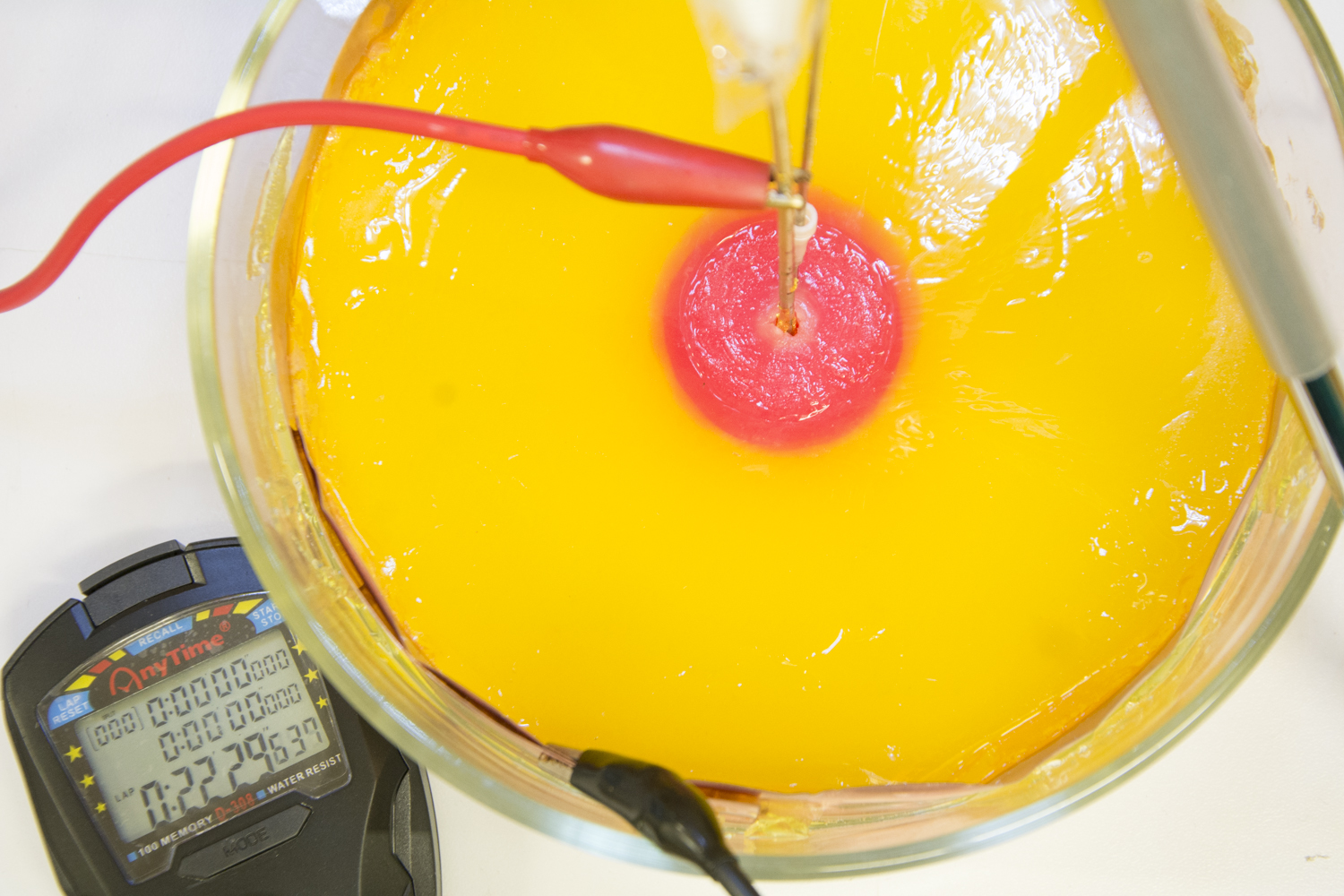

Supplement: Supplemental Information 3 — Photos from 160720-056-Exp_Cryoelettro to 160720-082-Exp_Cryoelettro shows the progression mentioned before from minute 13:00 to minute 26:00; each photo is taken every 30 s. [file peerj-05-2810-s003.zip › PeerJ2/160720-075-Exp_Cryoelettro.jpg]

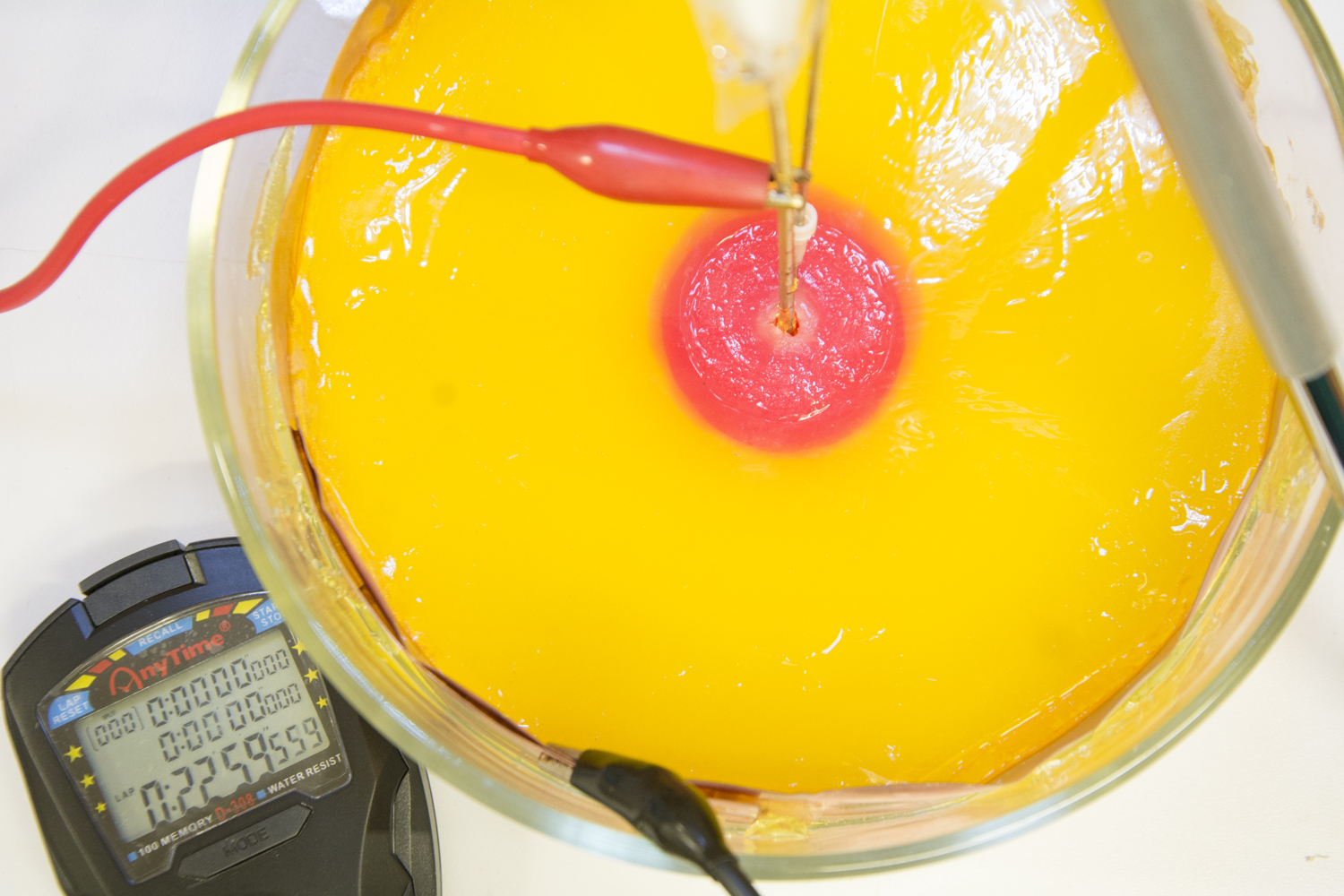

Supplement: Supplemental Information 3 — Photos from 160720-056-Exp_Cryoelettro to 160720-082-Exp_Cryoelettro shows the progression mentioned before from minute 13:00 to minute 26:00; each photo is taken every 30 s. [file peerj-05-2810-s003.zip › PeerJ2/160720-076-Exp_Cryoelettro.jpg]

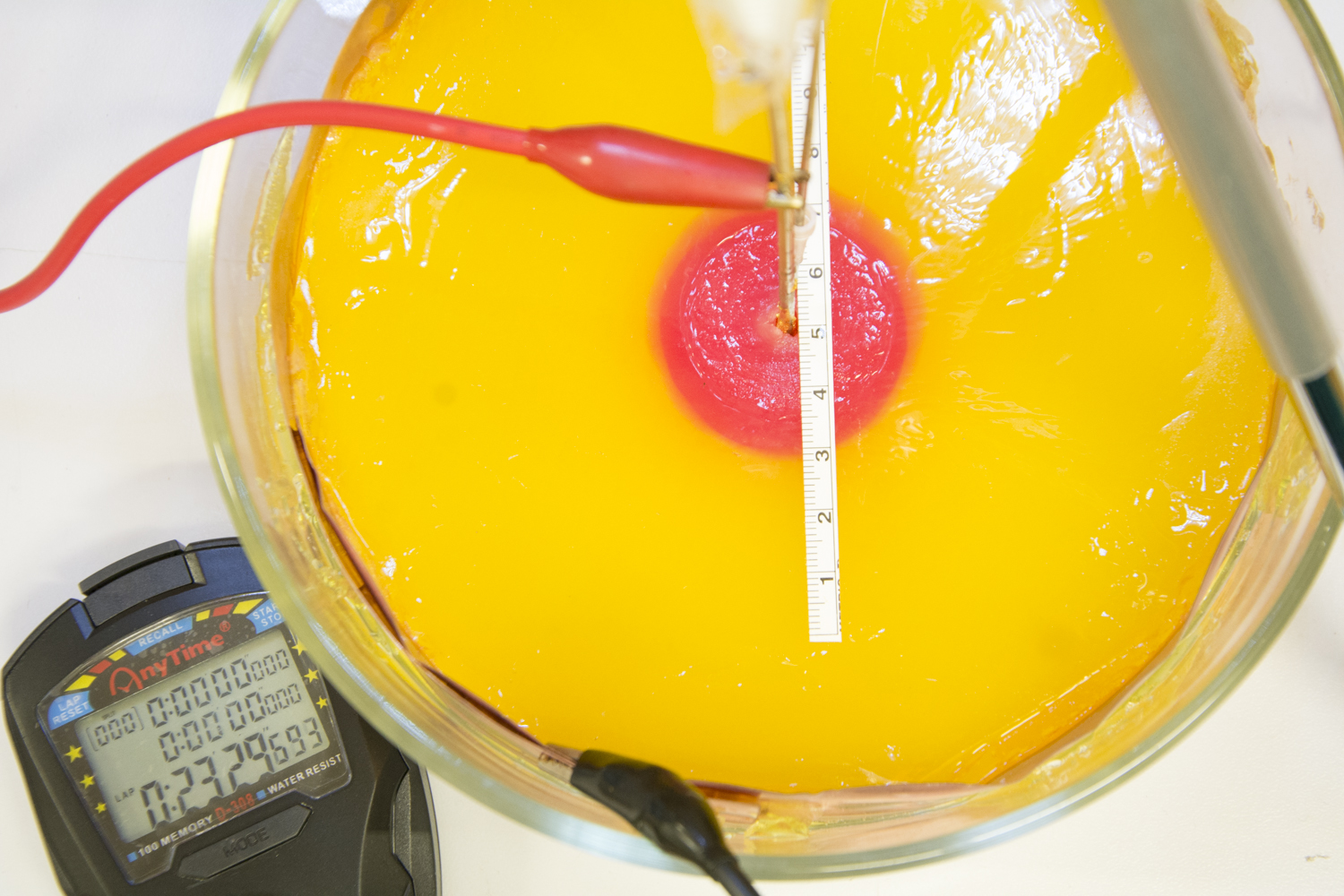

Supplement: Supplemental Information 3 — Photos from 160720-056-Exp_Cryoelettro to 160720-082-Exp_Cryoelettro shows the progression mentioned before from minute 13:00 to minute 26:00; each photo is taken every 30 s. [file peerj-05-2810-s003.zip › PeerJ2/160720-077-Exp_Cryoelettro.jpg]

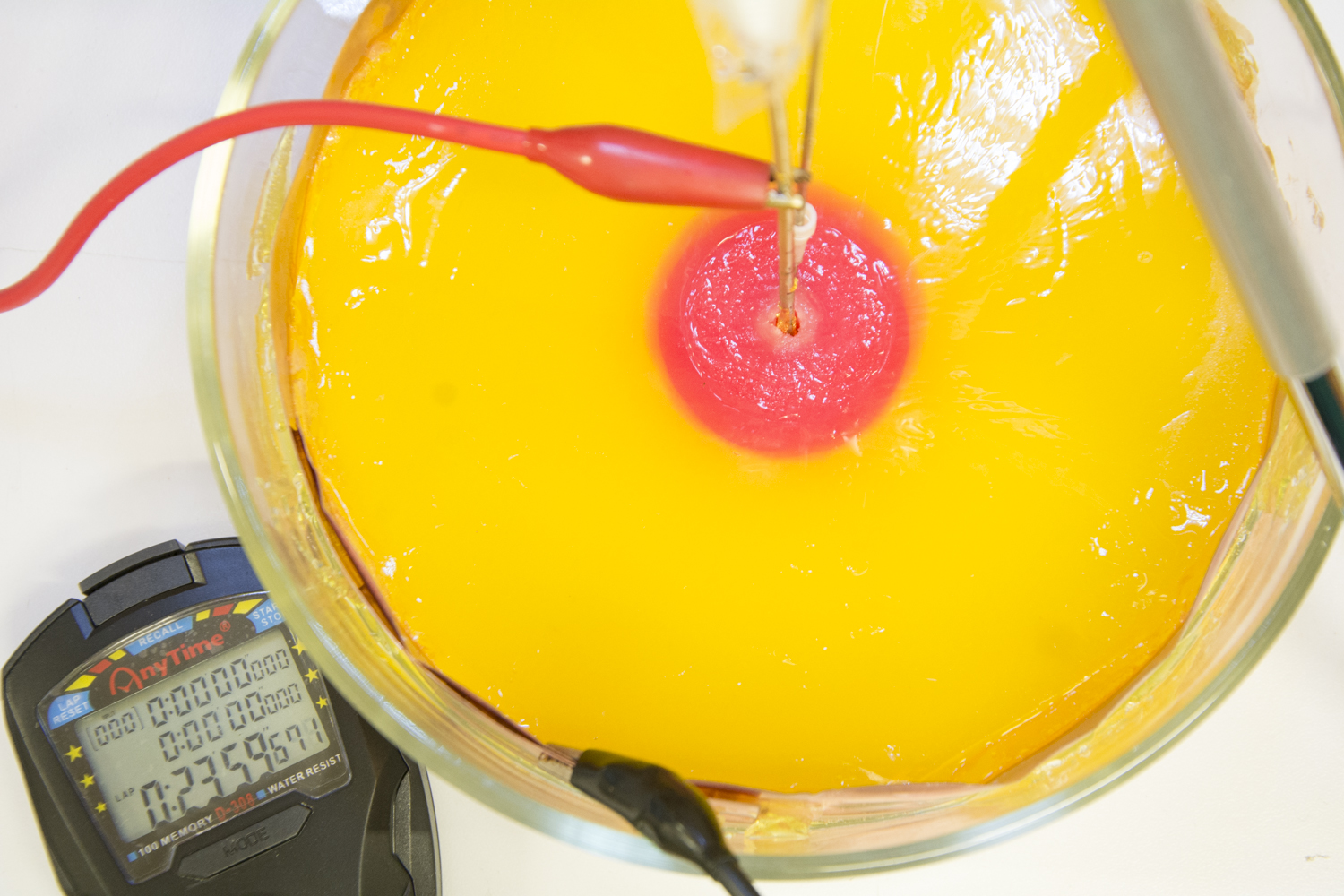

Supplement: Supplemental Information 3 — Photos from 160720-056-Exp_Cryoelettro to 160720-082-Exp_Cryoelettro shows the progression mentioned before from minute 13:00 to minute 26:00; each photo is taken every 30 s. [file peerj-05-2810-s003.zip › PeerJ2/160720-078-Exp_Cryoelettro.jpg]

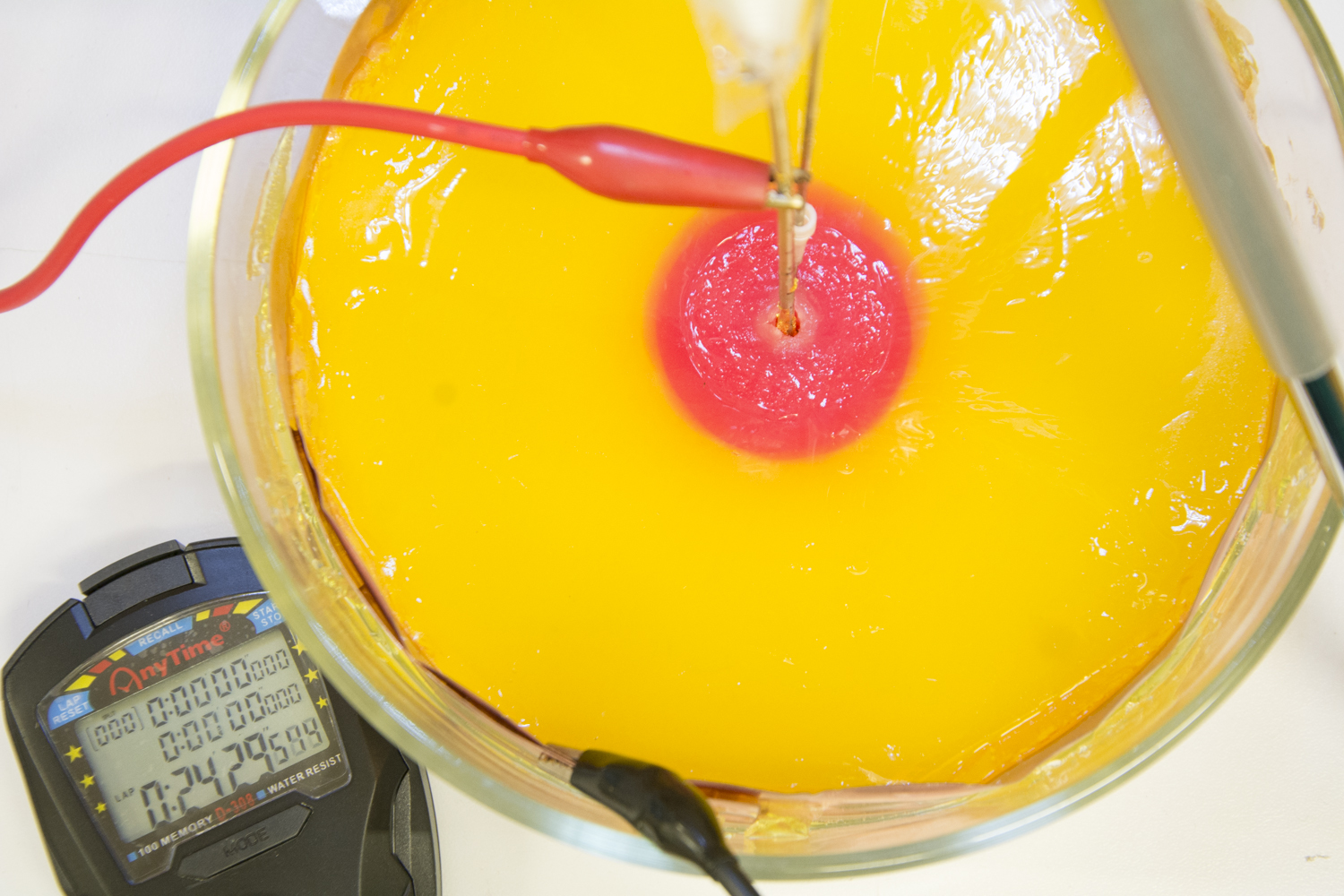

Supplement: Supplemental Information 3 — Photos from 160720-056-Exp_Cryoelettro to 160720-082-Exp_Cryoelettro shows the progression mentioned before from minute 13:00 to minute 26:00; each photo is taken every 30 s. [file peerj-05-2810-s003.zip › PeerJ2/160720-079-Exp_Cryoelettro.jpg]

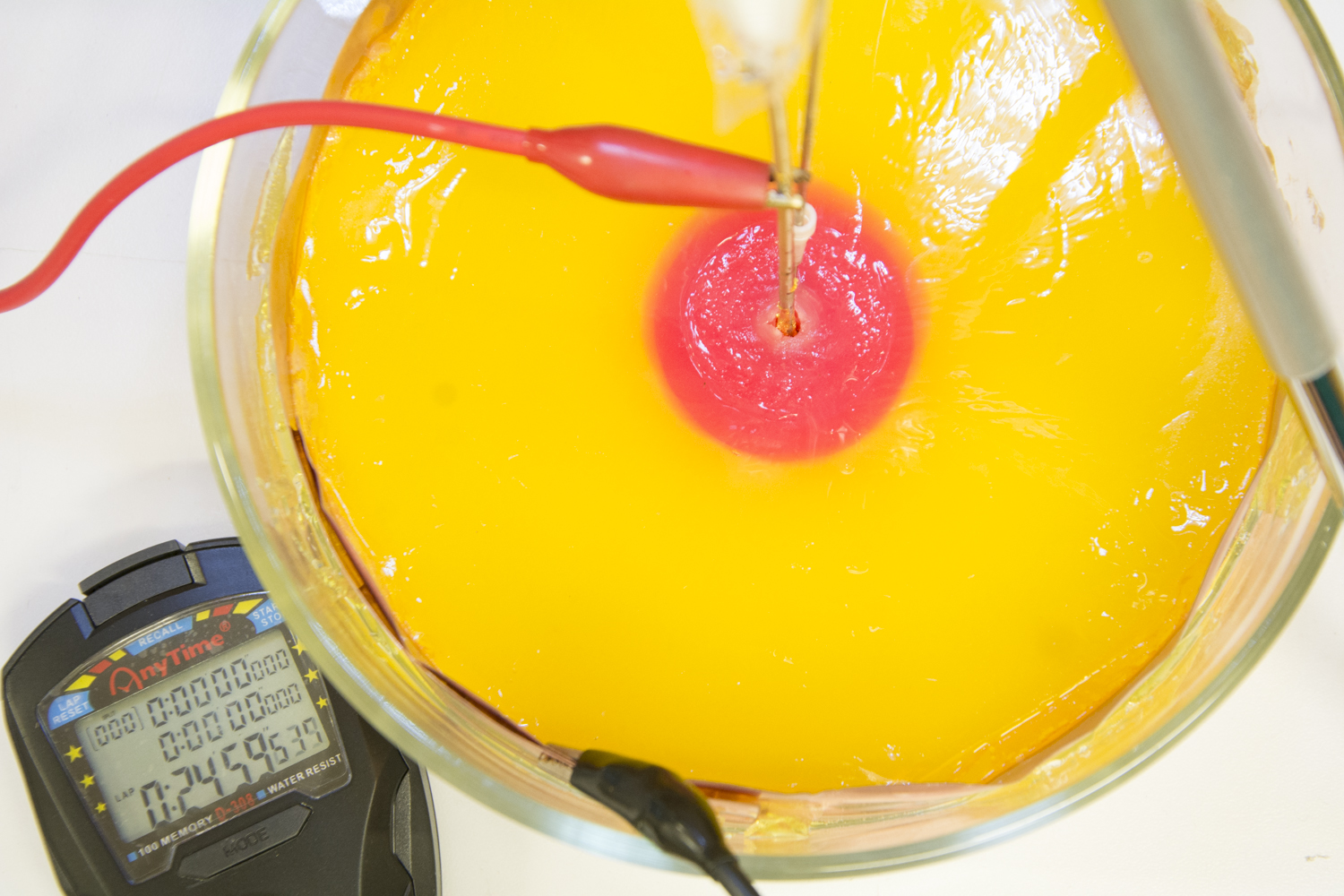

Supplement: Supplemental Information 3 — Photos from 160720-056-Exp_Cryoelettro to 160720-082-Exp_Cryoelettro shows the progression mentioned before from minute 13:00 to minute 26:00; each photo is taken every 30 s. [file peerj-05-2810-s003.zip › PeerJ2/160720-080-Exp_Cryoelettro.jpg]

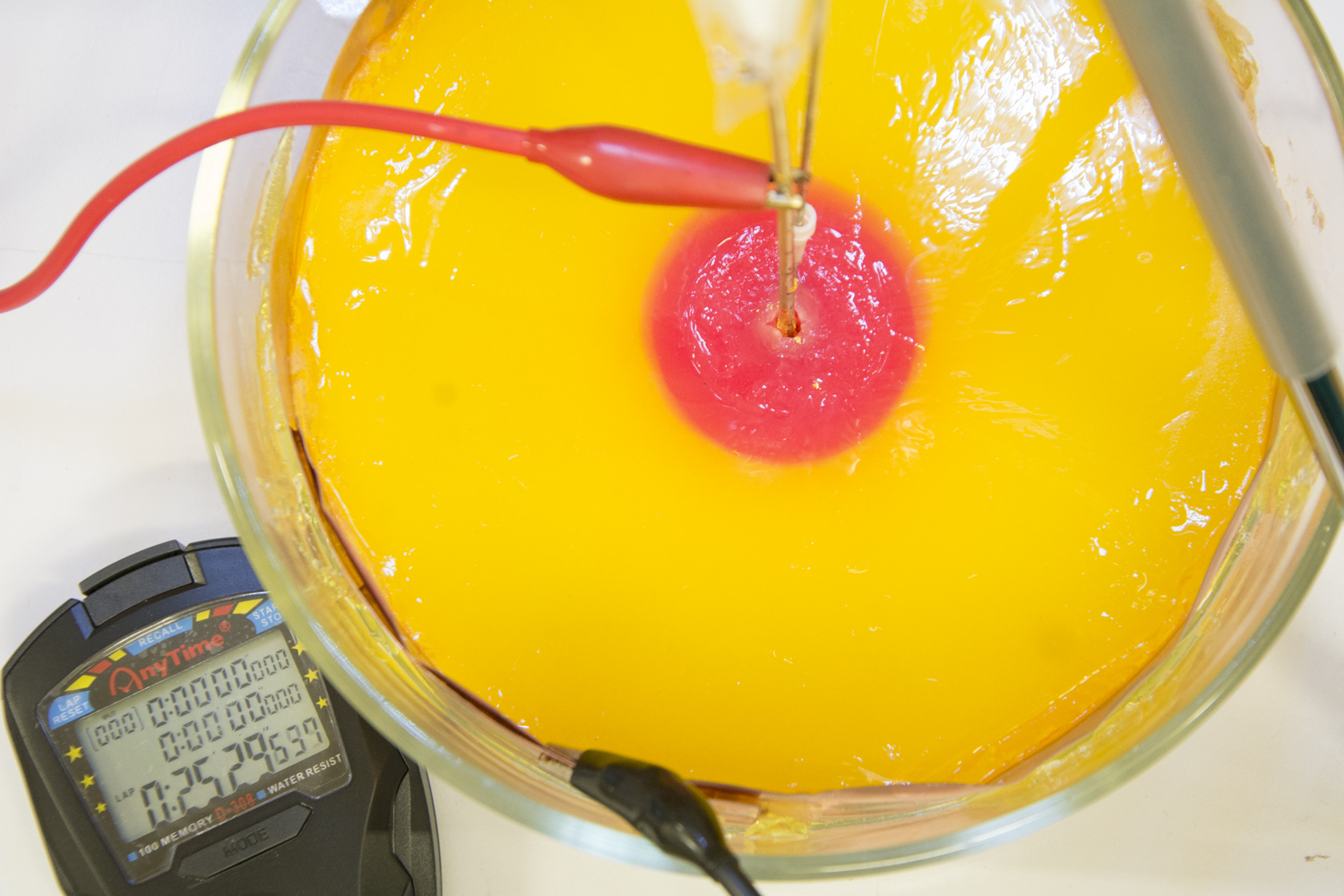

Supplement: Supplemental Information 3 — Photos from 160720-056-Exp_Cryoelettro to 160720-082-Exp_Cryoelettro shows the progression mentioned before from minute 13:00 to minute 26:00; each photo is taken every 30 s. [file peerj-05-2810-s003.zip › PeerJ2/160720-081-Exp_Cryoelettro.jpg]

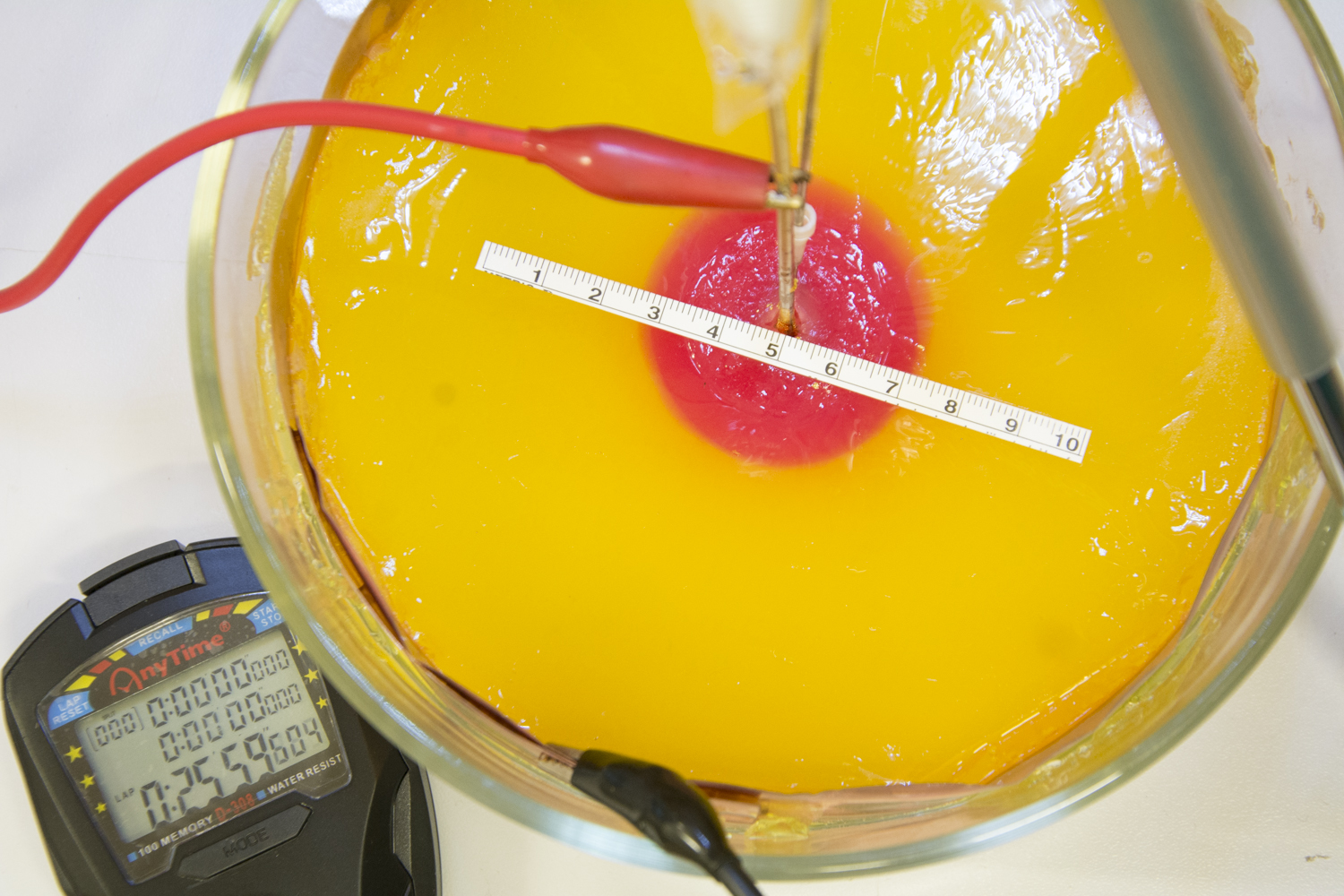

Supplement: Supplemental Information 3 — Photos from 160720-056-Exp_Cryoelettro to 160720-082-Exp_Cryoelettro shows the progression mentioned before from minute 13:00 to minute 26:00; each photo is taken every 30 s. [file peerj-05-2810-s003.zip › PeerJ2/160720-082-Exp_Cryoelettro.jpg]
